# A Pandemic-Scale Ancestral Recombination Graph for SARS-CoV-2

**DOI:** 10.1101/2023.06.08.544212

**Published:** 2025-11-10

**Authors:** Shing H. Zhan, Yan Wong, Anastasia Ignatieva, Katherine Eaton, Isobel Guthrie, Benjamin Jeffery, Duncan S. Palmer, Carmen Lia Murall, Sarah P. Otto, Jerome Kelleher

**Affiliations:** 1 Big Data Institute, Li Ka Shing Centre for Health Information and Discovery, University of Oxford, United Kingdom; 2 Infectious Disease Epidemiology Unit (IDEU), Nuffield Department of Population Health, University of Oxford, Oxford, United Kingdom; 3 Department of Statistics, University of Oxford, United Kingdom; 4 National Microbiology Laboratory, Public Health Agency of Canada, Canada; 5 The Pioneer Centre for SMARTbiomed, Big Data Institute, Li Ka Shing Centre for Health Information and Discovery, University of Oxford, United Kingdom; 6 Department of Zoology and Biodiversity Research Centre, University of British Columbia, Vancouver BC Canada; 7 Joint second author; 8 Lead contact

**Keywords:** Ancestral recombination graphs, recombination, SARS-CoV-2, ARG, tskit

## Abstract

Millions of SARS-CoV-2 genome sequences were collected during the COVID-19 pandemic, forming a dataset of unprecedented richness. Estimated genealogies are fundamental to understanding this ocean of data and form the primary input to many downstream analyses. A basic assumption of methods to infer genealogies from viral genetic data is that recombination is negligible and the genealogy is a tree. However, recombinant lineages have risen to global prevalence, and simple tree representations are therefore incomplete and potentially misleading. We present sc2ts, a method to infer reticulate genealogies as an Ancestral Recombination Graph (ARG) in real time at pandemic scale. We infer an ARG for 2.48 million SARS-CoV-2 genomes, which leverages the widely used tskit software ecosystem to support further analyses and visualisation. This rich and validated resource clarifies the relationships among recombinant lineages, quantifies the rate of recombination over time, and provides a lower bound on detectable recombination.

## INTRODUCTION

Classical phylogenetics is largely concerned with inferring the evolutionary history of divergent species^1^. Recombination among lineages, however, distorts the inference of species trees^2^, biases downstream analyses^3,4^, and so requires careful consideration^5^. Modern pathogen datasets are not sparsely sampled from different species and may contain millions of genomes sampled from a single species^6–10^. In contrast to the sophisticated model-based approaches used to infer species trees^11–13^, comprehensive phylogenies of pathogen datasets are typically built with simpler distance methods or approximations^14^, primarily due to computational scaling limitations. While several methods have been proposed to incorporate recombination explicitly into model-based phylogenetic inference^15–19^, scalability is a major issue and practical application has been limited. Software support that explicitly *uses* recombination-aware inference is mostly restricted to visualisation, for example of small-scale phylogenetic networks^20–22^ or tree changes along the genome^23^.

The global surveillance data collected during the COVID-19 pandemic presents fundamental challenges to existing approaches. First, the sheer amount of data overwhelmed even approximate distance matrix-based methods. At the peak in February 2022, ~80,000 samples per day were submitted to GISAID^6^, accumulating to a total of over 17.5 million samples as of October 2025. Regularly re-inferring trees de novo from such rapidly growing datasets is untenable. The UShER^24^ method is based on adding samples to an existing tree using maximum parsimony, with periodic rearrangements to counter the inherent greediness of the approach^25^. The benefits of this simple model and the value of a comprehensive genealogy updated in real time^26,27^ were conclusively demonstrated, as the resource quickly became an indispensable element of the pandemic response^28^. Recurrent recombination has posed an additional challenge to reconstructing the evolutionary history of SARS-CoV-2^29–34^, with multiple deep recombination events impacting both the genealogy and disease attributes of the virus^35,36^. Although several methods have been proposed to *detect* recombinant samples^37–41^, this information is not incorporated into the phylogenies used in innumerable studies^26,42^, and recombination continues to be treated in a post hoc manner, after phylogenetic reconstruction.

Ancestral recombination graphs (ARGs) describe the reticulate genealogies of sampled sequences in a recombining species^43^, and provide a natural and efficient means of systematically incorporating recombination into the study of SARS-CoV-2. While ARGs have been of theoretical interest in population genetics for several decades^44–46^, recent breakthroughs in simulation^47–50^ and inference^51–57^ have led to a surge of interest in their application in population and statistical genetics^58–60^. As the range of applications for ARGs has broadened, the term has itself evolved from a specific model of coalescence with recombination^44,46^to a rich data structure representing recombinant ancestry^43,61^. The development of extensive software support has facilitated the broader application of ARGS. In particular, the collaboratively built tskit library^62^ underpins a rapidly growing software ecosystem of simulation^49,63–66^, visualisation^67–69^, inference^53,54,70–72^ and data analysis^73–75^ tools.

Here we present sc2ts, a new method to infer ARGs for SARS-CoV-2 that sequentially adds samples over time in a similar manner to UShER, while automatically detecting and incorporating recombination in real-time. Based on the freely-available Viridian dataset, a curated collection of SARS-CoV-2 genomes purged of various systematic errors and artifacts^76^, we infer an ARG with 2.48 million whole genome sequences. We show that the non-recombinant portions of this ARG are congruent with the UShER phylogeny built from the same dataset as well as the Pango nomenclature system^77^. The ARG also accurately captures evolutionary features such as mutational spectra^78^ and deletions^79^. We show that sc2ts automatically and accurately detects details about known recombinant lineages that were painstakingly collated through a community effort and provides novel insights into the relationships between these recombinant lineages. We compare the recombination events detected by sc2ts with the state-of-the-art combination of UShER and RIPPLES^38^, showing that sc2ts is more sensitive, detecting more of the well-characterized recombination events curated by the Pango lineage designation community. The output of sc2ts—incorporating genealogical relationships, point mutations, deletions, and recombination—provides a basis for the study of SARS-CoV-2 that systematically accounts for all of these evolutionary forces. This ARG, coupled with the rich suite of supporting software in the tskit^62^ and VCF-Zarr^80^ ecosystems, enables pandemic-scale analysis on a laptop. These resources provide a strong platform for future research, helping us to learn from the vast troves of data collected during the pandemic and to prepare for the next.

## RESULTS

### Overview of sc2ts

**Figure 1:**
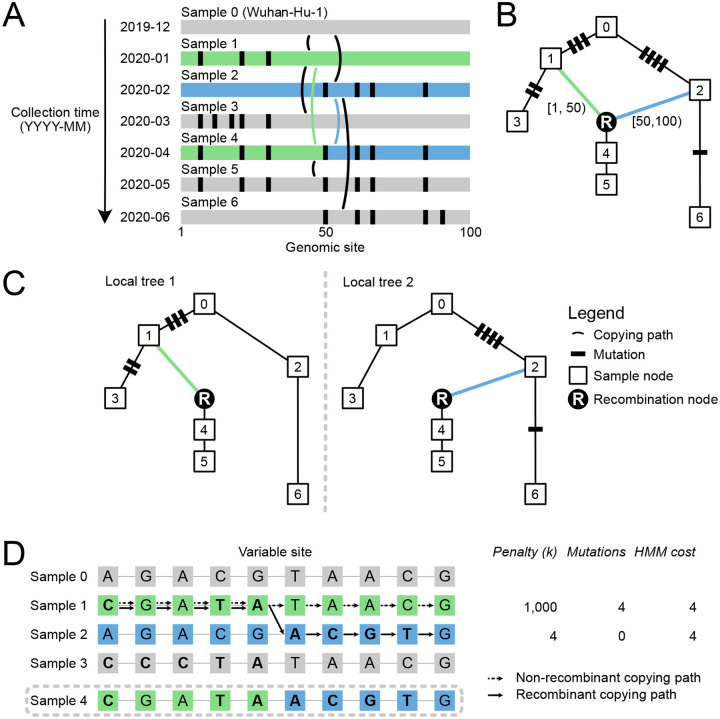
Schematic overview of sc2ts. (A) Process to build an ARG by incrementally updating it with samples sorted by collection dates. (B) The ARG resulting from the inference process. Sample 0 is used to initialize the ARG as the root. Next, Sample 1 is attached, mapping mutations onto the new branch to explain it. When Sample 4 is detected as a recombinant, a node representing the recombination event is inserted with two edges connecting to its parents, with Sample 4 itself attached to this node as its child. (C) Two local trees contained in the resulting ARG, one on each side of the inferred breakpoint which occurs at position 50. The genomic coordinates of the segments inherited from different recombinant parents are denoted by two half-open intervals. (D) Copying paths for Sample 4 inferred under the LS model while allowing recombination (*k* = 4) or disallowing it (*k* = 1000).

Sc2ts is a real-time inference method, updated incrementally with daily batches of sequence data (Figure 1). For each batch, sc2ts first infers likely “copying paths” connecting each sample to nodes in the current ARG using a simplified version of the Li and Stephens (LS) model^81^. The LS model is a Hidden Markov Model (HMM) widely used in human genomics to approximate the effects of mutation and recombination on genetic inheritance^82,83^, typically parametrised by population-scaled mutation and recombination rates varying along the genome^84^. In the interest of interpretability, we simplified this model to use a single parameter *k* that controls the number of recurrent mutations that is allowed before switching to a different parent via recombination (we use *k* = 4 here; see STAR Methods). We used the highly efficient LS HMM implementation in tsinfer^53^, with some additional optimisations enabled by our simplified model, to find exact Viterbi solutions of these copying paths for each sample given the current ARG. Note that this is a form of parsimony, with the recombination penalty *k* defining the relative parsimony of recombinant versus non-recombinant solutions to the HMM. The per-sample copying paths produced by the HMM (most of which do not involve recombination) form clusters of samples within a batch, which are then resolved using standard phylogenetic techniques. Finally, sc2ts attaches the phylogenies of the clusters to the current ARG, and applies parsimony-improving heuristics to address issues introduced by the inherent greediness of this strategy. Sc2ts is implemented in Python, building on core PyData infrastructure^85–88^ and bioinformatics packages^89–91^. See STAR Methods for details on all aspects of the method.

### A comprehensive representation of SARS-CoV-2 evolution

Using sc2ts, we built an ARG from 2,482,157 samples from the Viridian v04 dataset^76^, collected between 2020-01-01 and 2023-02-20. Inference took 21 days on a server with 64 cores (2× AMD EPYC 7502) and 512 GB of RAM (~5 CPU years; Figures S1 S2). After post-processing, the ARG contains 2,689,054 nodes, 2,748,838 edges, and 2,285,344 mutations at 29,893 sites, and is stored in a 32 MiB file. Of the nodes, 855 represent recombination events, resulting in 316 distinct trees along the genome (some events occurred at the same breakpoints). Using the tskit Python API, the ARG can be loaded into memory in half a second, and the vectorised representation integrates tightly with powerful data science tools, such as NumPy^85^. For example, a tabular summary for the 2.75 million nodes, including sample IDs and Pango assignments (stored as metadata in the ARG), can be derived as a Pandas data frame^87^ in less than half a second. The ARG can therefore be easily accessed using familiar tools with minimal hardware requirements, enabling diverse bespoke analyses.

The ARG is a comprehensive and deeply validated record of the pandemic phase of SARS-CoV-2 evolutionary history, accurately reflecting a range of results from the literature. First, we compared the sc2ts ARG with the state-of-the-art UShER tree inferred from the same dataset by pruning both to their intersection of 2,475,418 samples and 27,507 sites (STAR Methods). Eliminating recombination nodes and their descendants (which we expected to differ), we found very similar relationships among lineages in the ARG and UShER tree based on tanglegrams for a set of representative samples from 814 well-sampled lineages (STAR Methods; Figures S3, S4, S5, S6). Overall, we also found close similarity between the ARG and UShER tree in terms of parsimony (Document S1.1) and imputation of missing and ambiguous bases (Document S1.2). We compared the mutational spectra of major Variants of Concern (VOCs) in the ARG against the UShER tree and a previous study^78^ (STAR Methods), finding close agreement (Figure S7). We also evaluated how well the ARG reflects the structure of the Pango nomenclature system by computing Pango assignments for all 2.75 million nodes in the ARG using Pangolin^92^ (STAR Methods). Only a small minority of the exported sample alignments (21,680; 0.87%) disagreed with the source consensus sequences in terms of Pango lineage assignment (comparable to the 0.27% disagreement between pangolin-data versions 1.21 and 1.29 in the source Viridian metadata). A majority of the Pango lineages (1473 of 2058) have monophyletic origins in the ARG, increasing to 1779 (86%) when we allow for multiple sibling origination nodes (Document S1.3). The ARG is time-resolved, with internal node dates estimated by a non-parametric method^93^ (STAR Methods). We show that the dates agree well with Nextstrain estimates (Figure S8) and overall accuracy is comparable to Chronumental^94^ applied to an UShER tree (Figure S9, Document S1.4). Finally, we verified that the origins of the Alpha (Figure S10, Document S1.5.1), Delta (Figure S11, Document S1.5.2) and Omicron (Figure S12, Document S1.5.3) VOCs reflect expectations from the literature^95–101^.

Deletions are an important part of SARS-CoV-2 evolution^102^ but are not incorporated into UShER trees and must be accounted for separately. Because deletions are difficult to distinguish from bioinformatic errors and their multi-base nature presents challenges to the LS HMM, we masked out all gap characters as missing data during primary inference. To approximate the effects of the most important deletions into the ARG, we remapped data using post hoc parsimony at 75 sites involved in deletions with a frequency of ≥ 1% reported by Li et al.^79^ during post-processing (STAR Methods). Although sites are treated independently in this approach, runs of mutations to the gap character at adjacent sites correctly identified many known high-frequency (Table S1) and recurrent (Table S2) deletions. For example, the three defining deletions of Alpha^95^ (11288:9, 21765:6, 21991:3) are correctly mapped above the Alpha origin in the ARG (Figure S10). The ARG captures known recurrent deletions in the N-terminal domain of the S1 unit of Spike (which is a deletion hotspot^103^), for example, the 21765:6 deletion (ΔH69/V70), which causes S-Gene Target Failure^104^ and is associated with increased efficiency in cell entry^105^, and the 21991:3 deletion which is associated with antibody escape^103^. See Document S1.6 for further analysis.

### Epidemiologically relevant recombinant lineages

Part of the success of the Pango nomenclature system^77^ can be attributed to the clarity of the rules under which new lineages are designated and their explicit phylogenetic basis. The first recombinant lineage XA was designated in 2021 based on the analysis of Jackson et al.^29^. Although the process of incorporating recombinant lineages in the Pango nomenclature system is well defined, the identification of such events is much more subjective, requiring the synthesis of multiple lines of evidence and often the manual inspection of phylogenies and sequence alignments by volunteers^106^. As of pango-designation version v1.33.1 (2025-03-31), a total of 146 distinct Pango X lineages have been designated.

**Figure 2:**
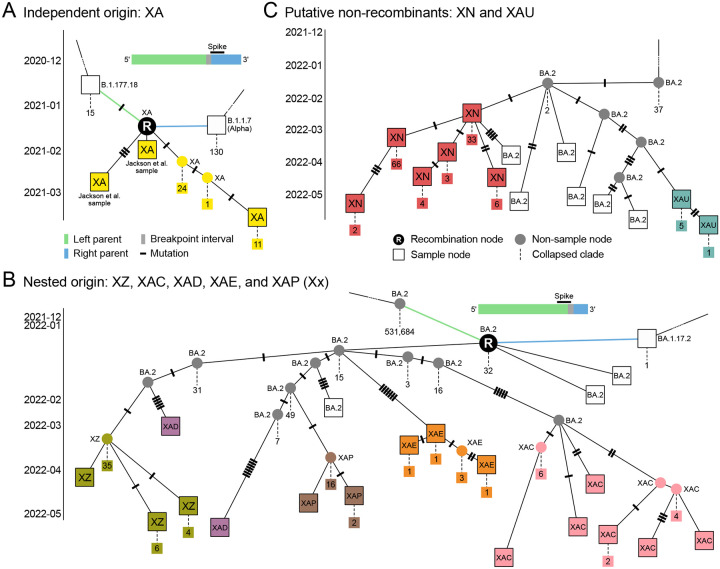
Illustrative subgraphs in the ARG. Boxes identify specific samples, coloured by their Pango X classification; smaller squares list extra samples descending from a node, hidden for brevity, with all hidden samples taking the same Pango designation as their parent. For the two recombination nodes, a genome bar above shows the location of the inferred breakpoint interval relative to the Spike gene. For simplicity, ancestral lineages above the recombinant parents or the MRCA are omitted. (A) Subgraph around the recombination node associated with XA. Two “Group A” recombinant samples reported by Jackson et al.^29^ are shown. (B) Subgraph around the recombination node associated with a group of “nested” Pango X lineages: we use the label Xx as a shorthand for this related group of recombinants; and (C) Subgraph around the MRCA of the closely related non-recombinants XN and XAU. For more detailed visualisations of these subgraphs, see Document S2.

By automatically detecting and incorporating recombination events into the ARG, sc2ts provides us with a systematic means of discovering and assessing evidence around these events. Table 1 summarises these results for the 44 Pango X lineages present in the ARG (see Document S1.7). For 16 Pango X lineages, the oldest node that has been assigned to the focal Pango lineage is a recombination node and that node is the ancestor of all samples assigned to that (or a descendant) Pango lineage. We consider these to be fully concordant (Type I) events, where the ARG reflects the hierarchical structure of Pango designation. Among these events, concordance with the community-consensus parent lineages and breakpoint intervals is excellent, with similar performance to the RecombinHunt^40^ and CovRecomb^41^ detection methods (STAR Methods; Table S3; see also Document S1.8 for analysis of the Jackson et al. recombinants). Of the Type I events, 13 are monophyletic for the focal Pango lineage (i.e., the descendants are entirely from that lineage); Figure 2A shows XA as an example.

There are five partially concordant events (Type II) in Table 1, which are recombination events closely associated with Pango X lineages that do not meet the strict origination criteria for Type I. Type III events are Pango origin nodes in the ARG that are derived from Type I or Type II recombination events. There is significant nesting among the Pango X lineage origin nodes within the ARG, with two major clusters of particular note. Although XQ is directly associated with a recombination event in the ARG, XAA, XAG, XAM, XR and XU are all closely derived, suggesting that they may be more parsimoniously explained as sublineages of XQ than de novo recombinant lineages (Document S1.9.12). The second cluster is based around a putative undocumented recombinant that is the ancestor of lineages XZ, XAC, XAD, XAE, and XAP, which we refer to as “Xx” (Figure 2B, Document S1.9.16).

The final class of events (Type IV) in Table 1 corresponds to nodes that are not associated with a recombination event in the ARG. These vary in their plausibility of being non-recombinant. For example, XN (Figure 2C) is only one mutation distant from a BA.2 sample and has little additional evidence to suggest recombination (Document S1.9.10). XB, on the other hand, has been thoroughly characterised^32^ and is likely misclassified by sc2ts because the required parent lineages were not present (Document S1.9.2).

The remaining 10 Pango X lineages (97 samples) present in the dataset are not included in the ARG because the HMM cost exceeds the inclusion threshold and there was insufficient support for retrospective inclusion (STAR Methods). These include “complex recombinants” such as XBC and XAY^35^ which are confirmed by sc2ts to have multiple breakpoints; see Document S1.10 and Table S6 for details. See Document S1.9 for extended analyses on all the Pango X lineages in the ARG; Figure S13 for copying patterns associated with Table 1; and Document S2 for ARG visualisations of the Pango X lineages.

### Quality control and validation of recombination events

Sequencing errors in genome assemblies^107^, incorrect read alignments^76^, and multi-nucleotide substitutions^108^ can cause short regions of a sample genome to match more closely to an alternative genome rather than the true parent. Such genomic regions can be mistaken for evidence of recombination. We use a simple quality control (QC) measure that counts the number of “loci” (clusters of closely spaced sites) supporting the left and right parents of a recombinant (STAR Methods), and effectively identifies a range of issues. Figure S16 shows the net number of supporting loci for the left and right parents for all 855 recombination events. The 501 recombinants having fewer than 4 net loci supporting each parent are highly enriched for samples sequenced using the AmpliSeq V1 protocol (70% of the recombinants despite making up 2% of the Viridian dataset), and 71 (14%) are associated with 6-base deletion in the Delta lineage, known to be affected by amplicon dropout^109^. The 354 remaining recombination events that pass this QC filter contain all of the recombinants associated with Pango X lineages (Table 1) and the Jackson et al. recombinants (Document S1.8).

We validated the recombination events in the sc2ts ARG by running 3SEQ^110^ on the putative recombinant sequence trios (STAR Methods). Of the 855 recombination events identified by sc2ts, 3SEQ verified 57% of them, rising to 95% verification among the 354 recombinants that passed our QC filter. In addition to 3SEQ, we ran the CovRecomb^41^ and rebar (https://github.com/phac-nml/rebar) recombination detection methods on the putative recombinant sequences, summarised in Table S4 (see also Figures S14, and S15). CovRecomb has a substantially lower validation rate at 25% among the QC-passing recombinants. This is expected, as CovRecomb is specifically intended to detect inter-lineage recombinants and has previously been shown to be less sensitive than other methods^41^. Rebar validation is intermediate, at 57% among QC-passing recombinants.

### Assessing evidence for recombination events

The LS HMM at the heart of sc2ts provides a simple and powerful means of quantifying the plausibility of a given recombination event. To do this, we force the HMM to find a Viterbi solution for the putative recombinant sequence with no recombination by specifying a large recombination penalty *k* (Figure 1). The difference between the numbers of mutations between the two solutions—the number of mutations “averted” by recombination—is a measure of the evidence in favour of recombination (similar to the “parsimony improvement” score in RIPPLES^38^). Table 1 shows the number of averted mutations for each of the 21 recombination events associated with Pango X lineages, with support ranging from very strong in the case of XC (the most parsimonious non-recombinant placement would require 37 additional mutations) to marginal for XG and XW, which have the minimal possible number of averted mutations.

**Figure 3:**
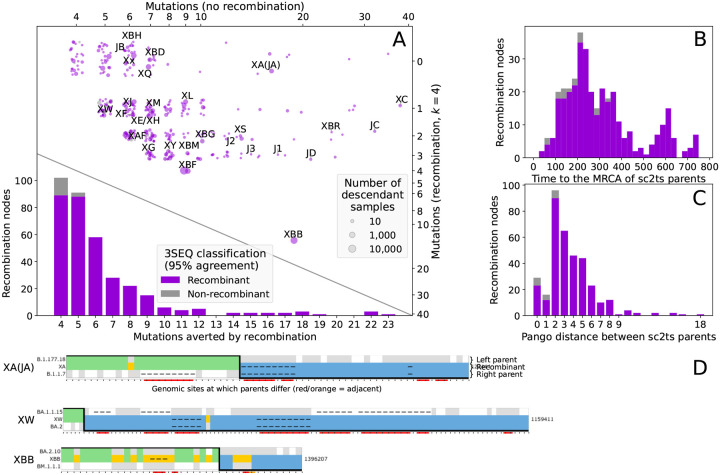
Properties of recombination events. A,B,C show summaries of the 354 sc2ts recombination nodes passing QC filters coloured by 3SEQ classification (recombinant: purple, non-recombinant: grey). (A) Upper: jittered scatterplot comparing number of mutations when recombination allowed (*y*: right axis) versus an alternative copying path disallowing recombination (*x*: top axis); recombination nodes in Table 1 are labelled starting with ‘X’ and those identified by Jackson et al.^29^ are prefixed with a ‘J’, e.g., Jackson group B (JB) and Jackson singleton recombinant 1 (J1). Point sizes reflect number of descendants. Lower: recombination nodes classified by number of mutations averted by allowing recombination (i.e., *x*−*y*). (B) Distribution of time to the MRCA of the parents of each QC-passing recombination node. (C) Distribution of the Pango lineage distance between parents. (D) Copying patterns (STAR Methods, Figure S17) for XA, XW and XBB.

Figure 3A plots the number of mutations required in the recombinant versus non-recombinant Viterbi solutions (upper scatter plot) and the resulting distribution of the number of averted mutations (lower histogram) over all 354 recombination events that pass QC. Also shown is the 3SEQ classification status of these recombinants. The 5% of recombinants that do not pass 3SEQ validation are among the most weakly supported, with 4 or 5 averted mutations. Figures 3B,C show the distribution of recombination events as a function of the divergence between their two parents. Figures S14 and S15 plot the same information as Figure 3 broken down by CovRecomb and rebar validation status.

Detectable recombination is rare in SARS-CoV-2, and ultimately judgments about plausibility require expert analysis. To aid such interpretation and help identify QC issues, we developed a visualisation tool inspired by SnpIt^111^ and the recombinant view of RIVET^106^, showing the recombination-informative sites between a recombinant and its parent sequences, and highlighting potentially problematic loci. Each copying pattern shows the positions where the allelic state in a recombinant (middle row) matches that of the left (upper row, coloured green) or right parent (lower row, coloured blue), or where the recombination event requires a de novo mutation (gold, with mutational change below). Figure 3D shows some example copying patterns, illustrating a spectrum of plausibility. XA is an example of a highly plausible recombinant, shown by the large number of supporting loci on the left and right. XBB is also strongly supported by many loci on the left and right, but the presence of many de novo mutations observed in the copying patterns suggests the existence of many genetically distinct, unsampled ancestors and some caution should be exercised when interpreting them (see Document S1.9.22 for more details). See Figures 3D and S13 for copying patterns of the Pango X lineages, and Figure S17 for copying patterns highlighting specific types of QC problems (and more details on the visualisations).

### Precision of recombination event times and positions

The genomic location of a recombination breakpoint in general cannot be determined exactly at single base resolution, but we can place bounds by inspecting sequences of the recombinant and its parents. The breakpoint interval of a recombinant is defined by two positions: a left position where the recombinant matches the left parent sequence but not the right, and a right position where the recombinant matches the right parent sequence but not the left. Genealogical methods such as sc2ts and UShER+RIPPLES use inferred parental sequences and are therefore in principle maximally precise. Other methods are limited in precision by the various proxies used to represent distinct parental lineages^37,39–41^.

**Figure 4:**
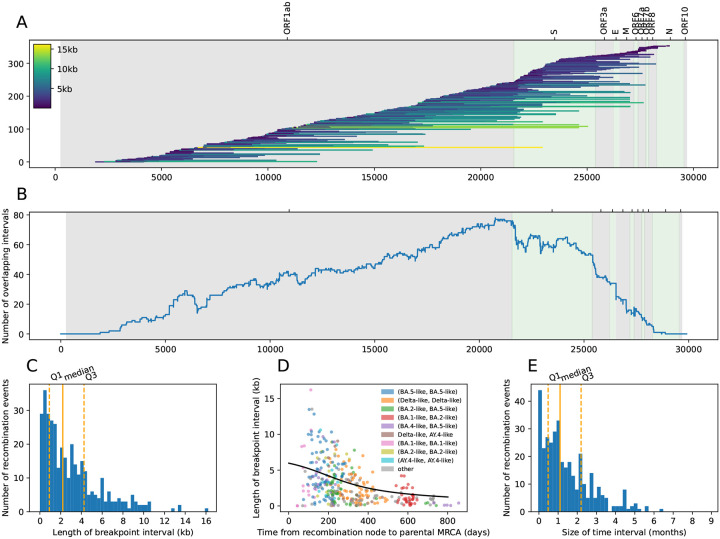
Recombination breakpoint and time intervals. (A) Distribution of the breakpoint intervals of 354 recombination events along the genome (annotated with gene labels on top). Each line represents a breakpoint interval coloured by its length. (B) Number of overlapping breakpoint intervals per site. (C) Length distribution of breakpoint intervals. (D) Relationship between breakpoint interval length and time between the recombination event and the parental MRCA. The black curve shows the theoretical expected relationship between these quantities, which is calculated assuming a genome-wide nucleotide substitution rate of ~0.06 per day (STAR Methods). (E) Length distribution of time intervals around recombination events.

The distribution of breakpoint intervals of the QC-passing recombination events in the sc2ts ARG shows an overall enrichment of breakpoints towards the 3’ end of the genome, particularly at the left boundary of the Spike gene (Figure 4A,B), consistent with the patterns seen in previous studies^38,41,106,112^. While this might result from a higher recombination rate in this region, it may also be due to the higher levels of polymorphism in the Spike gene, making recombination events more likely to be detected and allowing breakpoint intervals to be estimated more precisely. We found the median breakpoint interval length to be 2,207 bases (Figure 4C), indicating that the breakpoints of half of the events could be narrowed to a small region (less than ~7% of the genome), while the uncertainty around the breakpoints of the other events is considerably larger (up to ~54% of the genome). As expected from theory (STAR Methods), we found a negative relationship between the length of a breakpoint interval and divergence between recombinant parents (Figure 4D). Breakpoint intervals tend to be the smallest for events involving recombination between the deeply divergent Omicron BA.1 and BA.2 sublineages (the red points in Figure 4D).

The time interval of a recombination node in the ARG is bounded by the age of the youngest parent and the age of the oldest child of the recombination node. We found that for ~75% of the QC-passing recombination events, the time interval size is less than ~2 months (Figure 4D); for example, the time intervals of the events associated with the Jackson et al. samples span 5 to 48 days. Longer time intervals imply that a parental or a recombinant lineage might have been unsampled for an extended period of time.

### Emergence of recombinants over time

**Figure 5:**
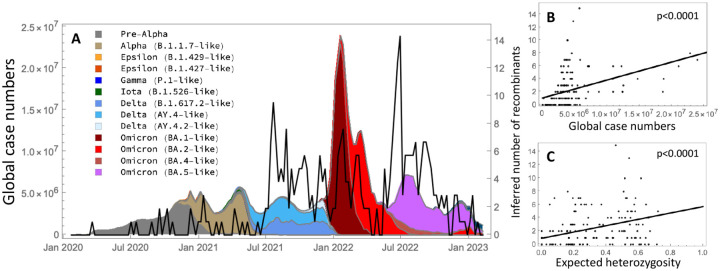
Recombination events across the pandemic. (A) Timing of the recombination events (black curve, right y-axis) relative to the global case numbers (^113^; left y-axis). Cases are colour-coded by their Scorpio labels, with the key showing those groupings with ≥ 1,000 samples in the Viridian dataset. Recombinants more commonly occur (B) in weeks with high case numbers and (C) in weeks with extensive lineage variation, as estimated by high expected heterozygosity; solid lines show fits by linear regression, allowing for non-zero intercepts to account for unknown cases and heterozygosity not captured by the Scorpio labels.

We examined the frequency of emergence of the recombinants over the course of the COVID-19 pandemic (Figure 5). We explored two hypotheses for the timing of recombination. First, we would expect recombination events to occur more often when case counts are high, which increases the chance of co-infection within an individual, allowing recombination to occur. Second, we would expect recombination events to be detected more often when lineage diversity is high, as this increases the chance that two sufficiently distinct viruses co-infected an individual, increasing our power to detect recombination.

We used the COVID-19 Data Repository at Johns Hopkins University^113^ to relate the timing of recombination events with global case numbers. We binned case numbers (*n*) by week from January, 2020 to March, 2023 and sub-divided by the proportion in each Scorpio label inferred within the ARG (shading in Figure 5). These lineages correspond loosely to a group of related lineages within a major VOC in the Viridian dataset. Alongside the case numbers, the black curve shows the number of recombinants inferred within each week along the ARG, focusing on the 354 events passing QC filters. Recombination events were inferred more often during the Delta and Omicron waves, when case counts were high. Overall, the number of recombination events rose with the global case count (Figure 5B; linear regression: 1.0 + 2.7 × 10^−7^*x*, *p* = 1.1 × 10^−7^). Recombination events also rose with the diversity of lineages in each week (Figure 5C; linear regression: 0.9 + 4.8*x*, *p* = 3.4 × 10^−7^), measured as the chance that two randomly drawn viruses have different Scorpio designations (*H*, referred to as the “expected heterozygosity”).

We then asked which single measure best explains the number of recombination events inferred each week, considering *n*, *H*, *nH*, *n*^2^, *n*^2^*H*. We included the squared number of cases each week thinking that it might better predict co-infections. The best single predictor of recombination events involved the product of cases and expected heterozygosity (*nH*), with an adjusted *R*^2^ of 23.7%. This measure of case diversity (*nH*) explained 49% more of the variation in recombination events per week than the next best predictor (*H*, adjusted *R*^2^ of 15.9%). While consistent with the hypotheses that recombination events occur more often when infection rates are common and are detected more often when viral diversity is high, we note several sources of uncertainty. Global case numbers were underreported, particularly during the Delta and Omicron waves^114^. Furthermore, there may be substantial uncertainty in the timing of a recombination event, particularly if these occur in chronically infected individuals (as reported by^115^) with substantial delays before onward cases are detected. Finally, the filters that we applied removed all but the most strongly supported recombination events, although similar conclusions were obtained when all 855 recombinants detected by sc2ts were considered (STAR Methods).

### Comparison with UShER+RIPPLES

The most comprehensive analysis on recombination in SARS-CoV-2 to date has been performed using the combination of UShER and RIPPLES^38^. RIPPLES works by taking an UShER phylogeny and systematically breaking up sequences subtended by long branches into multiple segments that are placed independently. A recombination event at a node is flagged if the overall parsimony of these multiple placements sufficiently improves over the original single-parent origin in the phylogeny. This approach has much in common with the LS HMM used in sc2ts, but with one important distinction. While sc2ts detects and incorporates recombination in “real-time” as sequences are added to the ARG in daily batches, RIPPLES operates on a complete phylogeny, attempting to detect recombination post-hoc among millions of nodes. Like any phylogenetic reconstruction method that ignores recombination, UShER must force placement of recombinant sequences in places that minimise the discrepancy across the entire sequence, which may not be near any of the true parents. These permanent misplacements distort the phylogeny^3^, and subsequent tree reorganisations to maximise overall parsimony^25^ may break up the artefactually long branches used to detect recombination.

We compared the performance of sc2ts to detect recombination against RIPPLES run on the Viridian UShER phylogeny, subset to the 2,475,418 samples shared with the ARG (STAR Methods). To enable exact comparison, we omitted the QC filtering steps from both methods, and initially set the RIPPLES “parsimony improvement” parameter to *p* = 4 (rather than the more lenient default of 3), to match the equivalent *k* = 4 setting used in sc2ts (STAR Methods). Under this setting, RIPPLES reported 1174 recombination events of which 601 were validated by 3SEQ (51%; STAR Methods). We then compared the specific events reported by both methods by aligning them using sets of descendant samples (STAR Methods), giving 468 events shared between RIPPLES and sc2ts (Figure S18). The 706 recombination events detected by RIPPLES that do not correspond to a recombination event in the sc2ts ARG can largely be explained by a combination of the strict time ordering imposed by sc2ts and the differing sets of problematic sites masked by sc2ts and UShER (Document S1.11). The 387 events detected by sc2ts and not by UShER+RIPPLES include some of the well characterised Pango X lineages. Table S8 shows the events detected by UShER+RIPPLES associated with samples assigned to Pango X lineages, showing that at *p* = 4 only 9 of the Pango X lineages are present and with unequivocal recombinant origin for 6 (contrast with 16 Type I events in Table 1). We therefore reran RIPPLES with *p* = 3, resulting in 4111 events overall and increasing the intersection of events with sc2ts to 595 (Figure S18). At this more lenient threshold, 23 of the 44 Pango X lineages are identified as descendants of RIPPLES recombinant events, with unequivocal origins for 10 lineages, and 7 lineages associated with two competing events (Document S1.11). Thus, a more lenient threshold (*p* = 3) was needed for UShER+RIPPLES to detect a similar fraction of Pango X lineages as sct2ts with *k* = 4, resulting in more false positives. That said, XB and XAJ are among the lineages detected by UShER+RIPPLES, even at *p* = 4, strengthening evidence that these are false negatives for sc2ts (Table 1).

The choice of the *p* threshold has a large effect on the number of recombination events detected by RIPPLES, and previous studies have used the default value of 3^38,106^. For example, when applied to an UShER phylogeny of ~1.6 million genomes sampled up to May, 2021, RIPPLES reported 589 unique recombination events with 43,104 descendant samples—corresponding to ~2.7% of all samples of recombinant origin. Over the same time interval, the sc2ts ARG (*k* = 4) identifies 14 recombination events passing QC with 172 descendant samples among 501,235 samples (0.034%), a 40- to 80-fold reduction. The sc2ts ARG identifies more of the well-characterised Pango X lineages at a more stringent threshold, and therefore provides us with a conservative lower bound on the frequency of detectable recombination events in SARS-CoV-2.

## DISCUSSION

There are two primary contributions of this work. The first is an ARG for 2.48 million SARS-CoV-2 genomes. Through multiple layers of validation against results from the literature, we have demonstrated that this ARG synthesises the evolutionary features of genetic inheritance, point mutations, deletions and recombination in a single cohesive structure. The ARG is freely available and easily accessible using the tskit software ecosystem, a rich and efficient platform for recombination-aware analyses widely used in population genomics. This combination of a deeply validated pandemic-scale ARG with a mature and highly capable software platform forms a unique resource. Previous landmark ARGs^54,116^ have helped spur the development of ARG-aware methods in human genomics^117–120^. We hope that the ARG provided here will similarly enable a range of recombination-aware analyses of SARS-CoV-2 and catalyse a wave of methodological developments integrating recombination into pathogen genomics. For instance, annotating the ARG with experimentally derived fitness estimates for mutations can help elucidate selection pressures on different recombinants; saltational variants can also be detected by analysing the rate and position of mutations along long branches (which persist after accounting for recombination). We envisage the ARG as a long-term stable resource—building on the high-quality, freely available reassemblies of the Viridian dataset^76^—to facilitate the study of the pandemic phase of SARS-CoV-2 in an open and reproducible manner.

The other primary contribution of this work is the method used to infer the ARG, sc2ts. The ability to infer an ARG over millions of whole genomes is a step change in scale over existing ARG inference methods for pathogen data^15–17,19,33^. To ensure these innovations are well-founded, we have paid significant attention to methodological detail, carefully validating the ARG (and consequently the method) against known results. Sc2ts is currently specialised to take advantage of unique features of the SARS-CoV-2 dataset, but the methods may be readily adapted to other pathogens. The LS HMM is the most important element of sc2ts, efficiently finding parsimonious placements for samples among all possible recombinant solutions. The current parametrisation—emphasising interpretability—is simplistic and could readily be extended to account for mutation rate variation along the genome^121^ or transition-to-transversion rate bias^78,122^. Our analysis of deletions shows that valuable phylogenetic signal is lost when they are ignored, and an important avenue for future work is to explore ways of incorporating them directly into the HMM. Another methodological innovation is the use of the tsdate method^54,93^ to estimate internal node dates in the ARG, illustrating the reuse of methods initially developed for population genetic applications. While results are encouraging, dating of phylogenies is a complex task^123^, and there is certainly room for improvement.

Further contributions of this work arise from the detailed study of the sc2ts ARG, providing insights into the evolution of SARS-CoV-2 and the COVID-19 pandemic. First, we clarify the relationships among recombinant Pango X lineages, finding examples of clustering of named lineages under a single shared recombination event. Second, we used the ARG to study breakpoint intervals for recombination events, showing that the relationship between the size of the interval in base pairs and the divergence time of the parental lineages matches theoretical expectations. Thirdly, we demonstrate a clear relationship between genetic diversity and the rate of detectable recombination, with the best predictor of the number of recombination events over time being the product of global case numbers and lineage diversity. Finally, and perhaps most fundamentally, through our conservative parameter choices and real-time handling of recombination events, we provide a stringent lower bound on the amount of recombination that occurred during the pandemic phase of SARS-CoV-2. These results underscore the utility of the ARG and provide a template for future analyses.

### Limitations of the study

We have focused on applying sc2ts to the Viridian dataset^76^ rather than the larger GISAID resource^6^ for two reasons. Firstly, the Viridian dataset is of higher quality and major efforts have been made to remove sources of error. Applying sc2ts to GISAID data is straightforward, but significant additional efforts would be required to address data quality issues. Secondly, the Viridian data is publicly available and redistributable, enabling us to share the resulting ARG without restrictions.

It is important to acknowledge that sampling is non-uniform both geographically and over time^124,125^. Sc2ts performs best in the data-dense regime, when the underlying assumption that genetic diversity is gradually accumulating within the structure holds. Large changes (i.e, saltation events) present significant challenges, and the results of the algorithm should be regarded more critically in these situations. Future methodological improvements may combine the strengths of sophisticated model-based inferences with the power of non-parametric inference^126^ when such data sparsity is detected.

## RESOURCE AVAILABILITY

### Data and code availability

- The ARG in tskit format has been deposited at Zenodo and is publicly available at https://zenodo.org/records/17558489. The UShER tree in tskit format is also available, as well as the intersection files.
- The sequences alignments in VCF Zarr format have been deposited at Zenodo and are publicly available at https://zenodo.org/records/16314739.
- The Snakemake workflow for aligning and converting the Viridian v05 dataset to VCF Zarr is publicly available at https://github.com/jeromekelleher/sc2ts-paper/blob/main/viridian_dataset/.
- The complete configuration file for running sc2ts to build the ARG above is publicly available at https://github.com/jeromekelleher/sc2ts-paper/blob/main/inference/viridian_config.toml.
- The Snakemake workflow for ARG post-processing is publicly available at https://github.com/jeromekelleher/sc2ts-paper/blob/main/arg_postprocessing/Snakefile.
- The original code of sc2ts is publicly available on GitHub at https://github.com/jeromekelleher/sc2ts/.
- The Jupyter notebooks and the Mathematica script to perform the analyses in this paper are publicly available on GitHub at https://github.com/jeromekelleher/sc2ts-paper/.

## STAR METHODS

### Method details

#### Ancestral Recombination Graphs

The topology of an Ancestral Recombination Graph (ARG) can be defined as a set of *nodes*, representing a genome that existed at some specific time, and *edges* that describe the genetic inheritance relationships between those genomes^43^. Each edge defines the parent and child nodes and a genomic interval over which inheritance occurs. The “succinct tree sequence” ARG encoding defines these relationships in a simple and concise tabular format, sufficient to represent arbitrarily complex patterns of recombination^43^ and providing the basis for a range of highly efficient algorithms^47,62,118,119,127^. The mature and feature-rich tskit library has interfaces in Python, C and Rust, extensive documentation, and is used in numerous downstream applications^49,53,54,63–68,70–75^.

Similarly to the Mutation Annotated Tree (MAT)^26^ format (used by UShER^24^, matOptimize^25^, RIPPLES^38^, and RIVET^106^), tskit incorporates mutational information directly in the data model. This is done using the site (defining the genome position and ancestral state) and mutation (defining the site, node, and derived state) tables. In addition, tskit provides a flexible system for attaching metadata to all aspects of the data model, which can either be in JSON format for flexibility (used heavily by sc2ts for debugging information) or in a vectorisable packed binary format for efficiency (used to attach sample identifiers and Pango lineage assignments in the final ARGs). Tskit also provides a “provenance” table, which provides a mechanism for recording software version and parameter information through each step of complex processing pipelines.

#### Pandemic-scale alignment storage

To provide the efficient access to alignment data required for inference with sc2ts, we developed a new format based on the VCF Zarr^80^ specification. Briefly, Zarr is a storage format for scientific data, originally developed for the *Anopheles gambiae* 1000 Genomes Project^128^, and is now seeing widespread adoption across the sciences^129–136^. Zarr is essentially a simple mechanism for storing n-dimensional array data as a regular grid of compressed chunks, which provides high levels of data compression, efficient access across multiple dimensions, and is ideally suited to modern cloud-based deployments^80^.

We converted the Viridian v05 dataset^76^, consisting of 4,484,157 SARS-CoV-2 consensus sequences (125 GiB over 48 FASTA files) and 30 metadata fields (1.4 GiB TSV file) to VCF Zarr using a reproducible Snakemake^137^ pipeline. We aligned each sequence to the Wuhan-Hu-1 reference sequence (MN908947.3) using MAFFT v7.475^138^, with the option --keeplength to retain the reference coordinate system. We then used sc2ts import-alignments and sc2ts import-metadata to convert all alignments and metadata to VCF Zarr format, resulting in a single 401 MiB file (around 77× smaller than gzip compressed FASTA^80^).

A classical trade-off in bioinformatics is whether to store data in sample-major (FASTA) or variant-major (VCF) format, determining whether the data can be accessed efficiently either by row or column of the variant matrix. Because VCF Zarr is chunked in two dimensions, we can now access either sample alignments or the variation data for a given site across all samples efficiently. While the VCF Zarr encoding of the MAFFT-aligned Viridian sequences and metadata can be accessed via native Zarr libraries in several popular programming languages (see Czech et al.^80^ for an overview), the sc2ts Python library provides a convenient interface. The sc2ts.Dataset class provides efficient access to alignments for samples and variant data for sites along the genome, as well as methods to export subsets of the data to FASTA if required. Document S1.2 gives an example of using this interface, where we retrieve alignment data for millions of samples in a few minutes on a standard desktop computer with minimal memory overhead. See Czech et al.^80^ for more details and benchmarks.

Both the pipeline and converted Viridian dataset are available for download; see Data and code availability for details.

#### Sample filtering and alignment pre-processing

The collection date of a sample is an important piece of information for sc2ts. We used the Viridian consensus date (“Date_tree“), based on information from COG-UK, GISAID, and ENA/SRA as our source. Samples that have missing or incomplete dates (e.g., 2020-01) were omitted. Samples with a collection date of December 31, 2020 were also omitted, as the number of sample on this date (~40,000) was a major outlier for this period of the pandemic and is likely enriched for incorrect collection dates. Manual checking (by cross-referencing the ENA and COG-UK Mutation Explorer) confirmed that some of these samples were not collected in 2020.

A set of problematic sites for SARS-CoV-2 that are enriched for systematic sequencing errors or high levels of homoplasy was compiled and updated during the pandemic^108,139^. Although these sites are recommended to be masked for UShER inference^107^, the list has not been updated to take into account the systematic improvements made by the Viridian dataset, and we therefore assembled a custom set of sites to mask. To do so, we first built a preliminary ARG from the samples collected up to June 30, 2021, without any site masking. Then, we identified the top 100 sites in terms of mutation counts in the ARG. Only 11 of these sites (635, 8835, 11074, 11083, 15521, 16887, 21304, 21305, 21575, 21987 and 28253) were present in the problematic sites file (https://github.com/W-L/ProblematicSites_SARS-CoV2), and most sites in this list were typical in terms of the number of mutations. We therefore masked our 100 most mutation-rich sites to remove sequencing errors and improve matching performance in the LS HMM.

Alignments read from the VCF Zarr file were first preprocessed to mask any non-nucleotide characters (A, C, G, or T) as missing data. Then, excluding the 100 sites identified as problematic, we filtered any samples that had missing data at more than 500 sites (this was set to 10,000 sites for the initial period up to 2020-03-01 when sampling density was low).

#### Li and Stephens model

The Li and Stephens (LS) model^81^ is an approximation of the coalescent with recombination that captures many of the key features of the joint processes of mutation and recombination. It is a Hidden Markov Model (HMM) in which a focal genome is modelled as a sequence of nucleotides that are probabilistically emitted as an imperfect mosaic of a set of reference genomes (Figure S19). The LS model is an HMM that models a focal genome as a sequence of nucleotides (the observed states), which are probabilistically emitted as an imperfect mosaic of a set of genomes in a reference panel (the hidden states). The process of switching between the genomes in the reference panel is governed by a transition matrix, and mismatches between a focal genome and a genome of the reference panel (from which it is “copied”) are permitted as emissions.

In the standard formulations of the LS model^81,84^ the probability of switching between the hidden states (i.e., genomes in a reference panel) when going from one site to the next is dependent on (1) the rate of recombination between the sites and (2) the number of genomes in the reference panel (*n*). Sc2ts uses the same formulation except that *n* is set to 1. This special case of the LS model leads to an intuitive interpretation of the ratio between the probability of switching and the probability of mismatch: When considering only two copying paths, the path having *k* mismatches and the other path having one switch but no mismatch are equally probable. We call this parameter *k* the “recombination penalty”. This parameter thus allows us to adjust the relative importance of recombination and mutation when finding the best explanation for a given sample. For simplicity, we assume that any single base mutation causing a mismatch is equally probable, ignoring transition-transversion biases and variation of mutation rate along the genome.

We use the efficient Viterbi algorithm (which finds the most likely sequence of hidden states in a HMM) implemented in tsinfer^53^ to find copying paths under this model. We updated this implementation to include a “likelihood threshold”, which allows all possible paths through the HMM with likelihood less than a given value to be considered equally improbable. This approach leads to major gains in likelihood compression, saving memory and reducing computation time. We then use this likelihood thresholding approach to iteratively process the samples for a given daily batch, first finding all exact matches, then all with 1 mismatch, etc. By setting the appropriate likelihood threshold for the HMM, we are guaranteed that any HMM solutions found at this threshold are correct, and can then run the HMM again with a lower threshold for the remaining samples. Close matches are found very quickly using this approach, and it is only samples that are more distant from the current ARG that require significant processing time and memory. This is reflected in the computational resources reported in Figures S1,S2.

#### Choice of k parameter

We explored using several values of *k*, by running sc2ts with *k* set to 3, 4, and 5 on the samples collected in 2020. We would expect that a good value for *k* should lead to parsimonious ARGs that capture plausible recombination events without incurring too many mutations, particularly reversions. A value of *k* too low could result in many artefactual recombination events, whereas a value of *k* too high could miss many genuine recombination events. We found that ARGs built with *k* set to 3 contained many dubious recombination events, but ARGs built with *k* set to 5 failed to include the well supported recombination events documented in a previous study^29^. Hence, we decided to use *k* set to 4 to infer the final ARG described in this study.

#### Tree inference from daily sample clusters

With tens of thousands of samples being added to the ARG per day, there are often clusters of hundreds of sequences with the same LS Viterbi solution. That is, large clusters of samples attach to the same node (or more generally, the same recombinant path) in the ARG. While some of these samples will require no extra mutations (as they are identical to the attachment node), in general there will be complex patterns of shared mutations among the samples reflecting their evolutionary relationships. We use a standard neighbour-joining algorithm^140^ to infer these within-cluster evolutionary relationships. We first compute the Hamming distance between all pairs of samples using SciPy^86^, and then use biotite^90,91^ to compute the neighbour-joining tree. Then, mutations are mapped back onto this daily sample cluster tree using the Fitch-Hartigan parsimony algorithm^141,142^ implemented in tskit. Finally, the resulting tree and mutations are added to the ARG at the attachment node(s) specified by the shared LS copying path.

#### Parsimony improving heuristics

Attaching trees built from the clusters of samples that copy from a particular node is an inherently greedy strategy and can produce inferences that are clearly unparsimonious. The final step in adding a daily batch of samples to the ARG is therefore to perform some local updates that target specific types of parsimony violations in the just-updated regions of the ARG. There are currently two parsimony-increasing operations applied, which we refer to as “mutation collapsing” and “reversion pushing” (Figure S20).

Given a newly attached node, mutation collapsing inspects its siblings from previous sample days to check if any of them share (a subset of) the mutations that it carries. If so, we increase the overall parsimony of the inference by creating a new node representing the ancestor that carried those shared mutations and make that new node the parent of the siblings carrying those shared mutations. The patterns of shared mutations between siblings can be complex, and the current implementation uses a simple greedy strategy for choosing the particular mutations to collapse. 119,844 nodes were added to the ARG during primary inference by mutation collapsing.

The reversion push operation inspects a newly added node to see if any of its mutations are “immediate reversions”; that is, are reversions of a mutation that occurred on the new node’s immediate parent. We increase the overall parsimony of the inference by “pushing in” a new node which descends from the original parent and carries all its mutations except those causing the reversions on the newly added node. Reversion pushing led to the addition of 52,336 nodes during primary inference.

#### Filtering time travellers

Time-travelling samples—those in which the recorded collection date differs substantially from their true collection date—pose significant problems for sc2ts and are present at a significant frequency. For example, of the 43 Scorpio^143^ designations present in the Viridian dataset studied, 32 have samples present with sampling dates in 2020, including lineages that do not arise until late 2022. Inserting these time-travellers into the ARG at the claimed sampling date causes major artefacts, and they must therefore be filtered out. We use the simple approach of filtering out samples that exceed a given “HMM cost”, i.e., requiring too many mutations or recombination breakpoints to plausibly represent the steady accumulation of diversity expected with dense sampling. The HMM cost for the Viterbi solution for a sample under the LS model is *k* times the number of recombination events plus the number of mismatches, where *k* is the recombination penalty (here, *k* = 4). After some experimentation, we set the HMM cost threshold to 7, disallowing samples that require 8 or more mutations (or, e.g., 1 recombination and 4 mutations, or 2 recombinations) from immediate inclusion (see next section) in the ARG. This approach has the benefit of filtering samples enriched for errors as well as time-travellers.

#### Inserting saltational lineages

The HMM cost threshold approach outlined in the previous section is effective at removing time-travelling samples, but it also filters out important evolutionary events such as “saltations” that characterize major VOCs. These lineages are characterised by a high number of accumulated mutations, and are hypothesised to emerge from chronic infections during which the virus is exposed to individual-specific immune selective pressures^144,145^. To distinguish time travellers from true saltational lineages, we re-evaluate the evidence for each lineage with a high HMM cost as additional samples are added. If these samples form a sufficiently plausible group over successive days (under criteria described in the next paragraph) sc2ts adds the group to the ARG as a “retrospective sample group” using same mechanisms as standard daily sample clusters.

Distinguishing a truly emerging outbreak from different forms of correlated error is non-trivial, and sc2ts has 5 parameters to fine-tune the behaviour. Firstly, we require at least 10 samples in the group to ensure there is sufficient support over a time window of seven days. Then, we build a local tree of the samples and consider some tree metrics to evaluate whether the samples within the group are closely related. If we have at least 2 mutations shared by all samples, there are at most 2 recurrent mutations within the group and at most 5 mutations unique to each sample, the retrospective group is accepted for inclusion. Although this process is highly heuristic, it successfully captured the majority of saltational lineages such as Alpha (Figure S10), and the vast majority of samples were added following the standard daily sample grouping mechanisms. In total, 882,398 daily sample groups were added to the ARG, while only 100 were added via this retrospective grouping mechanism.

The Delta, BA.1, and BA.2 lineages were particularly challenging, however, as the initial emergences of these lineages were not densely sampled. In this case, there is little information for the retrospective sampling grouping mechanism to work with and it is very difficult to distinguish short-term time travellers from true outbreak samples. For these cases, we chose specific high-quality samples with confident dates as “seeds“ which are unconditionally included in the ARG on the specified date.

For Delta (first detected in December, 2020 in India^146^), we used two seeds: ERR5461562 assigned to B.1.617.1/Kappa (dated February 22, 2021 from the UK) and ERR5676810 assigned to B.1.617.2/Delta (dated March 23, 2021 from the UK). Kappa is closely related to Delta^99^, and therefore seeding it helps to reconstruct the origin of Delta. These are the earliest Kappa and Delta samples in the Viridian v04 dataset that were used for the designation of these Pango lineages. The resulting subgraph around Delta in the ARG can be seen in Figure S11; for further explanation, see Document S1.5.2.

For Omicron BA.1 and BA.2 (first detected in South Africa or Botswana in November, 2021^146^), we used SRR17041376 and SRR17461792, respectively. We chose SRR17041376 (dated November 6, 2021 from South Africa) for the BA.1 seed, as it is among the earliest African Viridian samples, and its initial placement in the ARG mirrors the initial placement of a subsequent high-quality COG-UK sample (ERR7443564). For BA.2, we chose SRR17461792 (dated November 27, 2021 from South Africa), which is the earliest BA.2 sample present in the Viridian v04 dataset. Note that this sample is dated later than SRR17041376 (the BA.1 seed), allowing time for the BA.1 lineage to become established and accumulate descendant samples in the ARG. The resulting subgraph around these Omicron lineages in the ARG can be seen in Figure S12. For further explanation, see Document S1.5.3.

With the combination of these mechanisms—gradual accumulation of diversity through daily sample groups, retrospective group inclusion and manual seeding—we successfully incorporated all major lineages in the dataset into the ARG (Figure S2).

#### Primary ARG inference

The “base ARG” we infer with sc2ts is the starting point for later analyses, and this step dominates overall processing time. To summarise, we first initialise the ARG using the Wuhan-Hu-1 reference sample (MN908947.3) as the root node, and then ran the algorithm day-by-day using the parameters described in the preceding sections and documented in the configuration file (see Data and code availability). As discussed above, computational resources are dominated by the LS model (Figures S2,S1) with all other aspects contributing negligibly.

#### Recombinant rematching

The first step in ARG postprocessing is to examine putative recombinants to determine whether a more parsimonious explanation is possible. This is done by rematching the inferred recombinant sequence against the ARG pruned back to include only samples before the date of the recombinant. Where the recent ancestry of a recombinant includes a branch containing large numbers of mutations, it is possible that a non-recombinant solution could be found by postulating an intermediate node along that branch, that contains only a subset of those mutations. In particular, for a given mutation-rich branch, artefactual recombinants may be found in which one recombinant region matches to a node like the parent whereas another region matches to a node like the child of that branch. Such recombinants can be identified by the presence of recurrent or reversion mutations in the left or right parent branches. To help remove these recombination events from the ARG, we note the genomic positions of mutations associated with the recombinant, and identify branches in their ancestry which have mutations at the same positions. Of these branches, we chose the one that contains the largest total number of mutations as a candidate for intermediate node creation. This is done by sorting the mutations on the branch such that the ones that match the allelic state in the recombinant are placed above the intermediate node, and the others are left below the node. We refer to this process as “long branch splitting”, and it creates additional nodes which are available for recombinant rematching.

Using recombinant-rematching, 58 recombinants were identified as being more parsimoniously explained by matching to existing nodes in the ARG (inserted by subsequent parsimony improving heuristics) and 16 by long branch splitting. Of these 16 recombinants, two are noteworthy as being the origins of the Delta (B.1.617.2) and Omicron-BA.2 lineages in the ARG. Both result in substantially fewer mutations (15 fewer for Delta, 6 for BA.2) than the previous recombinant HMM solution. We then updated the ARG to remove these 74 artefactual recombinants by “rewiring” the recombination node to the more parsimonious non-recombinant solution. This resulted in 16 additional nodes, 182 fewer mutations and reduced the number of trees along the genome from 348 to 317. The long branches involved in the origins of Delta and BA.2 originally joined B.1 to B.1.167 and B.1.1 to BA.1 respectively; they can be seen in Figure S11 and S12 with inserted intermediate nodes leading to the Delta and BA.2 origination nodes.

#### Postprocessing pipeline

This section described the various postprocessing steps that we performed after primary inference, which is encoded as a reproducible Snakemake workflow (see Data and code availability).

The first step is to perform recombinant rematching as described in the previous section, to find any recombinants that can be more parsimoniously explained either by matching to nodes subsequently added or by targeted splitting of long branches.

For performance reasons, samples that match exactly to an existing node in the ARG are not added during primary inference, but are stored in a secondary “match database”. During postprocessing, we added 1,252,208 of these exact match samples into the ARG using the stored information.

During primary inference, gap characters (“-”) are masked out as missing data, but this excludes both important information about deletions from the ARG as well as potentially informative phylogenetic signal. We therefore performed post-hoc parsimony mapping at 163 sites to include the effects of deletions and to ensure that all Pango-informative sites are included in the ARG. Using deletions with a minimum frequency of 1% (count of sequences divided by 9,149,680 sequences analyzed) from Table S1 of Li et al.^79^, we chose 75 sites for deletion-remapping (see Document S1.6 for analysis of the major deletions identified from this process). In addition, we remapped data at 88 sites that were excluded from primary inference and are defined as Pango lineage defining sites by Freyja^147^ (https://raw.githubusercontent.com/andersen-lab/Freyja/refs/heads/main/freyja/data/lineage_mutations.json). Alignment data for these 163 sites was then mapped to the ARG using the Fitch-Hartigan algorithm^141,142^ implemented in tskit, including the gap character as a state along with the four nucleotides. Following this, we iteratively applied the node parsimony heuristics in sc2ts until there were no further reductions in the numbers of mutations.

We computed Pango assignments for all 2,747,985 nodes in the ARG by first exporting their aligned sequences to a 77GiB FASTA file and then running pangolin (version 4.3.1; pangolin-data version 1.29). Note that this version of pangolin uses UShER placement for classification^148^. The resulting Pango and Scorpio designations were then stored as metadata associated with each node in the ARG.

When then estimated non-sample node dates using the “variational-gamma” algorithm in tsdate^54,93^ version 0.2.3. See Document S1.4 for more details and analysis of these dates.

Finally, we reduced the metadata stored in the ARG to a minimal set (node sample IDs, and the computed Pango and Scorpio designations), encoded to enable efficient columnar access, and compressed the file using tszip (version 0.2.5). The final file requires 32MiB of storage.

#### Quality control of recombination events

We examined the genomic positions at which the suggested parents differed in allelic state. Where such sites clustered closely (3 or fewer bases apart), we treated the entire cluster of sites as a single *locus*. For each genomic region identified by the HMM as having a single parent, we calculated the “net number of supporting loci” by counting the number of loci in the region that supported the suggested parent minus the number that did not support it. Finally, with all the recombination events in the ARG having a single breakpoint, we calculated the net number of supporting loci on the left and right sides of each breakpoint, and used this to plot all the 855 recombination events in Figure S16.

This simple QC measure effectively differentiates artefactual recombination events from those that are well supported. All the sc2ts-detected recombination events associated with the Pango X lineages (Table 1) occur in the upper right of Figure S16, with high numbers of net supporting loci on both the right and left parents. In contrast, the other regions of the plot, where one or both parents are less well supported, are dominated by recombination events associated with samples sequenced using the Ion AmpliSeq protocol. Coupled with closer inspection of copying patterns (see below), we chose a QC cutoff of 4 or more net supporting loci on both the left and right side of the breakpoint. This leads to 354 QC-passing recombination events (Figure S16, upper right quadrant), compared to 501 recombination events for which there is less support and which may be artefactual (shaded quadrants).

Inspection of the copying patterns for all the recombination events identified associations between recombination events that failed QC and known problematic loci in the SARS-CoV-2 genome. Figure S17 shows illustrative copying patterns (Q1 to Q4 correspond to the labelled quadrants in Figure S16). Q1 shows a well supported recombination event that passed QC (albeit marginally), having a net number of supporting loci of 4 on both sides of the breakpoint. Q2 shows an artefactual recombination event caused by a 6-base Delta lineage-defining deletion. There are 71 (14%) QC-failing recombination events involving this deletion, and many cases are affected by a known amplicon dropout caused by this particular deletion^109^. Q3 shows an artefactual recombination event caused by a combination of this Delta deletion and a known MNS (see below). Finally, Q4 shows an artefactual recombination event associated with the A507T-T508C-G509A MNS, which has been identified as a sequencing error due to incomplete read trimming^149^. There are 9 recombination events involving this particular MNS, of which 6 failed QC.

#### Validation of recombination events

We validated recombination events in the sc2ts ARG using 3SEQ^110^ as follows. We first exported the alignments for the recombination node and both parents to FASTA format, and then ran 3SEQ in “full mode” independently for each recombinant. Specifically, we ran 3seq -full parents.fa recombinant.fa using default thresholds. We used 3SEQ source code downloaded from https://gitlab.com/lamhm/3seq at git hash e45ee3b4.

For CovRecomb^39^, we obtained the original alignments for the “causal” samples for each recombination node, and ran CovRecomb-Local-Version with default settings. We used source code downloaded from https://github.com/wuaipinglab/CovRecomb at git hash 1fbf6b2.

We ran rebar in a similar manner, exporting the original alignments for the causal samples to FASTA and using default parameter values. We used rebar version 0.2.1 from bioconda^150^, and rebar dataset version “2025-01-28”.

These steps are included in the Snakemake postprocessing workflow (see Data and code availability).

#### Conversion of UShER tree to tskit

To facilitate comparison with the sc2ts ARG, we first converted the Viridian UShER tree from MAT^26^ to tskit format. We first downloaded the tree in JSON format (tree.all_viridian.202409.jsonl) and converted the tree topology to tskit node and edge table descriptions. We then downloaded the protobuf formatted tree (tree.all_viridian.202409.pb.gz), exported the mutations to nucleotide format using matUtils summary --translate (usher version 0.6.3 from bioconda), and translated these mutations into tskit site and mutation tables. The resulting file contains 5,345,019 nodes, 3,364,841 mutations and requires 29MB of storage space in tszip format (usher_viridian_v1.0.trees.tsz). We validated that the data encoding by comparing the variant data for the UShER tskit tree sequence against our MAFFT alignments. There was an exact match between the non-ambiguous nucleotide calls at 27,469 sites. At the remaining 39 sites for which the UShER tree had mutations there were a significant number of gap characters (deletion calls), suggesting that the mismatches could be due to differences in the alignments used as input to UShER. See the notebook validate_usher_tree.ipynb for details.

We then computed intersection ARGs for sc2ts (sc2ts_viridian_inter_v1.2.trees.tsz) and UShER (usher_viridian_inter_v1.2.trees.tsz) by simplifying^43,48^ both to the 2,475,418 samples and 27,507 sites that are shared (the UShER tree has mutations at 27,508 sites and sc2ts ARG has 29,893). As there was no documented means of deriving node dates from the UShER tree, and we wish to visually compare the phylogenetic backbones, we also dated the intersection tree by fixing the sample dates using the “Date_tree” metadata field, and using tsdate (version 0.2.3) to date internal nodes.

All conversions were performed as part of the reproducible pipeline described in the Postprocessing pipeline section. The full UShER tree and the intersection files are available for download in tszip format (see Data and code availability).

#### Comparison of phylogenetic backbones

We compare the non-recombinant parts of the ARG with the UShER topology by simplifying the phylogenies to a smaller number of Pango representative samples. We focus on 1286 Pango lineages comprising those which are defined by perfect “origination events” (Document S1.3). but which are not acknowledged recombinant lineages (i.e. Pango X lineages and their descendants). To ensure that each sample is a tip in the simplified topologies, a representative sample is then chosen as the oldest sample descending from the origination node whose own descendants (if any) are entirely of the focal Pango type. We then further remove 4 lineages which are singleton descendants of a putative ARG recombination event, This results in a simplified ARG of 1282 samples, each sample from a different Pango lineage.

The simplified ARG contains only two recombination nodes, which are the two likely artefactual events discussed in Document S1.12, both of which have breakpoints near the 5’ end of genome. We therefore compare the UShER topology against the first tree in the ARG (covering positions 1–26858; the majority of the genome). To reduce plot density, we consider only those Pango lineages with greater than 10 samples. Figure S3 shows the resulting tree compared with the equivalent dated UShER tree as a tanglegram. We also show subtrees for Delta (Figure S4), BA.2 (Figure S5), and BA.5 (Figure S6).

The order of tips in each plot was chosen to reduce tangling, as calculated using the NeighborNet algorithm from Dendroscope^20,151^, with tanglegrams plotted using the SVG outputting facility built into tskit. See supp_pdf-cophylogeny-tanglegrams.ipynb for details.

#### Comparison of mutational spectra

We calculated the mutational spectra of Alpha, Delta, Omicron BA.1, BA.2, BA.4, and BA.5 in the sc2ts ARG, considering mutations (ignoring indels) above the sample nodes and internal nodes which have Scorpio labels associated with the VOCs (e.g., for Alpha, B.1.1.7-like and B.1.1.7-like+E484K). Note that ~98% of all the mutations in the ARG occur above the nodes that have a Scorpio label associated with these VOCs (excluding the nodes with unassigned Scorpio labels).

We calculated the mutational spectra of the same VOCs in the intersection UShER tree in tskit format using a similar approach. Here, we used the Scorpio sample assignments provided in the Viridian metadata, and therefore restricted to mutations above sample nodes only.

Next, we calculated the mutational spectra of the same VOCs using the mutational counts from an UShER phylogeny analysed by Bloom et al.^78^, which was built using a Nextclade pipeline and has samples collected worldwide up to November, 2022. To obtain the WHO label corresponding to a Nextclade-defined clade, we used the following mapping: 20I, Alpha; 21I and 21J, Delta; 21K, BA.1; 21L, BA.2; 22A, BA.4; and 22B, BA.5. The mutation counts were downloaded from https://github.com/jbloomlab/SARS2-mut-spectrum/.

See the fig_mutational_spectra.ipynb notebook for details.

#### Concordance of recombination events

We compare concordance rates of sc2ts in identifying the Pango lineages of recombinant parents and the location of breakpoint intervals with RecombinHunt^40^ and CovRecomb^39^. We use these two methods because detection results on the Pango X lineages were reported and made available in the original publications. We do not compare here with UShER+RIPPLES+RIVET because curated data on the X lineages is not available for this pipeline (see Document S1.11 and Table S8, however). We use the data from the Pango designation community (https://github.com/cov-lineages/pango-designation/) as the “truth” when comparing performance in identifying parent Pango lineages and breakpoints between the methods. We focus on the 16 Type I recombination events (Table 1) identified by sc2ts for simplicity. While this is certainly biased in favour of sc2ts (i.e., by ignoring likely sc2ts false negatives such as XB), it helps to understand the probable accuracy of sc2ts on the true positives. It is important to emphasise that the “true” truth set among the Pango X lineages is currently unknown.

For RecombinHunt, we obtained two sets of detection results of the Pango X lineages from the supplementary materials of Alfonso et al.^40^. One set of results is based on mutational profiles calculated from a global GISAID dataset, and the other set from a global Nextstrain dataset. The breakpoint intervals identified using RecombinHunt are defined as the left and right recombination-informative sites.

For CovRecomb, we summarized the detection results for the Pango X lineages as follows. First, we obtained the “independent” recombination events identified by Li et al.^41^ (see Table S4 of the study) which correspond to the Pango X lineages (if available). When multiple events corresponded to a Pango X lineage, we took the results of the event which had the highest number of epidemic recombinants. For other Pango X lineages, we downloaded the full list of putative recombinants identified in the study (https://github.com/wuaipinglab/CovRecomb/blob/main/CovRecomb-Global-Version/putative_recombinants/putative%20recombinants.csv). For each of these Pango X lineages, we took the most commonly observed combination of the left parent, the right parent, and the breakpoint interval. The breakpoint intervals are defined as the left and right recombination-informative sites.

The parents of a Pango X inferred using a method are deemed concordant if they have Pango lineage assignments that are at least as specific as those proposed by the Pango designation community. The breakpoint interval of a Pango X inferred using a method is deemed concordant if it overlaps with that proposed by the community by at least one base.

The results are reported in Table S3. See the tab_methods_concordance.ipynb notebook for details.

#### Calculation of Pango lineage distance using pangonet

We define a “pseudo-phylogenetic” distance to quantify the divergence between two Pango lineages. The idea is to count the number of edges separating two given Pango lineages in a pseudo-phylogeny which relates all the designated Pango lineages. To construct this pseudo-phylogeny, we used pangonet (https://github.com/phac-nml/pangonet) which mines the information on the Pango lineages in the “alias_key.json” and “lineage_notes.txt” files defining the designations (https://github.com/cov-lineages/pango-designation). The distance between two Pango lineages is then the number of edges separating the corresponding nodes in this graph.

#### Expected lengths of recombination breakpoint intervals

Recombination breakpoint intervals are imprecise when the recombining parents are identical in sequence at sites spanning the breakpoint. Here, we estimate the interval lengths expected around a breakpoint, given a genome-wide nucleotide substitution rate (*μ*), total time between two samples (*T*), and sufficient support to detect the recombination event. If a breakpoint occurs a fraction *P* along a genome of length *L* and *k* mutations have occurred to the left of the breakpoint, then we expect the nearest mutation to occur *PL/*(*k* + 1) bases to the left. If we require *n* mutations on both sides of the breakpoint, we can calculate the expected value of the interval to the left by summing across a Poisson-distributed number of mutations, conditioned on having at least *n* mutations, using the following equation (where Γ(*x*) is the gamma function, and Γ(*x, y*) is the upper incomplete gamma function):

$$I_{\text{left}}=\frac{L}{nT\mu }\frac{\Gamma \left(1+n\right)-\Gamma \left(1+n,PT\mu \right)}{\Gamma \left(n\right)-\Gamma \left(n,PT\mu \right)}$$

A similar result holds for the right interval (*I*_*right*_), but with *P* replaced with 1 − *P*. Summing the two intervals (*I*_*left*_ + *I*_*right*_) gives the expected interval length. As detectable recombination events tend to be closer to the center of the genome, we use *P* = 0.5 in Figure 4D.

In Figure 4D, we calculated the expected lengths of breakpoint intervals by setting *μ* to ~0.0598 per day. This is based on published estimates of the per-site nucleotide substitution rate of ~ 2 × 10^−6^ per site per day^145,152,153^.

#### Comparison with UShER+RIPPLES

We began with the Viridian UShER tree downloaded from Figshare. To simplify the comparison with sc2ts, we subset this file down to the 2,475,418 samples shared with the sc2ts ARG by running matUtils extract. We then ran ripples-fast^106^ on this file, using -n1 so that all detected recombinants were reported (equivalent to sc2ts). We ran RIPPLES with the parsimony improvement parameter *p* equal to 3 (the default) and 4.

The output of RIPPLES is two text files, “recombination.tsv” and “descendants.tsv“. We use the “descendants.tsv” file, which lists the descendant samples of all recombination events keyed by the recombinant node ID, to match events with sc2ts. Specifically, for each RIPPLES event identified a list of descendant samples, we find the MRCA of that set of samples in the first tree of the sc2ts ARG. The clades are then said to agree if the number of additional samples descending from the sc2ts node is < 2 (Figure S18).

We ran 3SEQ on the RIPPLES recombinants by extracting the alignments for the child and parent sequences for each recombination event. We first obtained the parent node IDs from the “recombination.tsv” file by choosing the most parsimonious event associated with each recombinant node, arbitrarily choosing among the possibilities in the case of ties. We then used this list of child and parent node IDs as input to matUtils extract -v to extract their sequences in VCF format. Following this, we used vcflib^154^ to convert to alignments in FASTA format, as recommended by the UShER documentation (vcf2fasta -f NC_045512v2.fa). We then ran 3SEQ in “full mode” independently for each trio, in the same manner as described when validating the sc2ts recombinants.

The UShER tree is not dated, and so to obtain dates for parent nodes we used sample metadata (Date Tree from the Viridian metadata). For the minority of parent sequences that are not samples, we used the oldest sample descending from a given internal node as a proxy for its date. We used the BTE Python library (https://jmcbroome.github.io/BTE/build/html/index.html) to perform the required traversals on the UShER tree.

The full workflow is encoded in the Snakemake postprocessing pipeline. See Document S1.11 for detailed analysis of the RIPPLES events, and the analysis_ripples.ipynb notebook for full methodological details of the analysis.

### Quantification and statistical analysis

#### Analysing global case counts

To explore the timing of emergence of recombination events, global case count data were downloaded from the COVID-19 Data Repository by the Center for Systems Science and Engineering (CSSE) at Johns Hopkins University^113^ and binned by week from January, 2020 to March, 2023. The proportion of each lineage *i* in each week (*p*_*i*_) was then inferred from the Scorpio designation at each node within the ARG for that week. Genetic variation was measured as the “expected heterozygosity” across lineages, which is the chance that two lineages chosen at random from that week had different Scorpio designations (∑_*i*_ ∑_*j>i*_ 2*p*_*i*_*p*_*j*_). The linear regression models reported in Figure 5 allowed for a non-zero intercept, because there may be unreported cases even without any reported cases. The slopes were highly significant, and the p-values were confirmed by randomly permuting the predictor variable 10,000 times. ANOVAs were conducted to determine which single predictor (*n*, *H*, *n H*, *n*^2^, *n*^2^*H*) explained the most variation in recombination events by week. These statistical tests and graphics were conducted in *Mathematica* 14.2 (Wolfram 2024) and available at https://github.com/jeromekelleher/sc2ts-paper/blob/main/notebooks/TemporalTrends_RecEvent25August2025.nb.

In addition to the analysis of the 354 recombination events that pass QC, we analyzed the relationship between the full set of 855 recombinants and case numbers. These additional recombinants occurred disproportionately during the Delta and first Omicron wave (July, 2021 to February, 2022). Again, the number of recombinant events rose with the global case count (linear regression: 3.1+5.3 10^−7^*x*, *p* = 0.0006) and with the diversity of lineages in each week (linear regression: 0.42 + 18.3*x*, *p* = 2 10^−12^). The single best predictor of recombination events among the predictors tested (*n*, *H*, *n H*, *n*^2^, *n*^2^*H*) was again the product of cases and diversity (*n H*), with an adjusted *R*^2^ of 26.9%, although this was now followed closely by expected heterozygosity on its own (*H*), with an adjusted *R*^2^ of 26.5%. Thus, similar conclusions are reached with either dataset, although filtering the recombination events to the 354 with better support revealed a stronger relationship with case numbers.

## Supplementary Material

Supplement 1

Supplement 2

1

## Figures and Tables

**Table 1:** Events in the ARG associated with Pango X lineages. Each row corresponds to the origin node for a given Pango lineage (Types I, II, and IV, see text) or associated recombination event (Type II). For origin nodes that are recombinant (Type I) or near a recombination event (Type II), the “averted” column shows the number of additional mutations required without recombination, as well as the position of informative sites to the left and right of the breakpoint. Parent lineages shown in boldface are nodes exactly matched by sampled sequences. The descendants column shows the number of descendants by Pango lineage. For Pango lineages (Type III) whose origin node was a descendant of a Type I or II recombination node, the distance (path length and time in days) from the recombination event is shown, as well as the number of mutations directly ancestral to the Pango origin node. Pango X lineages not associated with a recombination node (Type IV) are shown with the parent node and number of mutations.

Type I event: Recombination coinciding with Pango origin node						
		Parent lineage		Interval		
pango	averted	left	right	left	right	descendants
XC	37	**AY.29**	**B.1.1.7**	26768	27390	XC:5
XBR	22	**BN.3.1**	BQ.1.25	22034	22190	XBR:1
XA	16	**B.1.177.18**	**B.1.1.7**	20411	21765	XA:39
XS	11	AY. 103	**BA.1.1**	9054	10449	XS:17
XL	8	**BA.1.17.2**	**BA.2.5**	5925	8393	XL:64
XBG	8	**BA.2.76**	**BA.5.2**	22305	22917	XBG:25
XQ	7	**BA.1.1.15**	**BA.2.9**	4322	5386	XQ:55, BA.2:37, XR:17, XAA:17, XU:1, XAM:21, XAG:6
XBD	7	**BA.2.75.2**	**BA.5.2.1**	23020	24620	XBD:30
XM	6	**BA.1.1**	**BA.2**	17411	21595	XM:26, BA.2:16, XAL:3
XBH	6	**BA.2.1**	BA.2.75.2	15452	22001	XBH:6
XBB	6	**BA.2.10**	BM.1.1.1	22332	22577	XBB:6452, BA.2:1
XY	5	**BA.1.1**	**BA.2**	11538	12880	XY:23
XF	5	**AY.4**	**BA.1**	5387	6402	XF:16
XBF	5	**BA.5.2.1**	CJ.1	5184	9866	XBF:185
XW	4	**BA.1.1.15**	**BA.2**	2833	4321	XW:32
XG	4	**BA.1.17**	**BA.2**	5925	6513	XG:3
Type II event: Recombination closely associated with pango lineage(s)						
		Parent lineage		Interval		
name	averted	left	right	left	right	descendants
Xx	6	**BA.2**	**BA.1.17.2**	24504	26060	BA.2:156, XZ:48, XAP:20, XAC:18, XAE:9, XAD:2
XE/XH	6	**BA.1.17.2**	**BA.2**	10448	11283	XE:1116, BA.2:37, XH:2, XAF:1
XBM	6	**BA.2.76**	**BF.3**	22305	22917	XBM:10, BF.3:2
XJ	5	**BA.1.17.2**	**BA.2**	13196	17410	XJ:68, BA.2:17
XAF	5	**BA.1.1**	**BA.2**	10199	10447	BA.2:35, XAF:1
Type III events: Pango origin nodes derived from Type I and II events						
pango	event	path len	time	mutations		descendants
XAF	XAF	5	140	8		XAF:1
XBM	XBM	1	23	1		XBM:10
XE	XE/XH	1	19	2		XE:1116
XH	XE/XH	1	55	7		XH:2
XJ	XJ	1	5	2		XJ:68, BA.2:3
XAL	XM	3	65	2		XAL:3
XAA	XQ	6	33	3		XAA:17
XAG	XQ	6	53	4		XAG:6
XAM	XQ	6	40	3		XAM:21
XR	XQ	3	11	4		XR:17, BA.2:1
XU	XQ	6	103	13		XU:1
XAE	Xx	2	47	7		XAE:9
XAP	Xx	4	66	1		XAP:20
XZ	Xx	4	48	1		XZ:48
Type IV events: Non recombinants in the ARG						
pango	parent			mutations		descendants
XAS	BA.4			1		XAS:77
XAU	BA.2			1		XAU:8
XBK	CJ.1			1		XBK:7
XBQ	CJ.1			1		XBQ:14
XN	**BA.2**			1		XN:120, BA.2:1
XP	**BA.1.1**			1		XP:45
XB	B.1			2		XB:192
XAV	BA.5.1.24			3		XAV:13
XAZ	**BA.5**			3		XAZ:133
XBE	BA.5.2			3		XBE:65
XAN	**BA.5.1**			5		XAN:7
XAJ	**BA.2.12**			11	5	XAJ:18

## References

[1] Felsenstein J. (2004). Inferring Phylogenies. Sinauer Associates.

[2] Posada D., and Crandall K.A. (2002). The effect of recombination on the accuracy of phylogeny estimation. Journal of Molecular Evolution 54, 396–402. 11847565 10.1007/s00239-001-0034-9

[3] Schierup M.H., and Hein J. (2000). Consequences of recombination on traditional phylogenetic analysis. Genetics 156, 879–891. 11014833 10.1093/genetics/156.2.879 PMC1461297

[4] Hedge J., and Wilson D.J. (2014). Bacterial phylogenetic reconstruction from whole genomes is robust to recombination but demographic inference is not. MBio 5, 10–1128. 10.1128/mBio.02158-14 PMC4251999 25425237

[5] Rannala B. (2025). Recombination and phylogenetic inference. Evolutionary Journal of the Linnean Society 4, kzaf016. 40978768 10.1093/evolinnean/kzaf016 PMC12448323

[6] Shu Y., and McCauley J. (2017). GISAID: Global initiative on sharing all influenza data – from vision to reality. Eurosurveillance 22. 10.2807/1560-7917.ES.2017.22.13.30494 PMC5388101 28382917

[7] Jolley K.A., Bray J.E., and Maiden M.C. (2018). Open-access bacterial population genomics: BIGSdb software, the PubMLST.org website and their applications. Wellcome Open Research 3, 124. 30345391 10.12688/wellcomeopenres.14826.1 PMC6192448

[8] Apetrei C., Hahn B., Rambaut A., Wolinsky S., Brister J.R., Keele B., and Faser C.,eds. (2022). HIV Sequence Compendium 2020. Los Alamos, New Mexico: Los Alamos National Laboratory, Theoretical Biology and Biophysics. Technical Report: LA-UR-23-2190.

[9] Consortium CRyPTIC (2022). A data compendium associating the genomes of 12,289 mycobacterium tuberculosis isolates with quantitative resistance phenotypes to 13 antibiotics. PLoS Biology 20, e3001721. 35944069 10.1371/journal.pbio.3001721 PMC9363010

[10] Dyer N.P., Päuker B., Baxter L., Gupta A., Bunk B., Overmann J., Diricks M., Dreyer V., Niemann S., Holt K.E. (2025). Enterobase in 2025: Exploring the genomic epidemiology of bacterial pathogens. Nucleic Acids Research 53, D757–D762. 39441072 10.1093/nar/gkae902 PMC11701629

[11] Suchard M.A., Lemey P., Baele G., Ayres D.L., Drummond A.J., and Rambaut A. (2018). Bayesian phylogenetic and phylodynamic data integration using BEAST 1.10. Virus Evolution 4, vey016. 29942656 10.1093/ve/vey016 PMC6007674

[12] Bouckaert R., Vaughan T.G., Barido-Sottani J., Duchêne S., Fourment M., Gavryushkina A., Heled J., Jones G., Kühnert D., De Maio N. (2019). BEAST 2.5: An advanced software platform for Bayesian evolutionary analysis. PLoS Computational Biology 15, e1006650. 30958812 10.1371/journal.pcbi.1006650 PMC6472827

[13] Minh B.Q., Schmidt H.A., Chernomor O., Schrempf D., Woodhams M.D., Von Haeseler A., and Lanfear R. (2020). IQ-TREE 2: New models and efficient methods for phylogenetic inference in the genomic era. Molecular Biology and Evolution 37, 1530–1534. 32011700 10.1093/molbev/msaa015 PMC7182206

[14] Price M.N., Dehal P.S., and Arkin A.P. (2009). FastTree: Computing large minimum evolution trees with profiles instead of a distance matrix. Molecular Biology and Evolution 26, 1641–1650. 19377059 10.1093/molbev/msp077 PMC2693737

[15] Didelot X., Lawson D., Darling A., and Falush D. (2010). Inference of homologous recombination in bacteria using whole-genome sequences. Genetics 186, 1435–1449. 20923983 10.1534/genetics.110.120121 PMC2998322

[16] Vaughan T.G., Welch D., Drummond A.J., Biggs P.J., George T., and French N.P. (2017). Inferring ancestral recombination graphs from bacterial genomic data. Genetics 205, 857–870. 28007885 10.1534/genetics.116.193425 PMC5289856

[17] Müller N.F., Stolz U., Dudas G., Stadler T., and Vaughan T.G. (2020). Bayesian inference of reassortment networks reveals fitness benefits of reassortment in human influenza viruses. Proceedings of the National Academy of Sciences 117, 17104–17111. 10.1073/pnas.1918304117 PMC7382287 32631984

[18] Guo F., Carbone I., and Rasmussen D.A. (2022). Recombination-aware phylogeographic inference using the structured coalescent with ancestral recombination. PLOS Computational Biology 18, e1010422. 35984849 10.1371/journal.pcbi.1010422 PMC9447913

[19] Müller N.F., Kistler K.E., and Bedford T. (2022). A Bayesian approach to infer recombination patterns in coronaviruses. Nature Communications 13, 4186. 10.1038/s41467-022-31749-8 PMC9297283 35859071

[20] Huson D.H., and Scornavacca C. (2012). Dendroscope 3: An interactive tool for rooted phylogenetic trees and networks. Systematic Biology 61, 1061–1067. 22780991 10.1093/sysbio/sys062

[21] Vaughan T.G. (2017). IcyTree: Rapid browser-based visualization for phylogenetic trees and networks. Bioinformatics 33, 2392–2394. 28407035 10.1093/bioinformatics/btx155 PMC5860111

[22] Solís-Lemus C., Bastide P., and Ané C. (2017). PhyloNetworks: A package for phylogenetic networks. Molecular Biology and Evolution 34, 3292–3298. 28961984 10.1093/molbev/msx235

[23] Rasmussen D.A., and Guo F. (2023). Espalier: Efficient tree reconciliation and ancestral recombination graphs reconstruction using maximum agreement forests. Systematic Biology 72, 1154–1170. 37458753 10.1093/sysbio/syad040 PMC10627558

[24] Turakhia Y., Thornlow B., Hinrichs A.S., De Maio N., Gozashti L., Lanfear R., Haussler D., and Corbett-Detig R. (2021). Ultrafast Sample placement on Existing tRees (UShER) enables real-time phylogenetics for the SARS-CoV-2 pandemic. Nature Genetics 53, 809–816. 33972780 10.1038/s41588-021-00862-7 PMC9248294

[25] De Maio N., Kalaghatgi P., Turakhia Y., Corbett-Detig R., Minh B.Q., and Goldman N. (2023). Maximum likelihood pandemic-scale phylogenetics. Nature Genetics 55, 746–752. 37038003 10.1038/s41588-023-01368-0 PMC10181937

[26] McBroome J., Thornlow B., Hinrichs A.S., Kramer A., De Maio N., Goldman N., Haussler D., Corbett-Detig R., and Turakhia Y. (2021). A daily-updated database and tools for comprehensive SARS-CoV-2 mutation-annotated trees. Molecular Biology and Evolution 38, 5819–5824. 34469548 10.1093/molbev/msab264 PMC8662617

[27] Kramer A.M., Thornlow B., Ye C., De Maio N., McBroome J., Hinrichs A.S., Lanfear R., Turakhia Y., and Corbett-Detig R. (2023). Online phylogenetics with matoptimize produces equivalent trees and is dramatically more efficient for large SARS-CoV-2 phylogenies than de novo and maximum-likelihood implementations. Systematic Biology 72, 1039–1051. 37232476 10.1093/sysbio/syad031 PMC10627557

[28] Hinrichs A., Ye C., Turakhia Y., and Corbett-Detig R. (2024). The ongoing evolution of UShER during the SARS-CoV-2 pandemic. Nature Genetics 56, 4–7. 38155331 10.1038/s41588-023-01622-5

[29] Jackson B., Boni M.F., Bull M.J., Colleran A., Colquhoun R.M., Darby A.C., Haldenby S., Hill V., Lucaci A., McCrone J.T., Nicholls S.M., O’Toole Á., Pacchiarini N., Poplawski R., Scher E., Todd F., Webster H.J., Whitehead M., Wierzbicki C., Loman N.J., Connor T.R., Robertson D.L., Pybus O.G., and Rambaut A. (2021). Generation and transmission of interlineage recombinants in the SARS-CoV-2 pandemic. Cell 184, 5179–5188.e8. 34499854 10.1016/j.cell.2021.08.014 PMC8367733

[30] VanInsberghe D., Neish A.S., Lowen A.C., and Koelle K. (2021). Recombinant SARS-CoV-2 genomes circulated at low levels over the first year of the pandemic. Virus Evolution 7, veab059. 36793768 10.1093/ve/veab059 PMC8344435

[31] Focosi D., and Maggi F. (2022). Recombination in coronaviruses, with a focus on SARS-CoV-2. Viruses 14, 1239. 35746710 10.3390/v14061239 PMC9228924

[32] Gutierrez B., Castelán Sánchez H.G., Candido D.d.S., Jackson B., Fleishon S., Houzet R., Ruis C., Delaye L., Faria N.R., Rambaut A., Pybus O.G., and Escalera-Zamudio M. (2022). Emergence and widespread circulation of a recombinant SARS-CoV-2 lineage in north america. Cell Host & Microbe 30, 1112–1123.e3. 35853454 10.1016/j.chom.2022.06.010 PMC9212848

[33] Ignatieva A., Hein J., and Jenkins P.A. (2022). Ongoing recombination in SARS-CoV-2 revealed through genealogical reconstruction. Molecular Biology and Evolution 39, msac028. 35106601 10.1093/molbev/msac028 PMC8841603

[34] Tamura T., Ito J., Uriu K., Zahradnik J., Kida I., Anraku Y., Nasser H., Shofa M., Oda Y., Lytras S. (2023). Virological characteristics of the SARS-CoV-2 XBB variant derived from recombination of two Omicron subvariants. Nature Communications 14, 2800. 10.1038/s41467-023-38435-3 PMC10187524 37193706

[35] Roemer C., Sheward D.J., Hisner R., Gueli F., Sakaguchi H., Frohberg N., Schoenmakers J., Sato K., O’Toole Á., Rambaut A., Pybus O.G., Ruis C., Murrell B., and Peacock T.P. (2023). SARS-CoV-2 evolution in the Omicron era. Nature Microbiology 8, 1952–1959. 10.1038/s41564-023-01504-w 37845314

[36] Shiraz R., and Tripathi S. (2023). Enhanced recombination among omicron subvariants of SARS-CoV-2 contributes to viral immune escape. Journal of Medical Virology 95, e28519. 36691935 10.1002/jmv.28519

[37] Varabyou A., Pockrandt C., Salzberg S.L., and Pertea M. (2021). Rapid detection of inter-clade recombination in SARS-CoV-2 with Bolotie. Genetics 218, iyab074. 33983397 10.1093/genetics/iyab074 PMC8194586

[38] Turakhia Y., Thornlow B., Hinrichs A., McBroome J., Ayala N., Ye C., Smith K., De Maio N., Haussler D., Lanfear R., and Corbett-Detig R. (2022). Pandemic-scale phylogenomics reveals the SARS-CoV-2 recombination landscape. Nature 609, 994–997. 35952714 10.1038/s41586-022-05189-9 PMC9519458

[39] Zhou Z.J., Yang C.H., Ye S.B., Yu X.W., Qiu Y., and Ge X.Y. (2023). VirusRecom: An information-theory-based method for recombination detection of viral lineages and its application on SARS-CoV-2. Briefings in Bioinformatics 24, bbac513. 36567622 10.1093/bib/bbac513

[40] Alfonsi T., Bernasconi A., Chiara M., and Ceri S. (2024). Data-driven recombination detection in viral genomes. Nature Communications 15, 3313. 10.1038/s41467-024-47464-5 PMC11024102 38632281

[41] Li J.Y., Wang H.Y., Cheng Y.X., Ji C., Weng S., Han N., Yang R., Zhou H.Y., and Wu A. (2024). Comprehensive detection and dissection of interlineage recombination events in the SARS-CoV-2 pandemic. Virus Evolution 10, veae074. 39399153 10.1093/ve/veae074 PMC11470760

[42] Hadfield J., Megill C., Bell S.M., Huddleston J., Potter B., Callender C., Sagulenko P., Bedford T., and Neher R.A. (2018). Nextstrain: Real-time tracking of pathogen evolution. Bioinformatics 34, 4121–4123. 29790939 10.1093/bioinformatics/bty407 PMC6247931

[43] Wong Y., Ignatieva A., Koskela J., Gorjanc G., Wohns A.W., and Kelleher J. (2024). A general and efficient representation of ancestral recombination graphs. Genetics 228, iyae100. 39013109 10.1093/genetics/iyae100 PMC11373519

[44] Hudson R.R. (1983). Properties of a neutral allele model with intragenic recombination.Theoretical Population Biology 23, 183–201. 6612631 10.1016/0040-5809(83)90013-8

[45] Griffiths R.C. (1991). The two-locus ancestral graph. Lecture Notes-Monograph Series 18, 100–117.

[46] Griffiths R.C., and Marjoram P. (1997). An ancestral recombination graph. In Donnelly P., and Tavaré S., eds. Progress in Population Genetics and Human Evolution, IMA Volumes in Mathematics and its Applications vol. 87 pp. 257–270. Berlin: Springer-Verlag pp. 257–270.

[47] Kelleher J., Etheridge A.M., and McVean G. (2016). Efficient coalescent simulation and genealogical analysis for large sample sizes. PLOS Computational Biology 12, e1004842. 27145223 10.1371/journal.pcbi.1004842 PMC4856371

[48] Kelleher J., Thornton K.R., Ashander J., and Ralph P.L. (2018). Efficient pedigree recording for fast population genetics simulation. PLOS Computational Biology 14, 1–21. 10.1371/journal.pcbi.1006581 PMC6233923 30383757

[49] Haller B.C., and Messer P.W. (2019). SLiM 3: Forward genetic simulations beyond the Wright–Fisher model. Molecular Biology and Evolution 36, 632–637. 30517680 10.1093/molbev/msy228 PMC6389312

[50] Gopalan S., Rodrigues M.F., Ralph P.L., and Haller B.C. (2025). Bridging forward-in-time and coalescent simulations using pyslim. bioRxiv. 2025.09.30.679676.

[51] Rasmussen M.D., Hubisz M.J., Gronau I., and Siepel A. (2014). Genome-wide inference of ancestral recombination graphs. PLOS Genetics 10, e1004342. 24831947 10.1371/journal.pgen.1004342 PMC4022496

[52] Speidel L., Forest M., Shi S., and Myers S.R. (2019). A method for genome-wide genealogy estimation for thousands of samples. Nature Genetics 51, 1321–1329. 31477933 10.1038/s41588-019-0484-x PMC7610517

[53] Kelleher J., Wong Y., Wohns A.W., Fadil C., Albers P.K., and McVean G. (2019). Inferring whole-genome histories in large population datasets. Nature Genetics 51, 1330–1338. 31477934 10.1038/s41588-019-0483-y PMC6726478

[54] Wohns A.W., Wong Y., Jeffery B., Akbari A., Mallick S., Pinhasi R., Patterson N., Reich D., Kelleher J., and McVean G. (2022). A unified genealogy of modern and ancient genomes. Science 375, eabi8264. 35201891 10.1126/science.abi8264 PMC10027547

[55] Zhang B.C., Biddanda A., Gunnarsson Á.F., Cooper F., and Palamara P.F. (2023). Biobank-scale inference of ancestral recombination graphs enables genealogical analysis of complex traits. Nature Genetics 55, 768–776. 37127670 10.1038/s41588-023-01379-x PMC10181934

[56] Gunnarsson Á.F., Zhu J., Zhang B.C., Tsangalidou Z., Allmont A., and Palamara P.F. (2024). A scalable approach for genome-wide inference of ancestral recombination graphs. bioRxiv. 2024.08.31.610248.

[57] Deng Y., Nielsen R., and Song Y.S. (2025). Robust and accurate Bayesian inference of genome-wide genealogies for hundreds of genomes. Nature Genetics 57, 2124–2135. 40921789 10.1038/s41588-025-02317-9 PMC12425808

[58] Brandt D.Y.C., Huber C.D., Chiang C.W.K., and Ortega-Del Vecchyo D. (2024). The promise of inferring the past using the ancestral recombination graph. Genome Biology and Evolution 16, evae005. 38242694 10.1093/gbe/evae005 PMC10834162

[59] Lewanski A.L., Grundler M.C., and Bradburd G.S. (2024). The era of the ARG: An introduction to ancestral recombination graphs and their significance in empirical evolutionary genomics. PLOS Genetics 20, e1011110. 38236805 10.1371/journal.pgen.1011110 PMC10796009

[60] Nielsen R., Vaughn A.H., and Deng Y. (2025). Inference and applications of ancestral recombination graphs. Nature Reviews Genetics 26, 47–58. 10.1038/s41576-024-00772-4 PMC12036574 39349760

[61] Minichiello M.J., and Durbin R. (2006). Mapping trait loci by use of inferred ancestral recombination graphs. American Journal of Human Genetics 79, 910–922. 17033967 10.1086/508901 PMC1698562

[62] Ralph P., Thornton K., and Kelleher J. (2020). Efficiently summarizing relationships in large samples: A general duality between statistics of genealogies and genomes. Genetics 215, 779–797. 32357960 10.1534/genetics.120.303253 PMC7337078

[63] Baumdicker F., Bisschop G., Goldstein D., Gower G., Ragsdale A.P., Tsambos G., Zhu S., Eldon B., Ellerman E.C., Galloway J.G. (2022). Efficient ancestry and mutation simulation with msprime 1.0. Genetics 220, iyab229. 34897427 10.1093/genetics/iyab229 PMC9176297

[64] Petr M., Haller B.C., Ralph P.L., and Racimo F. (2023). slendr: A framework for spatiotemporal population genomic simulations on geographic landscapes. Peer Community Journal 3, e121. 38984034 10.24072/pcjournal.354 PMC11233137

[65] Tsambos G., Kelleher J., Ralph P., Leslie S., and Vukcevic D. (2023). Link-ancestors: Fast simulation of local ancestry with tree sequence software. Bioinformatics Advances 3, vbad163. 38033661 10.1093/bioadv/vbad163 PMC10682689

[66] Tagami D., Bisschop G., and Kelleher J. (2024). tstrait: a quantitative trait simulator for ancestral recombination graphs. Bioinformatics 40, btae334. 38796683 10.1093/bioinformatics/btae334 PMC11784591

[67] Karthikeyan S., Jeffery B., Mbuli-Robertson D., and Kelleher J. (2025). Tsbrowse: An interactive browser for ancestral recombination graphs. Bioinformatics 41, btaf393. 40650922 10.1093/bioinformatics/btaf393 PMC12342996

[68] Kitchens J., and Wong Y. (2025). tskit arg visualizer: Interactive plotting of ancestral recombination graphs. arXiv. 2508.03958. 10.1093/bioadv/vbaf302 PMC12701794 41394082

[69] Talbot C., and Bradburd G. (2025). ARGscape: A modular, interactive tool for manipulation of spatiotemporal ancestral recombination graphs. arXiv. 2510.07255. 10.1093/bioinformatics/btag420 PMC13371762 42348238

[70] Mahmoudi A., Koskela J., Kelleher J., Chan Y.b., and Balding D. (2022). Bayesian inference of ancestral recombination graphs. PLOS Computational Biology 18, e1009960. 35263345 10.1371/journal.pcbi.1009960 PMC8936483

[71] Martin S.H. (2025). A model-free method for genealogical inference without phasing and its application for topology weighting. Genetics pp. iyaf181. 40916405 10.1093/genetics/iyaf181 PMC12774849

[72] Pieszko T., Kelleher J., Wilson C.G., and Barraclough T.G. (2025). Detecting and quantifying rare sex in natural populations. bioRxiv. 2025.06.03.657731.

[73] Fan C., Mancuso N., and Chiang C.W. (2022). A genealogical estimate of genetic relationships. American Journal of Human Genetics 109, 812–824. 35417677 10.1016/j.ajhg.2022.03.016 PMC9118131

[74] Nowbandegani P.S., Wohns A.W., Ballard J.L. (2023). Extremely sparse models of linkage disequilibrium in ancestrally diverse association studies. Nature Genetics 55, 1494–1502. 37640881 10.1038/s41588-023-01487-8

[75] Fritze H., Pope N., Kelleher J., and Ralph P. (2024). A forest is more than its trees: Haplotypes and inferred ARGs. bioRxiv. 2024.11.30.626138.

[76] Hunt M., Hinrichs A.S., Anderson D., Karim L., Dearlove B.L., Knaggs J., Constantinides B., Fowler P.W., Rodger G., Street T. (2024). Addressing pandemic-wide systematic errors in the SARS-CoV-2 phylogeny. bioRxiv. 2024.04.29.591666. 10.1038/s41592-025-02947-1 PMC12982125 41663577

[77] Rambaut A., Holmes E.C., O’Toole Á., Hill V., McCrone J.T., Ruis C., du Plessis L., and Pybus O.G. (2020). A dynamic nomenclature proposal for SARS-CoV-2 lineages to assist genomic epidemiology. Nature Microbiology 5, 1403–1407. 10.1038/s41564-020-0770-5 PMC7610519 32669681

[78] Bloom J.D., Beichman A.C., Neher R.A., and Harris K. (2023). Evolution of the SARS-CoV-2 mutational spectrum. Molecular Biology and Evolution 40, msad085. 37039557 10.1093/molbev/msad085 PMC10124870

[79] Li X., Yan H., Wong G., Ouyang W., and Cui J. (2023). Identifying featured indels associated with SARS-CoV-2 fitness. Microbiology Spectrum 11, e02269–23. 10.1128/spectrum.02269-23 PMC10580940 37698427

[80] Czech E., Tyler W., White T., Jeffery B., Millar T.R., Elsworth B., Guez J., Hancox J., Karczewski K.J., Miles A., Tallman S., Unneberg P., Wojdyla R., Zabad S., Hammerbacher J., and Kelleher J. (2025). Analysis-ready VCF at Biobank scale using Zarr. GigaScience 14, giaf049. 40451243 10.1093/gigascience/giaf049 PMC12127038

[81] Li N., and Stephens M. (2003). Modeling linkage disequilibrium and identifying recombination hotspots using single-nucleotide polymorphism data. Genetics 165, 2213–2233. 14704198 10.1093/genetics/165.4.2213 PMC1462870

[82] Delaneau O., Zagury J.F., Robinson M.R., Marchini J.L., and Dermitzakis E.T. (2019). Accurate, scalable and integrative haplotype estimation. Nature Communications 10, 5436. 10.1038/s41467-019-13225-y PMC6882857 31780650

[83] Browning B.L., Zhou Y., and Browning S.R. (2018). A One-Penny imputed genome from Next-Generation reference panels. American Journal of Human Genetics 103, 338–348. 30100085 10.1016/j.ajhg.2018.07.015 PMC6128308

[84] Donnelly P., and Leslie S. (2010). The coalescent and its descendants. arXiv. 1006.1514.

[85] Harris C.R., Millman K.J., van der Walt S.J., Gommers R., Virtanen P., Cournapeau D., Wieser E., Taylor J., Berg S., Smith N.J. (2020). Array programming with NumPy. Nature 585, 357–362. 32939066 10.1038/s41586-020-2649-2 PMC7759461

[86] Virtanen P., Gommers R., Oliphant T.E., Haberland M., Reddy T., Cournapeau D., Burovski E., Peterson P., Weckesser W., Bright J. (2020). SciPy 1.0: Fundamental algorithms for scientific computing in python. Nature Methods 17, 261–272. 32015543 10.1038/s41592-019-0686-2 PMC7056644

[87] McKinney W. (2010). Data Structures for Statistical Computing in Python. In der Walt Stéfan van, and Millman Jarrod, eds. Proceedings of the 9th Python in Science Conference. pp. 56–61.

[88] Lam S.K., Pitrou A., and Seibert S. (2015). Numba: a LLVM-based Python JIT compiler. In Proceedings of the Second Workshop on the LLVM Compiler Infrastructure in HPC. pp. 1–6.

[89] Shirley M.D., Ma Z., Pedersen B.S., and Wheelan S.J. Efficient “pythonic” access to FASTA files using pyfaidx. Tech. Rep. PeerJ PrePrints (2015).

[90] Kunzmann P., and Hamacher K. (2018). Biotite: A unifying open source computational biology framework in Python. BMC Bioinformatics 19, 346. 30285630 10.1186/s12859-018-2367-z PMC6167853

[91] Kunzmann P., Müller T.D., Greil M., Krumbach J.H., Anter J.M., Bauer D., Islam F., and Hamacher K. (2023). Biotite: New tools for a versatile Python bioinformatics library. BMC Bioinformatics 24, 236. 37277726 10.1186/s12859-023-05345-6 PMC10243083

[92] O’Toole Á., Scher E., Underwood A., Jackson B., Hill V., McCrone J.T., Colquhoun R., Ruis C., Abu-Dahab K., Taylor B. (2021). Assignment of epidemiological lineages in an emerging pandemic using the pangolin tool. Virus Evolution 7, veab064. 34527285 10.1093/ve/veab064 PMC8344591

[93] Pope N. (2025). In preparation.

[94] Sanderson T. (2021). Chronumental: Time tree estimation from very large phylogenies. bioRxiv. 2021.10.27.465994.

[95] Rambaut A., Loman N., Pybus O., Barclay W., Barrett J., Carabelli A., Connor T., Peacock T., Robertson D.L., and Volz E. (2020). Preliminary genomic characterisation of an emergent SARS-CoV-2 lineage in the uk defined by a novel set of spike mutations. https://virological.org/t/preliminary-genomic-characterisation-of-an-emergent-sars-cov-2-lineage-in-the-uk-defined-563.

[96] European Centre for Disease Control (2021). Threat assessment brief: Emergence of SARS-CoV-2 B.1.617 variants in India and situation in the EU/EEA. https://www.ecdc.europa.eu/en/publications-data/threat-assessment-emergence-sars-cov-2-b1617-variants.

[97] Cherian S., Potdar V., Jadhav S., Yadav P., Gupta N., Das M., Rakshit P., Singh S., Abraham P., Panda S. (2021). SARS-CoV-2 spike mutations, L452R, T478K, E484Q and P681R, in the second wave of COVID-19 in Maharashtra, India. Microorganisms 9, 1542. 34361977 10.3390/microorganisms9071542 PMC8307577

[98] Farinholt T., Doddapaneni H., Qin X., Menon V., Meng Q., Metcalf G., Chao H., Gingras M.C., Avadhanula V., Farinholt P., Agrawal C., Muzny D.M., Piedra P.A., Gibbs R.A., and Petrosino J. (2021). Transmission event of SARS-CoV-2 Delta variant reveals multiple vaccine breakthrough infections. BMC Medicine 19, 255. 34593004 10.1186/s12916-021-02103-4 PMC8483940

[99] Stern A., Fleishon S., Kustin T., Dotan E., Mandelboim M., Erster O., Israel Consortium of SARS-CoV-2 Sequencing, Mendelson E., Mor O., and Zuckerman N.S. (2021). The unique evolutionary dynamics of the SARS-CoV-2 Delta variant. medRxiv. 2021.08.05.21261642.

[100] Dor G., Wilkinson E., Martin D.P., Moir M., Tshiabuila D., Kekana D., Ntozini B., Joseph R., Iranzadeh A., Nyaga M.M. (2025). Tracing the spatial origins and spread of SARS-CoV-2 Omicron lineages in South Africa. Nature Communications 16, 4937. 10.1038/s41467-025-60081-0 PMC12120024 40436832

[101] Rambaut A. (2021). Proposal to split B.1.1.529 to incorporate a newly characterised sibling lineage. https://github.com/cov-lineages/pango-designation/issues/361.

[102] Jeronimo P.M.C., Aksenen C.F., Duarte I.O., Lins R.D., and Miyajima F. (2023). Evolutionary deletions within the SARS-CoV-2 genome as signature trends for virus fitness and adaptation. Journal of Virology 98, e01404–23. 38088350 10.1128/jvi.01404-23 PMC10804945

[103] McCarthy K.R., Rennick L.J., Nambulli S., Robinson-McCarthy L.R., Bain W.G., Haidar G., and Duprex W.P. (2021). Recurrent deletions in the SARS-CoV-2 spike glycoprotein drive antibody escape. Science 371, 1139–1142. 33536258 10.1126/science.abf6950 PMC7971772

[104] Walker A.S., Vihta K.D., Gethings O., Pritchard E., Jones J., House T., Bell I., Bell J.I., Newton J.N., Farrar J., Diamond I., Studley R., Rourke E., Hay J., Hopkins S., Crook D., Peto T., Matthews P.C., Eyre D.W., Stoesser N., and Pouwels K.B. (2021). Tracking the emergence of SARS-CoV-2 Alpha variant in the United Kingdom. New England Journal of Medicine 385, 2582–2585. 34879193 10.1056/NEJMc2103227 PMC8693687

[105] Meng B., Kemp S.A., Papa G., Datir R., Ferreira I.A., Marelli S., Harvey W.T., Lytras S., Mohamed A., Gallo G. (2021). Recurrent emergence of SARS-CoV-2 spike deletion H69/V70 and its role in the Alpha variant B.1.1.7. Cell Reports 35, 109292. 34166617 10.1016/j.celrep.2021.109292 PMC8185188

[106] Smith K., Ye C., and Turakhia Y. (2023). Tracking and curating putative SARS-CoV-2 recombinants with RIVET. Bioinformatics 39, btad538. 37651464 10.1093/bioinformatics/btad538 PMC10493179

[107] Turakhia Y., De Maio N., Thornlow B., Gozashti L., Lanfear R., Walker C.R., Hinrichs A.S., Fernandes J.D., Borges R., Slodkowicz G., Weilguny L., Haussler D., Goldman N., and Corbett-Detig R. (2020). Stability of SARS-CoV-2 phylogenies. PLOS Genetics 16, e1009175. 33206635 10.1371/journal.pgen.1009175 PMC7721162

[108] De Maio N., Walker C., Borges R., Weilguny L., Slodkowicz G., and Goldman N. (2020). Issues with SARS-CoV-2 sequencing data. https://virological.org/t/issues-with-sars-cov-2-sequencing-data/473. 10.1371/journal.pgen.1009175 PMC7721162 33206635

[109] Sanderson T., and Barrett J.C. (2021). Variation at Spike position 142 in SARS-CoV-2 Delta genomes is a technical artifact caused by dropout of a sequencing amplicon. Wellcome Open Research 6, 305. 35634532 10.12688/wellcomeopenres.17295.1 PMC9117943

[110] Lam H.M., Ratmann O., and Boni M.F. (2018). Improved algorithmic complexity for the 3SEQ recombination detection algorithm. Molecular Biology and Evolution 35, 247–251. 29029186 10.1093/molbev/msx263 PMC5850291

[111] O’Toole Á., Aziz A., and Maloney D. (2024). Publication-ready single nucleotide polymorphism visualization with snipit. Bioinformatics 40, btae510. 39137137 10.1093/bioinformatics/btae510 PMC11349183

[112] Bobay L.M., O’Donnell A.C., and Ochman H. (2020). Recombination events are concentrated in the spike protein region of betacoronaviruses. PLoS Genetics 16, e1009272. 33332358 10.1371/journal.pgen.1009272 PMC7775116

[113] Dong E., Du H., and Gardner L. (2020). An interactive web-based dashboard to track COVID-19 in real time. The Lancet Infectious Diseases 20, 533–534. 32087114 10.1016/S1473-3099(20)30120-1 PMC7159018

[114] Wang Y., and Kumbhakar S.C. (2025). Covid-19 under-reporting: Spillovers and stringent containment strategies of global cases. Journal of Productivity Analysis 63, 87–106.

[115] Burel E., Colson P., Lagier J.C., Levasseur A., Bedotto M., Lavrard-Meyer P., Fournier P.E., La Scola B., and Raoult D. (2022). Sequential appearance and isolation of a SARS-CoV-2 recombinant between two major SARS-CoV-2 variants in a chronically infected immunocompromised patient. Viruses 14, 1266. 35746737 10.3390/v14061266 PMC9227898

[116] Anderson-Trocmé L., Nelson D., Zabad S., Diaz-Papkovich A., Kryukov I., Baya N., Touvier M., Jeffery B., Dina C., Vézina H. (2023). On the genes, genealogies, and geographies of Quebec. Science 380, 849–855. 37228217 10.1126/science.add5300

[117] Mejia-Garcia A., Diaz-Papkovich A., Sillon G., D’Agostino D., Chong A.L., Chong G., Lo K.S., Baret L., Hamel N., Chapdelaine V. (2025). Using the ancestral recombination graph to study the history of rare variants in founder populations. American Journal of Human Genetics. 10.1016/j.ajhg.2025.10.005 PMC12808987 41172993

[118] Grundler M.C., Terhorst J., and Bradburd G.S. (2025). A geographic history of human genetic ancestry. Science 387, 1391–1397. 40146820 10.1126/science.adp4642 PMC12132082

[119] Lehmann B., Lee H., Anderson-Trocmé L., Kelleher J., Gorjanc G., and Ralph P.L. (2025). On ARGs, pedigrees, and genetic relatedness matrices. Genetics pp. iyaf219. 41061669 10.1093/genetics/iyaf219 PMC12774834

[120] Terhorst J. (2025). Accelerated Bayesian inference of population size history from recombining sequence data. Nature Genetics 57, 2570–2577. 40954248 10.1038/s41588-025-02323-x PMC12513830

[121] Haddox H.K., Angehrn G., Sesta L., Jennings-Shaffer C., Temple S.D., Galloway J.G., Hinrichs A.S., DeWitt W.S., Bloom J.D., M. F.A. IV, and Neher R.A. (2025). The mutation rate of SARS-CoV-2 is highly variable between sites and is influenced by sequence context, genomic region, and RNA structure. Nucleic Acids Research 53, gkaf503. 40503682 10.1093/nar/gkaf503 PMC12159741

[122] Simmonds P. (2024). C→U transition biases in SARS-CoV-2: Still rampant 4 years from the start of the COVID-19 pandemic. mBio 15, e02493–24. 39475243 10.1128/mbio.02493-24 PMC11633203

[123] Kumar S., and Hedges S.B. (2016). Advances in time estimation methods for molecular data. Molecular Biology and Evolution 33, 863–869. 26882983 10.1093/molbev/msw026 PMC5870647

[124] Lancet T. (2021). Genomic sequencing in pandemics. The Lancet 397, 445. 10.1016/S0140-6736(21)00257-9 PMC7906659 33549175

[125] Chen Z., Azman A.S., Chen X., Zou J., Tian Y., Sun R., Xu X., Wu Y., Lu W., Ge S., Zhao Z., Yang J., Leung D.T., Domman D.B., and Yu H. (2022). Global landscape of SARS-CoV-2 genomic surveillance and data sharing. Nature Genetics 54, 499–507. 35347305 10.1038/s41588-022-01033-y PMC9005350

[126] Bisschop G., Kelleher J., and Ralph P. (2025). Likelihoods for a general class of args under the smc. Genetics pp. iyaf103. 40439129 10.1093/genetics/iyaf103 PMC12774825

[127] Kelleher J., and Lohse K. (2020). Coalescent simulation with msprime. In Dutheil J.Y., ed. Statistical Population Genomics pp. 191–230. New York, NY: Springer US pp. 191–230. 10.1007/978-1-0716-0199-0_9 31975169

[128] Anopheles gambiae 1000 Genomes Consortium and others (2017). Genetic diversity of the african malaria vector anopheles gambiae. Nature 552, 96. 29186111 10.1038/nature24995 PMC6026373

[129] Abernathey R.P., Augspurger T., Banihirwe A., Blackmon-Luca C.C., Crone T.J., Gentemann C.L., Hamman J.J., Henderson N., Lepore C., McCaie T.A. (2021). Cloud-native repositories for big scientific data. Computing in Science & Engineering 23, 26–35.

[130] Moore J., Allan C., Besson S., Burel J.M., Diel E., Gault D., Kozlowski K., Lindner D., Linkert M., Manz T. (2021). OME-NGFF: A next-generation file format for expanding bioimaging data-access strategies. Nature Methods 18, 1496–1498. 34845388 10.1038/s41592-021-01326-w PMC8648559

[131] Gowan T.A., Horel J.D., Jacques A.A., and Kovac A. (2022). Using cloud computing to analyze model output archived in Zarr format. Journal of Atmospheric and Oceanic Technology 39, 449–462.

[132] Dhapola P., Rodhe J., Olofzon R., Bonald T., Erlandsson E., Soneji S., and Karlsson G. (2022). Scarf enables a highly memory-efficient analysis of large-scale single-cell genomics data. Nature Communications 13, 4616. 10.1038/s41467-022-32097-3 PMC9360040 35941103

[133] Virshup I., Bredikhin D., Heumos L., Palla G., Sturm G., Gayoso A., Kats I., Koutrouli M., Berger B. (2023). The scverse project provides a computational ecosystem for single-cell omics data analysis. Nature Biotechnology 41, 604–606. 10.1038/s41587-023-01733-8 37037904

[134] Baker E.A., Huang M.Y., Lam A., Rahim M.K., Bieniosek M.F., Wang B., Zhang N.R., Mayer A.T., and Trevino A.E. (2023). emObject: domain specific data abstraction for spatial omics. bioRxiv. 2023.06.07.543950.

[135] Marconato L., Palla G., Yamauchi K.A., Virshup I., Heidari E., Treis T., Vierdag W.M., Toth M., Stockhaus S., Shrestha R.B. (2024). SpatialData: An open and universal data framework for spatial omics. Nature Methods 22, 58–62. 38509327 10.1038/s41592-024-02212-x PMC11725494

[136] Ruan X., Mueller M., Liu G., Görlitz F., Fu T.M., Milkie D.E., Lillvis J.L., Kuhn A., Gan Chong J., Hong J.L. (2024). Image processing tools for petabyte-scale light sheet microscopy data. Nature Methods 21, 2342–2352. 39420143 10.1038/s41592-024-02475-4 PMC11621031

[137] Köster J., and Rahmann S. (2012). Snakemake–a scalable bioinformatics workflow engine. Bioinformatics 28, 2520–2522. 22908215 10.1093/bioinformatics/bts480

[138] Katoh K., and Standley D.M. (2013). MAFFT multiple sequence alignment software version 7: Improvements in performance and usability. Molecular Biology and Evolution 30, 772–780. 23329690 10.1093/molbev/mst010 PMC3603318

[139] Turakhia Y., De Maio N., Thornlow B., Gozashti L., Lanfear R., Walker C.R., Hinrichs A.S., Fernandes J.D., Borges R., Slodkowicz G. (2020). Stability of SARS-CoV-2 phylogenies. PLOS Genetics 16, e1009175. 33206635 10.1371/journal.pgen.1009175 PMC7721162

[140] Saitou N., and Nei M. (1987). The neighbor-joining method: A new method for reconstructing phylogenetic trees. Molecular Biology and Evolution 4, 406–425. 3447015 10.1093/oxfordjournals.molbev.a040454

[141] Fitch W.M. (1971). Toward defining the course of evolution: Minimum change for a specific tree topology. Systematic Biology 20, 406–416.

[142] Hartigan J.A. (1973). Minimum mutation fits to a given tree. Biometrics 29, 53–65.

[143] Colquhoun R., Jackson B., O’Toole Á., and Rambaut A. (2023). SCORPIO: A utility for defining and classifying mutation constellations of virus genomes. Bioinformatics 39, btad575. 37713452 10.1093/bioinformatics/btad575 PMC10563142

[144] Otto S.P., Day T., Arino J., Colijn C., Dushoff J., Li M., Mechai S., Van Domselaar G., Wu J., Earn D.J. (2021). The origins and potential future of SARS-CoV-2 variants of concern in the evolving COVID-19 pandemic. Current Biology 31, R918–R929. 34314723 10.1016/j.cub.2021.06.049 PMC8220957

[145] Markov P.V., Ghafari M., Beer M., Lythgoe K., Simmonds P., Stilianakis N.I., and Katzourakis A. (2023). The evolution of SARS-CoV-2. Nature Reviews Microbiology 21, 361–379. 37020110 10.1038/s41579-023-00878-2

[146] European Centre for Disease Prevention and Control (2025). SARS-CoV-2 variants of concern as of 31 October 2025. https://www.ecdc.europa.eu/en/covid-19/variants-concern.

[147] Karthikeyan S., Levy J.I., De Hoff P., Humphrey G., Birmingham A., Jepsen K., Farmer S., Tubb H.M., Valles T., Tribelhorn C.E. (2022). Wastewater sequencing reveals early cryptic SARS-CoV-2 variant transmission. Nature 609, 101–108. 35798029 10.1038/s41586-022-05049-6 PMC9433318

[148] McBroome J., de Bernardi Schneider A., Roemer C., Wolfinger M.T., Hinrichs A.S., O’Toole A.N., Ruis C., Turakhia Y., Rambaut A., and Corbett-Detig R. (2024). A framework for automated scalable designation of viral pathogen lineages from genomic data. Nature Microbiology 9, 550–560. 10.1038/s41564-023-01587-5 PMC10847047 38316930

[149] De Maio N., Anoufa O., Smith K., Turakhia Y., and Goldman N. (2025). Highly recurrent multi-nucleotide mutations in SARS-CoV-2. Molecular Biology and Evolution pp. msaf272. 41134688 10.1093/molbev/msaf272 PMC12619124

[150] Grüning B., Dale R., Sjödin A., Chapman B.A., Rowe J., Tomkins-Tinch C.H., Valieris R., Köster J., and Team B. (2018). Bioconda: Sustainable and comprehensive software distribution for the life sciences. Nature Methods 15, 475–476. 29967506 10.1038/s41592-018-0046-7 PMC11070151

[151] Scornavacca C., Zickmann F., and Huson D.H. (2011). Tanglegrams for rooted phylogenetic trees and networks. Bioinformatics 27, i248–i256. 21685078 10.1093/bioinformatics/btr210 PMC3117342

[152] Duchene S., Featherstone L., Haritopoulou-Sinanidou M., Rambaut A., Lemey P., andBaele G. (2020). Temporal signal and the phylodynamic threshold of SARS-CoV-2. Virus Evolution 6, veaa061. 33235813 10.1093/ve/veaa061 PMC7454936

[153] Ghafari M., du Plessis L., Raghwani J., Bhatt S., Xu B., Pybus O.G., and Katzourakis A. (2022). Purifying selection determines the short-term time dependency of evolutionary rates in SARS-CoV-2 and pH1N1 influenza. Molecular Biology and Evolution 39, msac009. 35038728 10.1093/molbev/msac009 PMC8826518

[154] Garrison E., Kronenberg Z.N., Dawson E.T., Pedersen B.S., and Prins P. (2022). A spectrum of free software tools for processing the VCF variant call format: vcflib, bio-vcf, cyvcf2, hts-nim and slivar. PLOS Computational Biology 18, e1009123. 35639788 10.1371/journal.pcbi.1009123 PMC9286226

[155] Kluyver T., Ragan-Kelley B., Pérez F., Granger B., Bussonnier M., Frederic J., Kelley K., Hamrick J., Grout J., Corlay S., Ivanov P., Avila D., Abdalla S., and Willing C. (2016). Jupyter notebooks – a publishing format for reproducible computational workflows. In Loizides F., and Schmidt B., eds. Positioning and Power in Academic Publishing: Players, Agents and Agendas. IOS Press pp. 87 – 90.

[156] Cantoni D., Murray M.J., Kalemera M.D., Dicken S.J., Stejskal L., Brown G., Lytras S., Coey J.D., McKenna J., Bridgett S. (2022). Evolutionary remodelling of N-terminal domain loops fine-tunes SARS-CoV-2 spike. EMBO reports 23, e54322. 35999696 10.15252/embr.202154322 PMC9535765

[157] Tegally H., Moir M., Everatt J., Giovanetti M., Scheepers C., Wilkinson E., Subramoney K., Makatini Z., Moyo S., Amoako D.G. (2022). Emergence of SARS-CoV-2 Omicron lineages BA.4 and BA.5 in South Africa. Nature Medicine 28, 1785–1790. 10.1038/s41591-022-01911-2 PMC9499863 35760080

[158] Harvey W.T., Carabelli A.M., Jackson B., Gupta R.K., Thomson E.C., Harrison E.M., Ludden C., Reeve R., Rambaut A., Peacock S.J., Robertson D.L., and Consortium, C..G.U.C.U. (2021). SARS-CoV-2 variants, spike mutations and immune escape. Nature Reviews Microbiology 19, 409–424. 34075212 10.1038/s41579-021-00573-0 PMC8167834

[159] Akaishi T., Fujiwara K., and Ishii T. (2022). Insertion/deletion hotspots in the Nsp2, Nsp3, S1, and ORF8 genes of SARS-related coronaviruses. BMC Ecology and Evolution 22, 123. 36307763 10.1186/s12862-022-02078-7 PMC9616624

[160] silcn (2023). XBB.1*/BA.2.75*/XBB.1* recombinant with S:F486P (8 seq, Malaysia). https://github.com/cov-lineages/pango-designation/issues/1532.

[161] Groves N. (2021). Proposed new B.1 sublineage circulating in India. https://github.com/cov-lineages/pango-designation/issues/38.

[162] Pango designation team (2022). Scorpio constellation of B.1.617.1. https://github.com/cov-lineages/constellations/blob/main/constellations/definitions/cB.1.617.1.json.

[163] Pango designation team (2021). Scorpio constellation of B.1.617.2. https://github.com/cov-lineages/constellations/blob/main/constellations/definitions/cB.1.617.2.json.

