# Supplementary material for: A Pandemic-Scale Ancestral Recombination Graph for SARS-CoV-2
Source: bioRxiv. 2025 Nov 25:2023.06.08.544212. Preprint. [Version 3] doi: 10.1101/2023.06.08.544212 (PMC12642650; doi:10.1101/2023.06.08.544212)

```
Using a 434.8 megabyte ARG up to 2023-02-21, with 2482157 sampled SARS-CoV2 sequences
(317 trees, 2484587 mutations over 29904.0bp with 855 recomb. events)
```

44 main pango-X lineages	135 sub pango-X lineages
XA, XAA, XAC, XAD, XAE, XAF, XAG, XAJ, XAL, XAM, XAN, XAP, XAS, XAU, XAV, XAZ, XB, XBB, XBD, XBE, XBF, XBG, XBH, XBK, XBM, XBQ, XBR, XC, XE, XF, XG, XH, XJ, XL, XM, XN, XP, XQ, XR, XS, XU, XW, XY, XZ	XBB.1, XBB.1.1, XBB.1.11, XBB.1.13, XBB.1.14, XBB.1.15, XBB.1.18, XBB.1.18.1, XBB.1.22.1, XBB.1.28, XBB.1.29, XBB.1.3, XBB.1.30, XBB.1.32, XBB.1.34, XBB.1.35, XBB.1.37, XBB.1.4, XBB.1.4.1, XBB.1.43, XBB.1.43.1, XBB.1.45, XBB.1.45.1, XBB.1.46, XBB.1.5, XBB.1.5.1, XBB.1.5.10, XBB.1.5.100, XBB.1.5.102, XBB.1.5.104, XBB.1.5.107, XBB.1.5.11, XBB.1.5.12, XBB.1.5.13, XBB.1.5.14, XBB.1.5.15, XBB.1.5.16, XBB.1.5.17, XBB.1.5.18, XBB.1.5.19, XBB.1.5.2, XBB.1.5.20, XBB.1.5.21, XBB.1.5.23, XBB.1.5.24, XBB.1.5.25, XBB.1.5.26, XBB.1.5.28, XBB.1.5.3, XBB.1.5.30, XBB.1.5.31, XBB.1.5.32, XBB.1.5.33, XBB.1.5.34, XBB.1.5.35, XBB.1.5.36, XBB.1.5.37, XBB.1.5.38, XBB.1.5.39, XBB.1.5.4, XBB.1.5.40, XBB.1.5.41, XBB.1.5.43, XBB.1.5.46, XBB.1.5.47, XBB.1.5.48, XBB.1.5.49, XBB.1.5.5, XBB.1.5.51, XBB.1.5.52, XBB.1.5.55, XBB.1.5.56, XBB.1.5.57, XBB.1.5.59, XBB.1.5.6, XBB.1.5.60, XBB.1.5.61, XBB.1.5.62, XBB.1.5.63, XBB.1.5.64, XBB.1.5.65, XBB.1.5.66, XBB.1.5.67, XBB.1.5.69, XBB.1.5.7, XBB.1.5.73, XBB.1.5.75, XBB.1.5.77, XBB.1.5.78, XBB.1.5.79, XBB.1.5.8, XBB.1.5.80, XBB.1.5.86, XBB.1.5.88, XBB.1.5.9, XBB.1.5.90, XBB.1.5.91, XBB.1.5.93, XBB.1.5.95, XBB.1.5.96, XBB.1.5.97, XBB.1.7, XBB.1.9, XBB.1.9.1, XBB.1.9.2, XBB.1.9.4, XBB.2, XBB.2.1, XBB.2.10, XBB.2.11.1, XBB.2.2, XBB.2.4, XBB.2.5, XBB.2.6, XBB.2.7, XBB.2.8, XBB.3, XBB.3.1, XBB.3.2, XBB.3.5, XBB.4, XBB.6, XBB.6.1, XBB.8, XBB.9, XBF.1, XBF.10, XBF.2, XBF.3, XBF.4, XBF.5, XBF.6, XBF.7, XBF.9, XBK.1

```
Consensus mutations for each lineage taken from https://covidcg.org
```

Bold = main pango

RE node	*pango*	*parents*	*break@*	*# descendants*	*Most common*
**122444**	**XA**	B.1.177.18/B.1.1.7	21765	39 of which 39 XA	XA: 39
	~~XB~~			0 of which 0 XB	
**414488**	**XC**	AY.29/B.1.1.7	27390	5 of which 5 XC	XC: 5
	XD	not in dataset			
**965353**	**XE**	BA.1.17.2/BA.2	11283	1156 of which 1116 XE	XE: 1116, BA.2: 37, XH: 2
**946761**	**XF**	AY.4/BA.1	6402	16 of which 16 XF	XF: 16
**1083412**	**XG**	BA.1.17/BA.2	6513	3 of which 3 XG	XG: 3
965353	XH	BA.1.17.2/BA.2	11283	1156 of which 2 XH	XE: 1116, BA.2: 37, XH: 2
**966905**	**XJ**	BA.1.17.2/BA.2	17410	85 of which 68 XJ	XJ: 68, BA.2: 17
	XK	not in dataset			
**1034619**	**XL**	BA.1.17.2/BA.2	8393	64 of which 64 XL	XL: 64
	~~XM~~			0 of which 0 XM	
	~~XN~~			0 of which 0 XN	
	~~XP~~			0 of which 0 XP	
**1058654**	**XQ**	BA.1.1.15/BA.2.9	5386	154 of which 55 XQ	XQ: 55, BA.2: 37, XAM: 21
1058654	XR	BA.1.1.15/BA.2.9	5386	154 of which 17 XR	XQ: 55, BA.2: 37, XAM: 21
**1000242**	**XS**	AY.103/BA.1.1	10449	17 of which 17 XS	XS: 17
	XT	not in dataset			
1058654	XU	BA.1.1.15/BA.2.9	5386	154 of which 1 XU	XQ: 55, BA.2: 37, XAM: 21
	XV	not in dataset			
**1159411**	**XW**	BA.1.1.15/BA.2	4321	32 of which 32 XW	XW: 32
**1187989**	**XY**	BA.1.1/BA.2	12880	23 of which 23 XY	XY: 23
**964555**	**XZ**	BA.2/BA.1.17.2	26060	253 of which 48 XZ	BA.2: 156, XZ: 48, XAP: 20
1058654	XAA	BA.1.1.15/BA.2.9	5386	154 of which 17 XAA	XQ: 55, BA.2: 37, XAM: 21
	XAB	not in dataset			
964555	XAC	BA.2/BA.1.17.2	26060	253 of which 18 XAC	BA.2: 156, XZ: 48, XAP: 20
964555	XAD	BA.2/BA.1.17.2	26060	253 of which 2 XAD	BA.2: 156, XZ: 48, XAP: 20
964555	XAE	BA.2/BA.1.17.2	26060	253 of which 9 XAE	BA.2: 156, XZ: 48, XAP: 20
**1177107**	**XAF**	BA.1.1/BA.2	10447	36 of which 1 XAF	BA.2: 35, XAF: 1
1058654	XAG	BA.1.1.15/BA.2.9	5386	154 of which 6 XAG	XQ: 55, BA.2: 37, XAM: 21
	XAH	not in dataset			
	~~XAJ~~			0 of which 0 XAJ	
	XAK	not in dataset			
1003220	XAL	BA.1.1/BA.2	21595	45 of which 3 XAL	XM: 26, BA.2: 16, XAL: 3
1058654	XAM	BA.1.1.15/BA.2.9	5386	154 of which 21 XAM	XQ: 55, BA.2: 37, XAM: 21
	~~XAN~~			167127 of which 7 XAN	BA.5.2.1: 30582, BA.5.1: 18649, BA.5.2: 15522
964555	XAP	BA.2/BA.1.17.2	26060	253 of which 20 XAP	BA.2: 156, XZ: 48, XAP: 20
	XAQ	not in dataset			
	XAR	not in dataset			
	~~XAS~~			0 of which 0 XAS	
	XAT	not in dataset			
	~~XAU~~			0 of which 0 XAU	
	~~XAV~~			167127 of which 13 XAV	BA.5.2.1: 30582, BA.5.1: 18649, BA.5.2: 15522
	XAW	not in dataset			
	XAY	not in dataset			
	~~XAZ~~			167127 of which 133 XAZ	BA.5.2.1: 30582, BA.5.1: 18649, BA.5.2: 15522
	XBA	not in dataset			
1396207	XBB	BA.2.10/BM.1.1.1	22577	6455 of which 71 XBB	XBB.1.5: 3338, XBB.1: 524, XBB.1.5.7: 224
	XBC	not in dataset			
**1378208**	**XBD**	BA.2.75.2/BA.5.2.1	24620	30 of which 30 XBD	XBD: 30
	~~XBE~~			167127 of which 65 XBE	BA.5.2.1: 30582, BA.5.1: 18649, BA.5.2: 15522
**1420385**	**XBF**	BA.5.2.1/CJ.1	9866	185 of which 124 XBF	XBF: 124, XBF.4: 22, XBF.3: 22
**1291970**	**XBG**	BA.2.76/BA.5.2	22917	25 of which 25 XBG	XBG: 25
**1379419**	**XBH**	BA.2.1/BA.2.75.2	22001	6 of which 2 XBH	BA.2: 4, XBH: 2
	~~XBK~~			0 of which 0 XBK	
**1348822**	**XBM**	BA.2.76/BF.3	22917	12 of which 10 XBM	XBM: 10, BF.3: 2
	~~XBQ~~			0 of which 0 XBQ	
**1420166**	**XBR**	BN.3.1/BQ.1.25	22190	1 of which 1 XBR	XBR: 1

```
21 total pango X recombinant origins of which 19 include all descendants of the dominant gro
up (exceptions: XM and XBB)
```

Pango-X Subgraphs

Below we display subgraphs for all the main PangoX lineages that have samples present in the *sc2ts* ARG. Pango designations for both samples and internal nodes were assigned using XX TODO: fill out details XXX. For nodes with large numbers of descendants, only a selected sample of (say) 20-50 Pango X samples are shown. Extra descendants of a node are shown with dotted lines indicating additional immediate children of a node. In some cases, additional descendant nodes of different Pango designations (e.g. BA.2) are shown for context.

Recombination nodes are presented as larger circular nodes, with a Pango designation followed by the breakpoint position(s) surrounded by slashes, e.g. a breakpoint at position 1234 bp is indicated as **/1234/** (but note that PangoX lineages that are not of recombinant origin in sc2ts will not have a clear recombination node). Mutations within each subgraph (tickmarks along edges) are coloured pink if they are flagged as consensus mutations for those lineages: often such mutations occur in lineages above the PangoX origination node. Alternatively, if there are multiple mutations at the same site within a subgraph (indicating reversions or recurrent mutations) they plotted in a unique colour. For example, two green mutation tickmarks will represent mutations at the same site. If one is a reversion of a previous mutation (often indicating an unparsimonious reconstruction of topology), then the mutation is emphasised with a solid black outline. Deletion mutations are filled in black, and reversions of deletions (expected not to happen spontaneously) are magenta with a black outline.

In the PDF version of this document, hovering over node names will reveal the sample_id of a node, and hovering over a mutation will reveal the position of the mutation and the inherited vs derived state. E.g. a mouseover label of `mut:A1234T` denotes a mutation from an A to a T at position 1234 in the genome. Technially this is implemented by faking a URL (this leads to the slightly annoying behaviour that actually clicking on the hover-over text will attempt to open a non-existent URL).

Subgraph of pango XA: (39 samples, 39 shown)

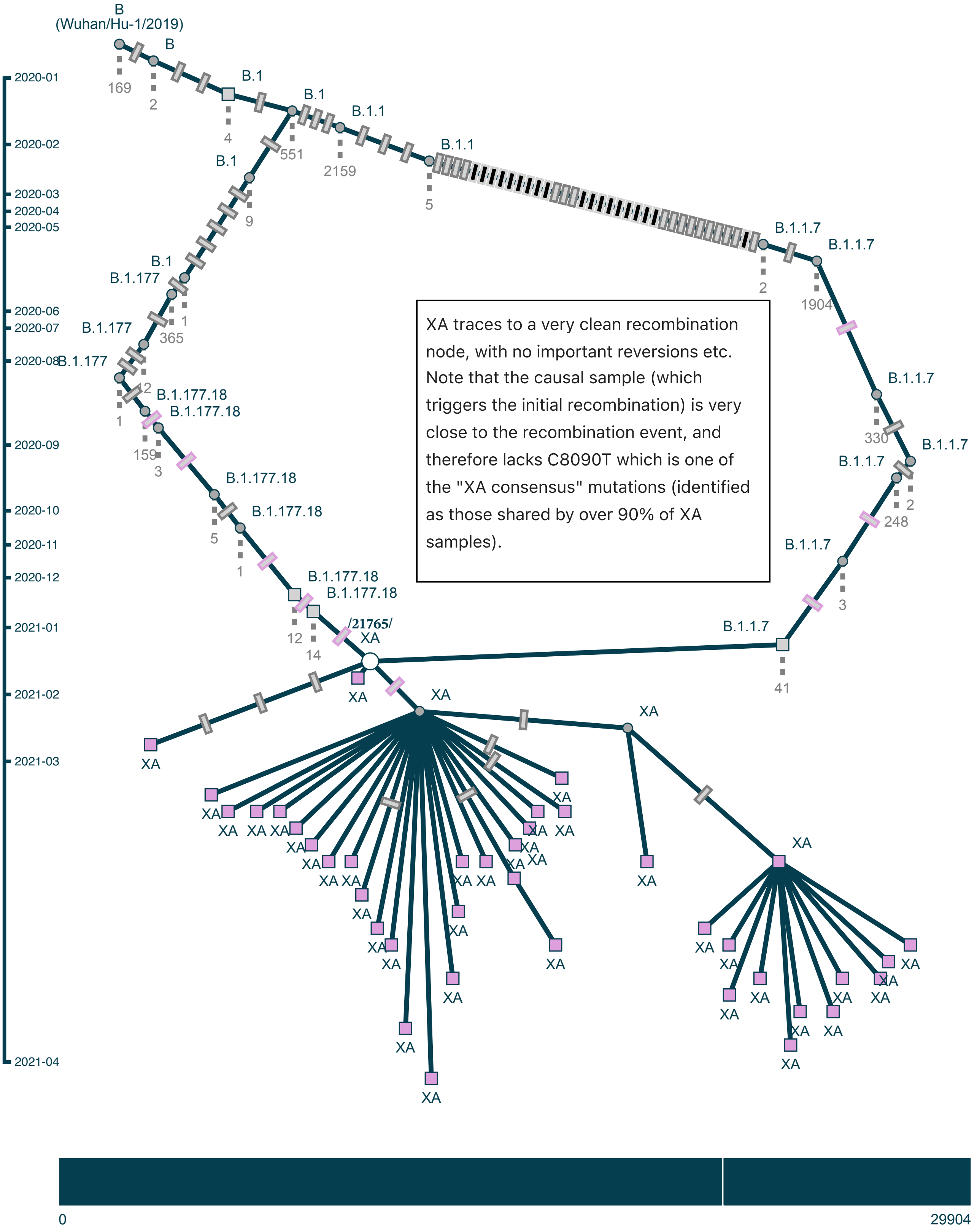

Subgraph of pango XB: (192 samples, 22 shown)

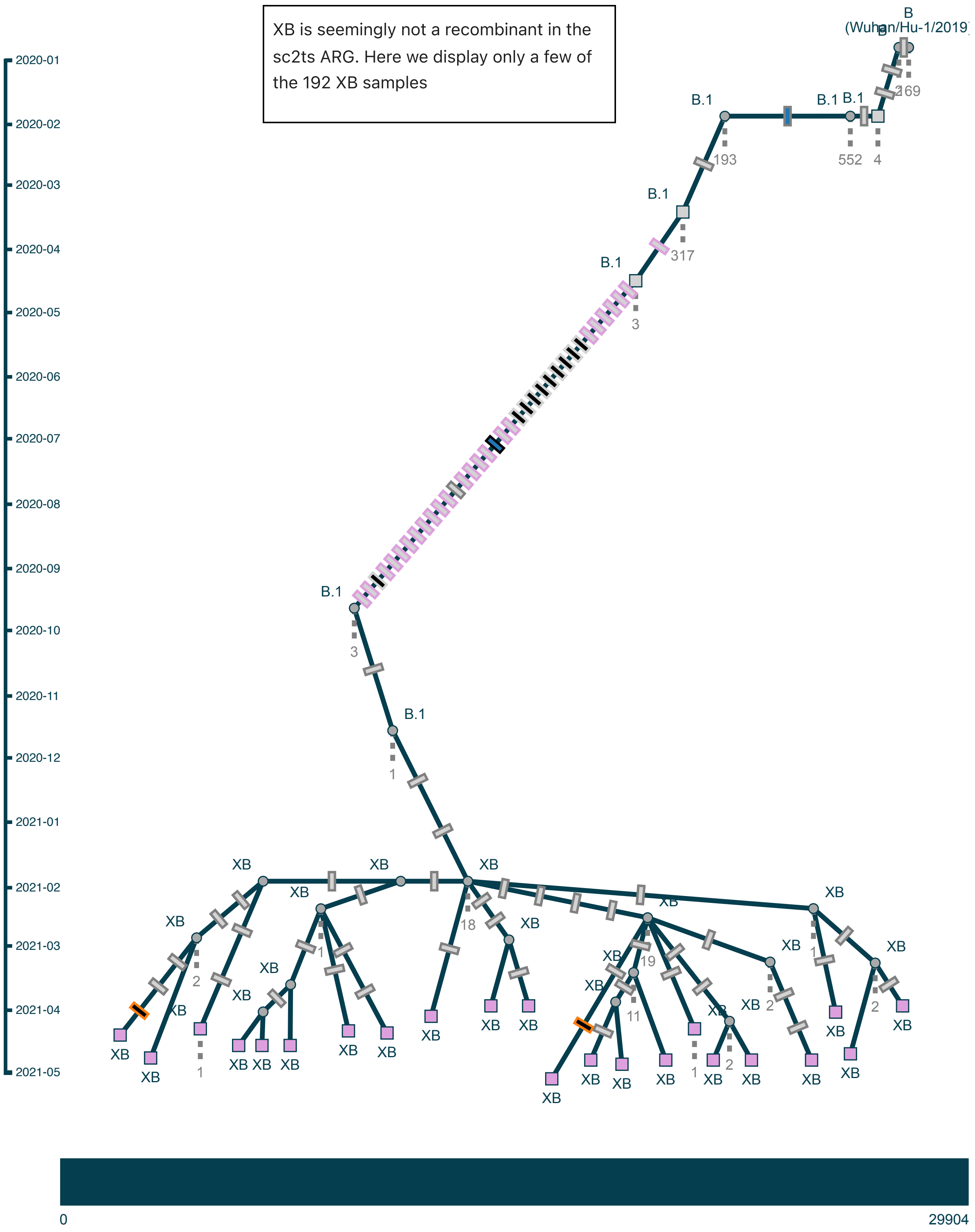

Subgraph of pango XC: (5 samples, 5 shown)

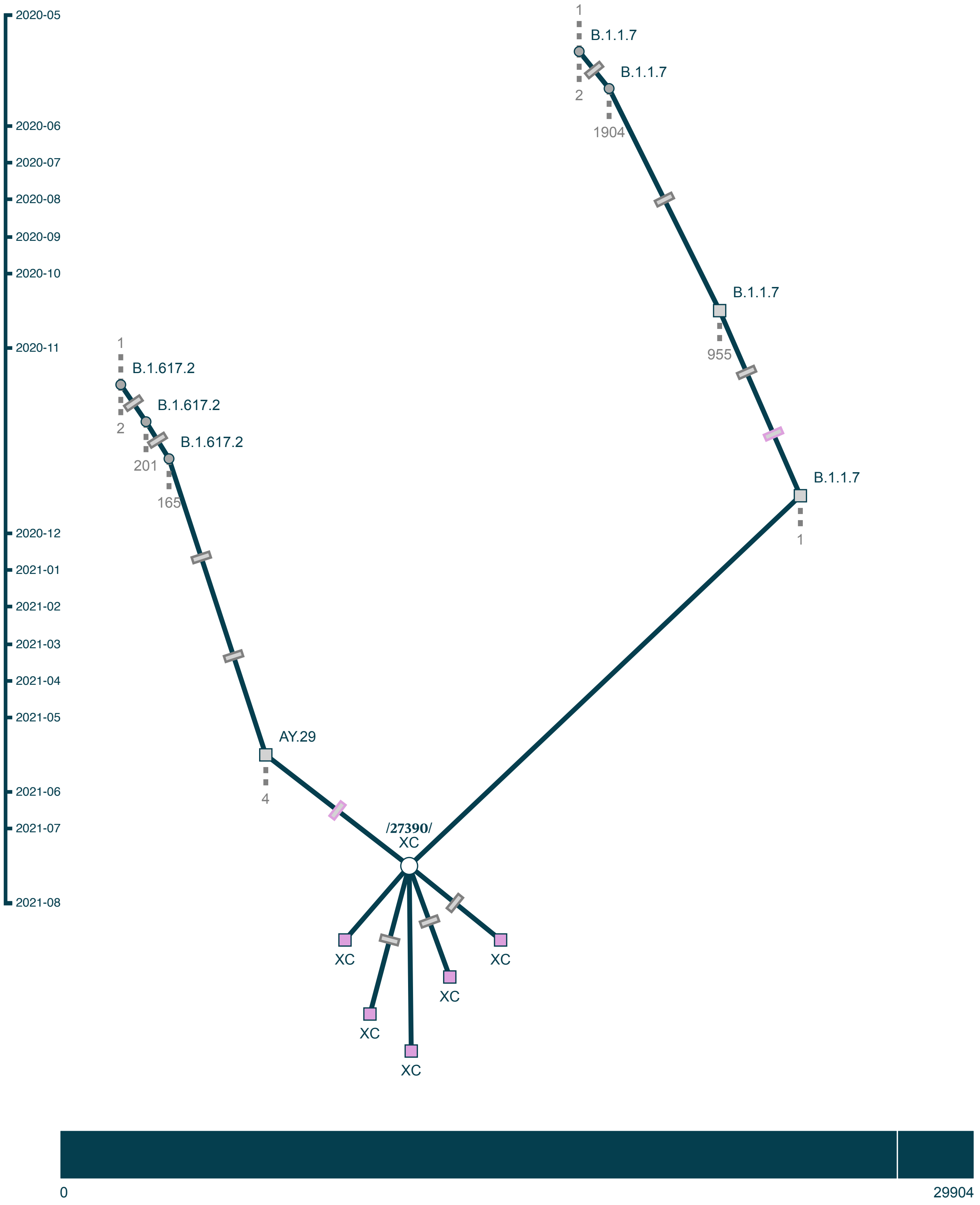

Subgraph of pango XE/XH: (1118 samples, 22 shown)

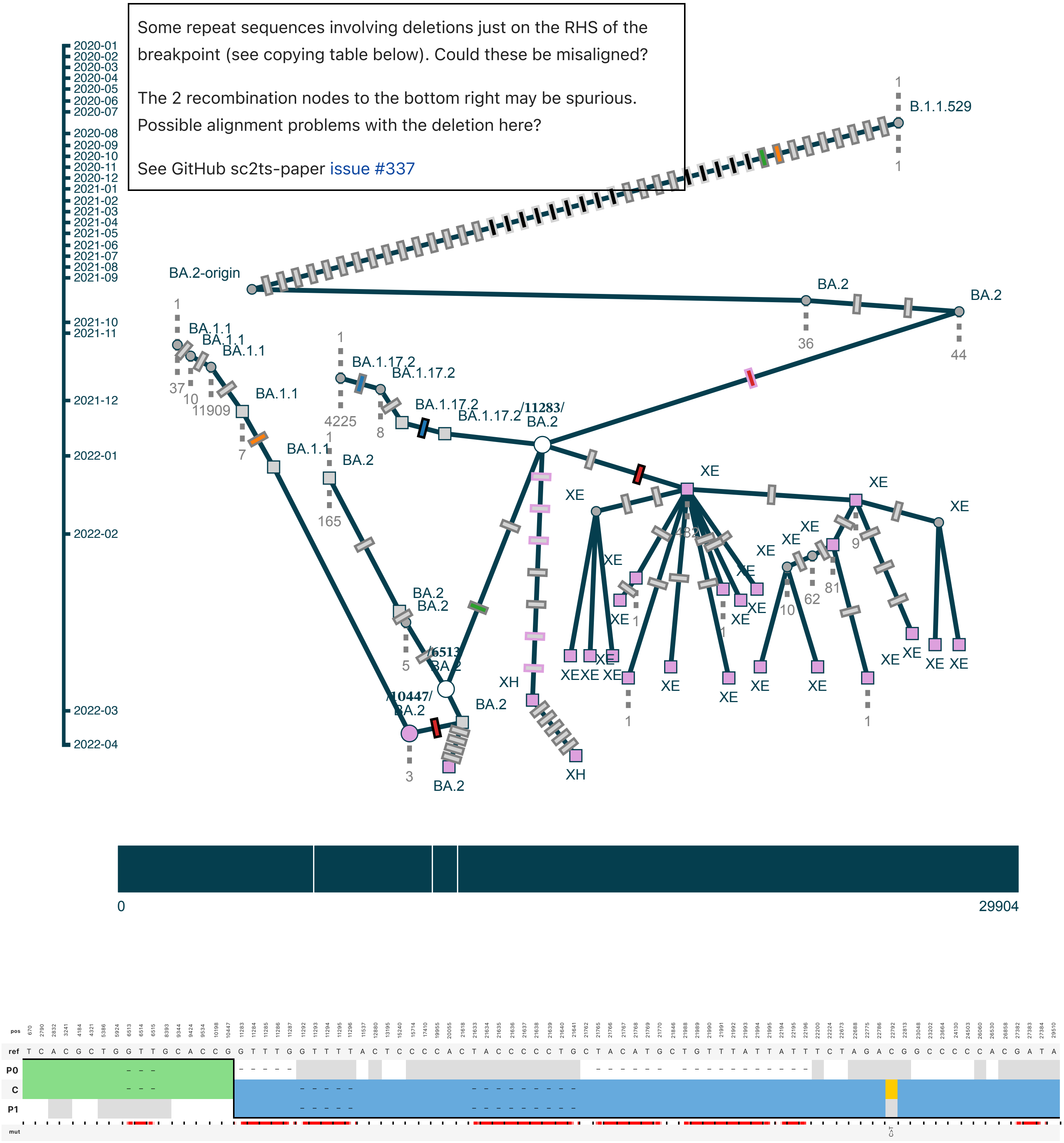

Subgraph of pango XF: (16 samples, 16 shown)

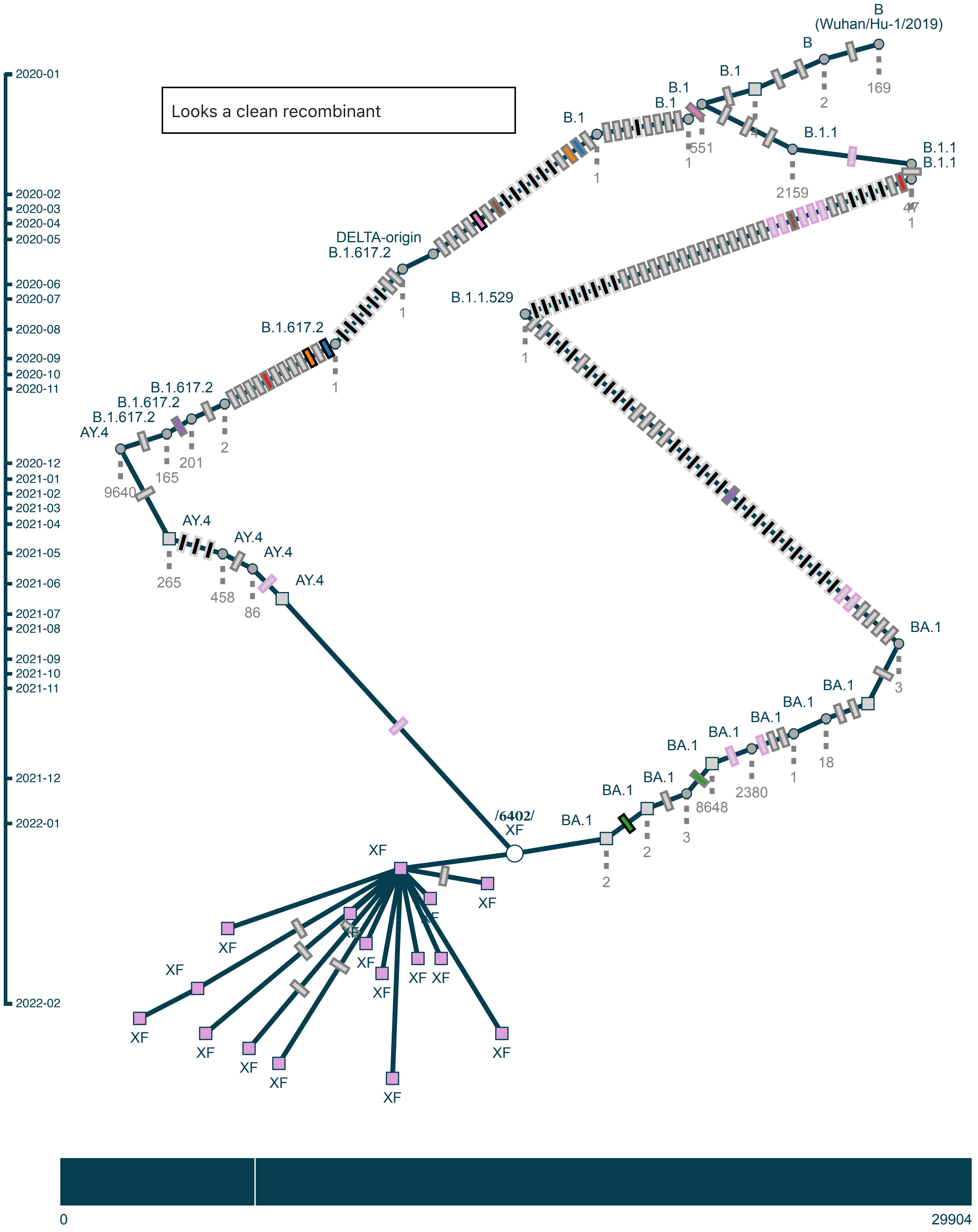

Subgraph of pango XG: (3 samples, 3 shown)

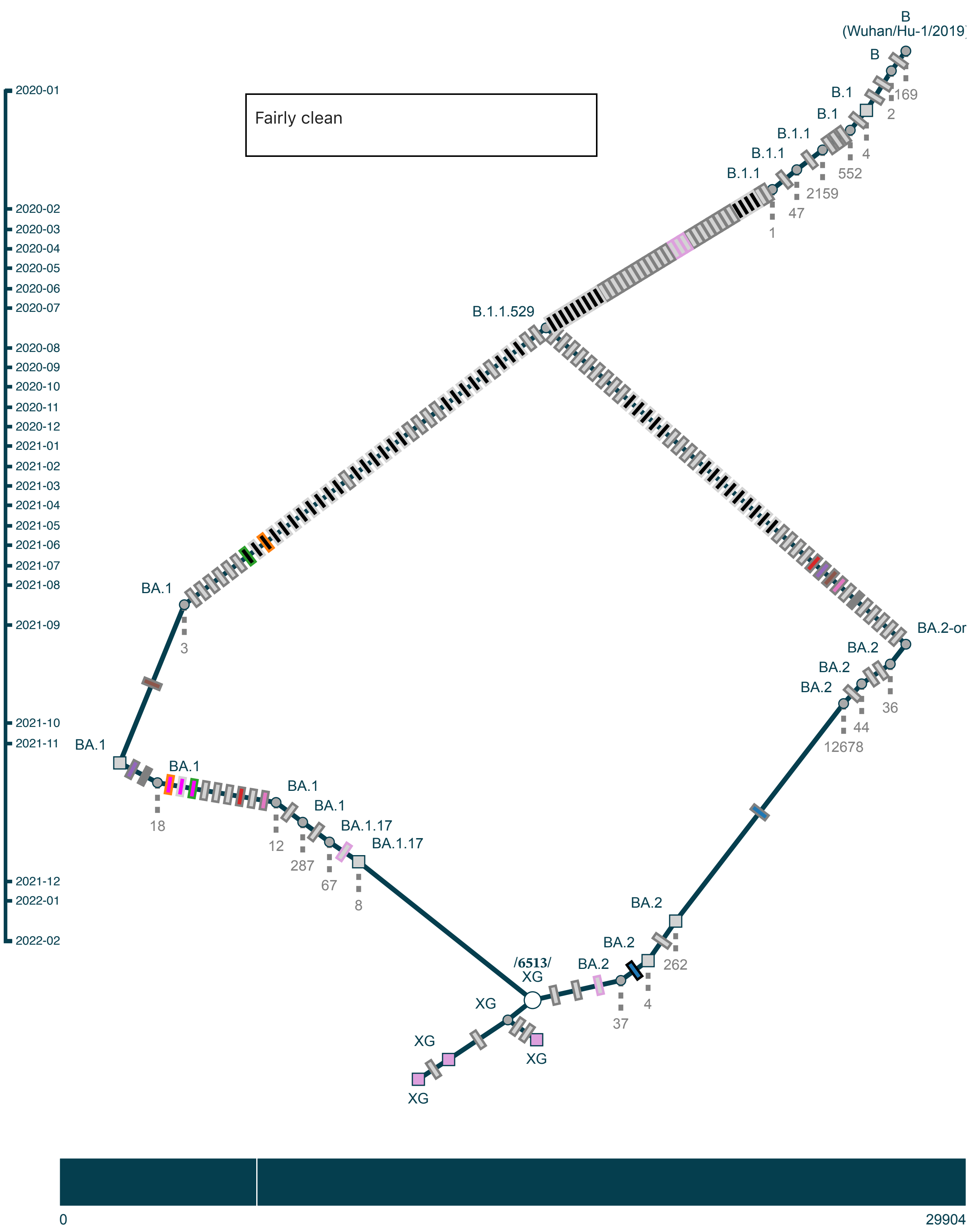

Subgraph of pango XJ: (68 samples, 68 shown)

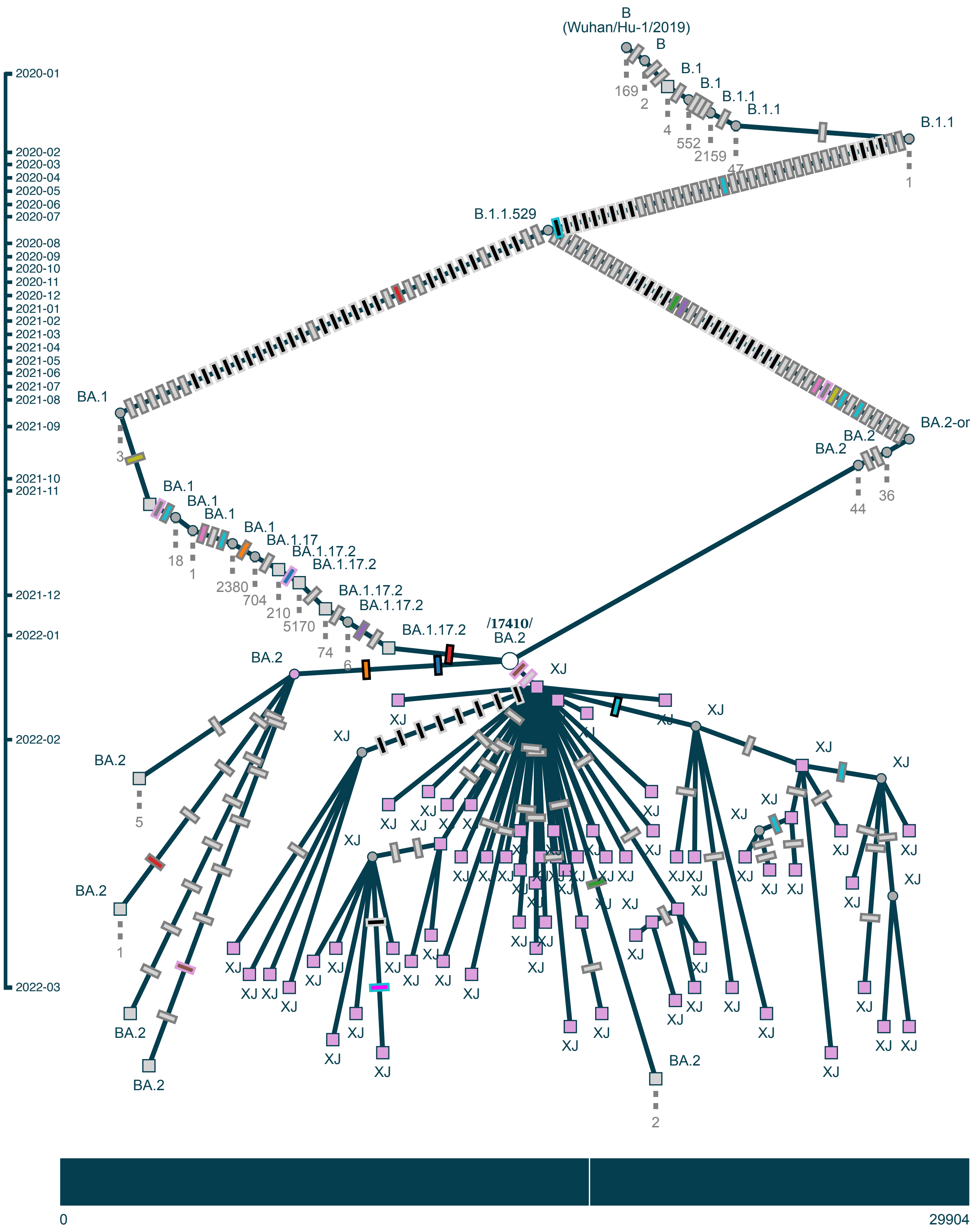

Subgraph of pango XL: (64 samples, 64 shown)

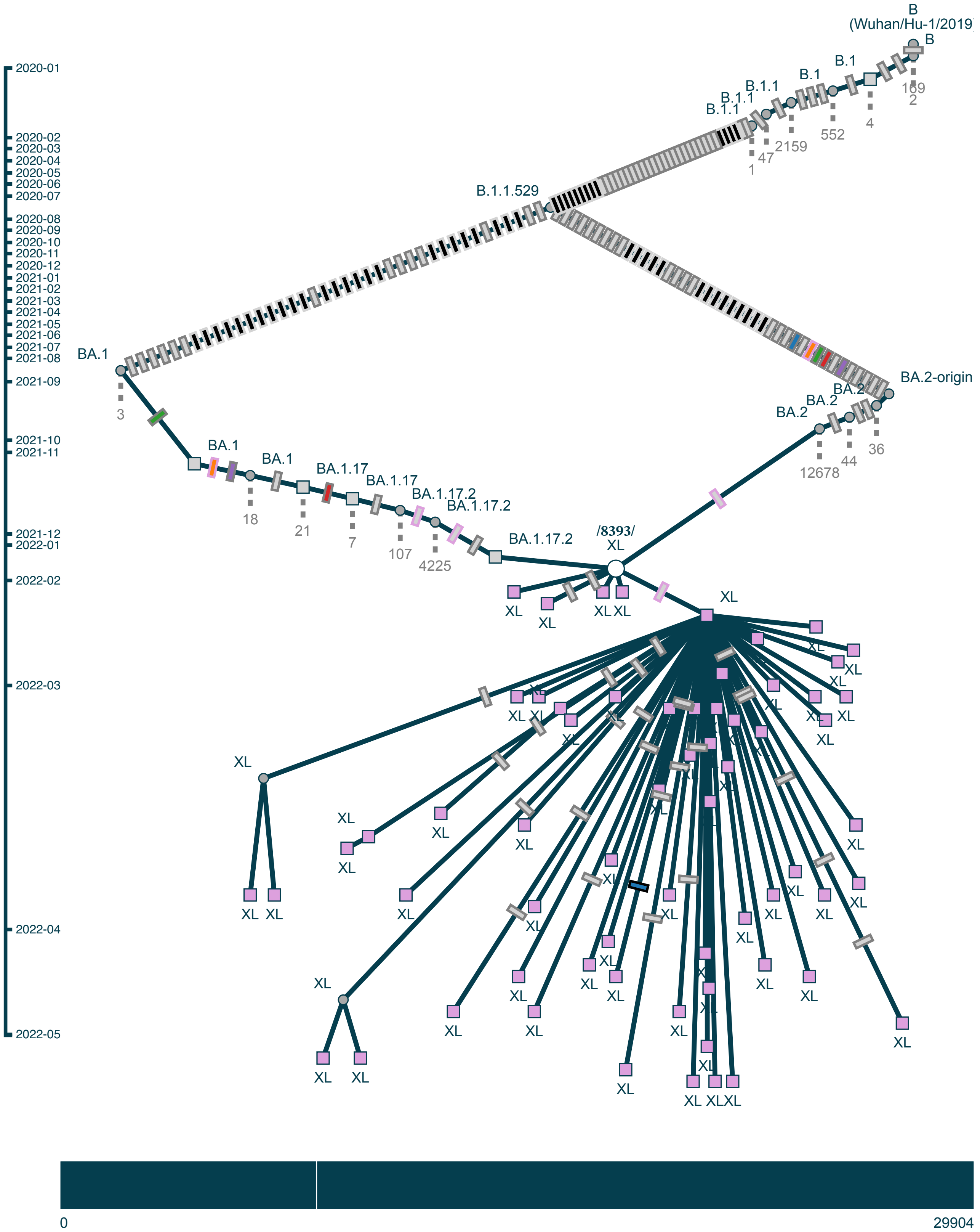

Subgraph of pango XM/XAL: (32 samples, 32 shown)

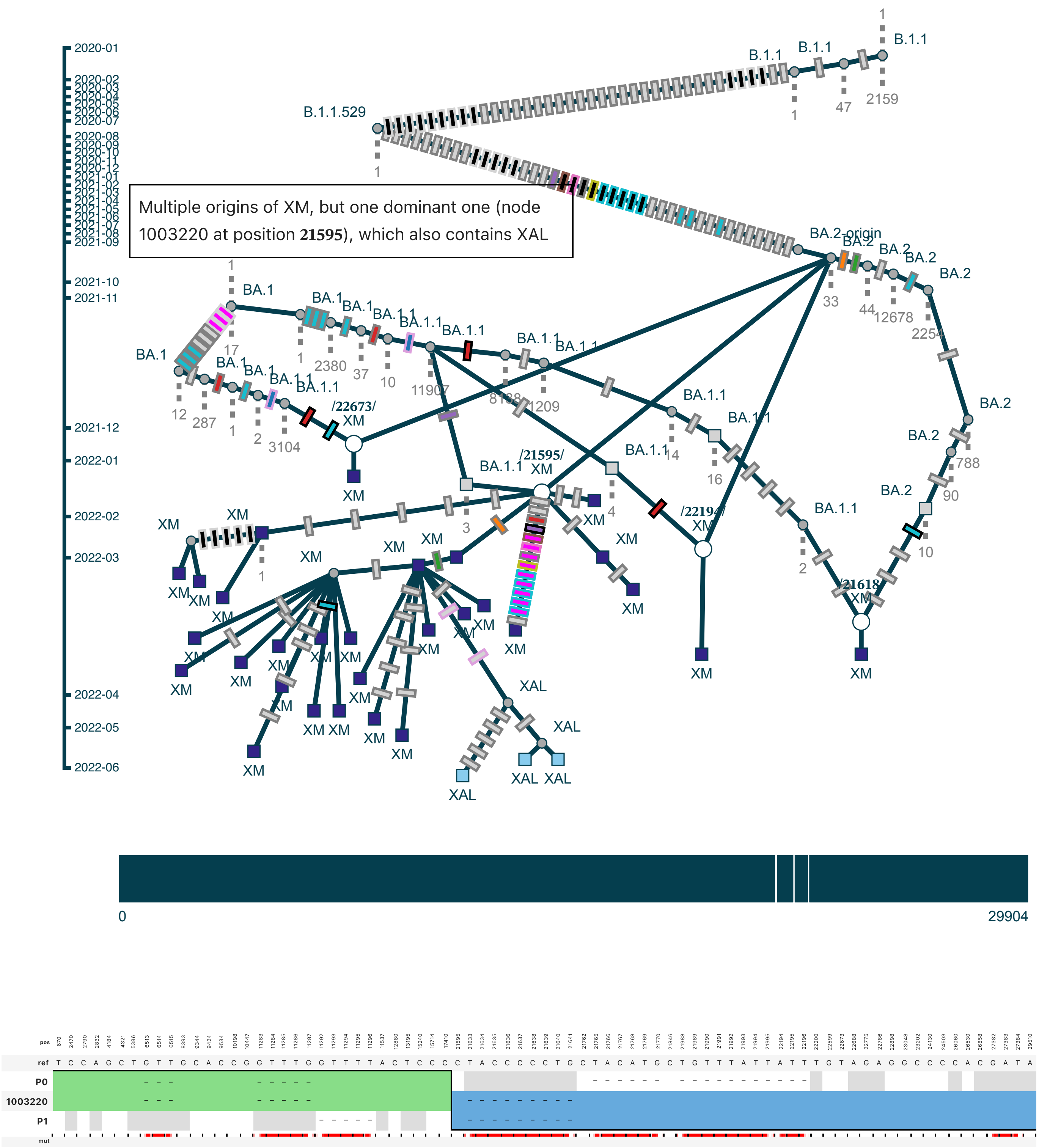

Subgraph of pango XN/XAU: (128 samples, 128 shown)

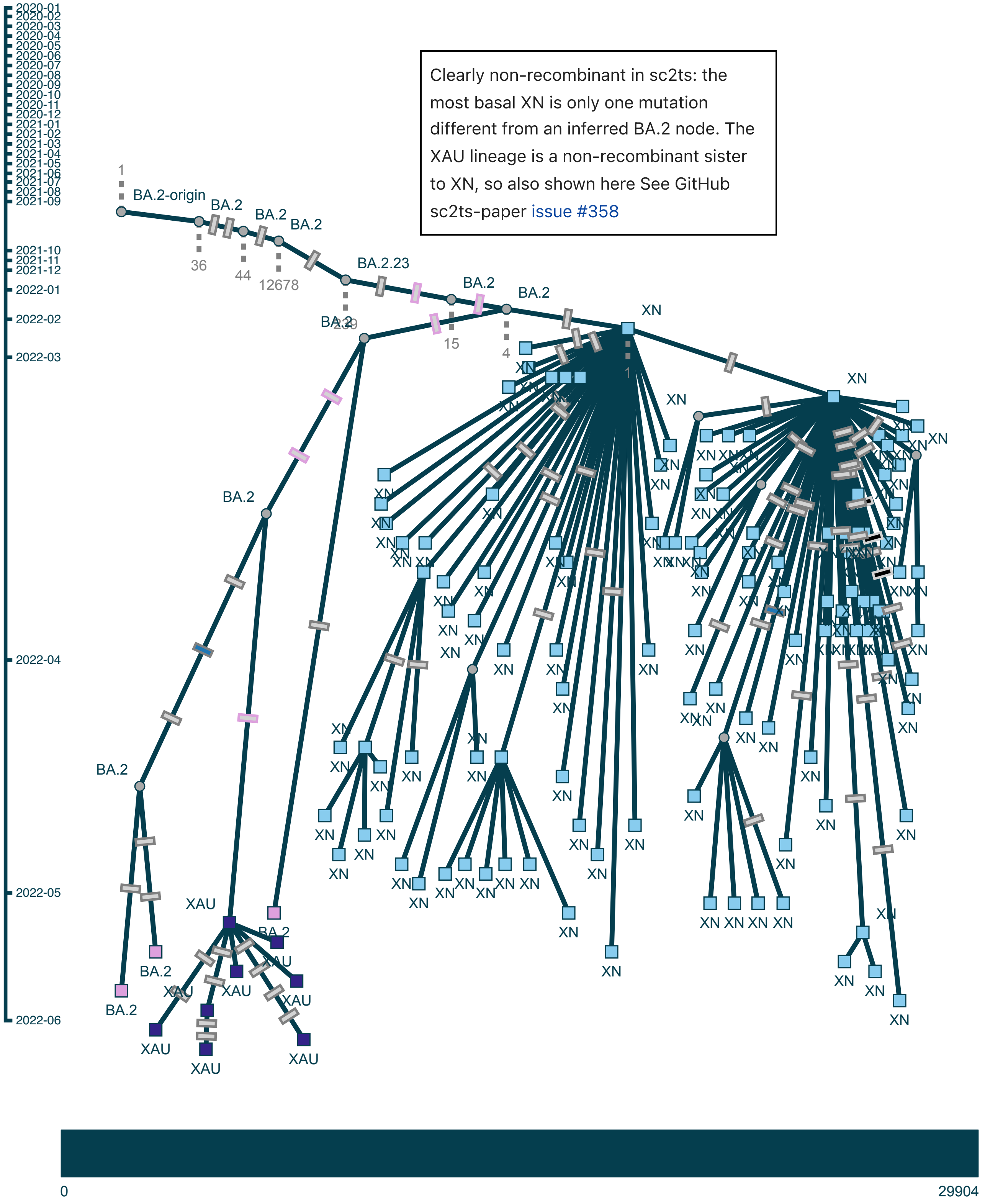

Subgraph of pango XP: (45 samples, 45 shown)

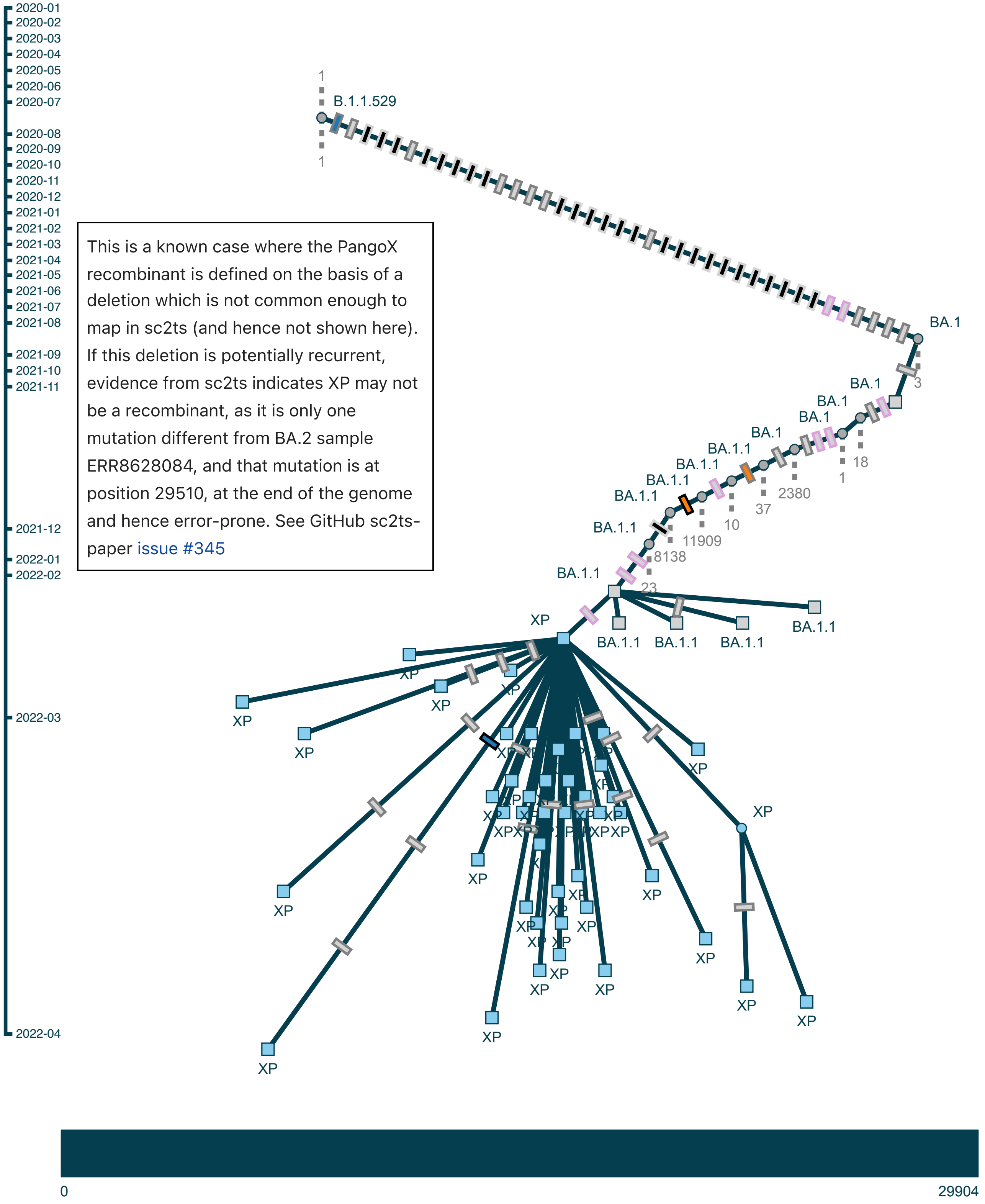

Subgraph of pango XQ/XR/XU/XAA/XAG/XAM: (117 samples, 49 shown)

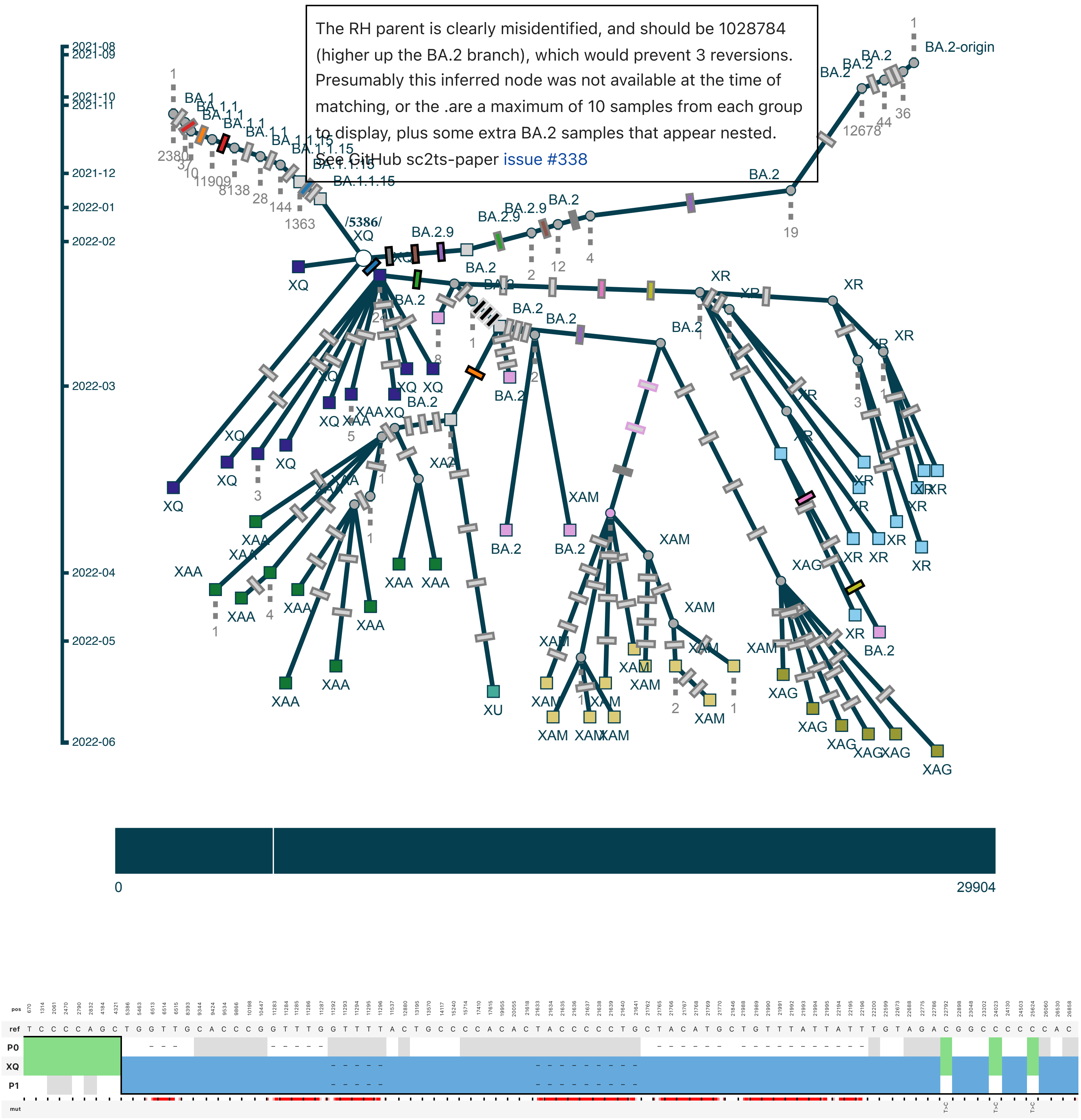

Subgraph of pango XS: (17 samples, 17 shown)

Although there are two recombination nodes adjacent to each other. The second node is likely to be an artifact See GitHub sc2ts-paper issue #287

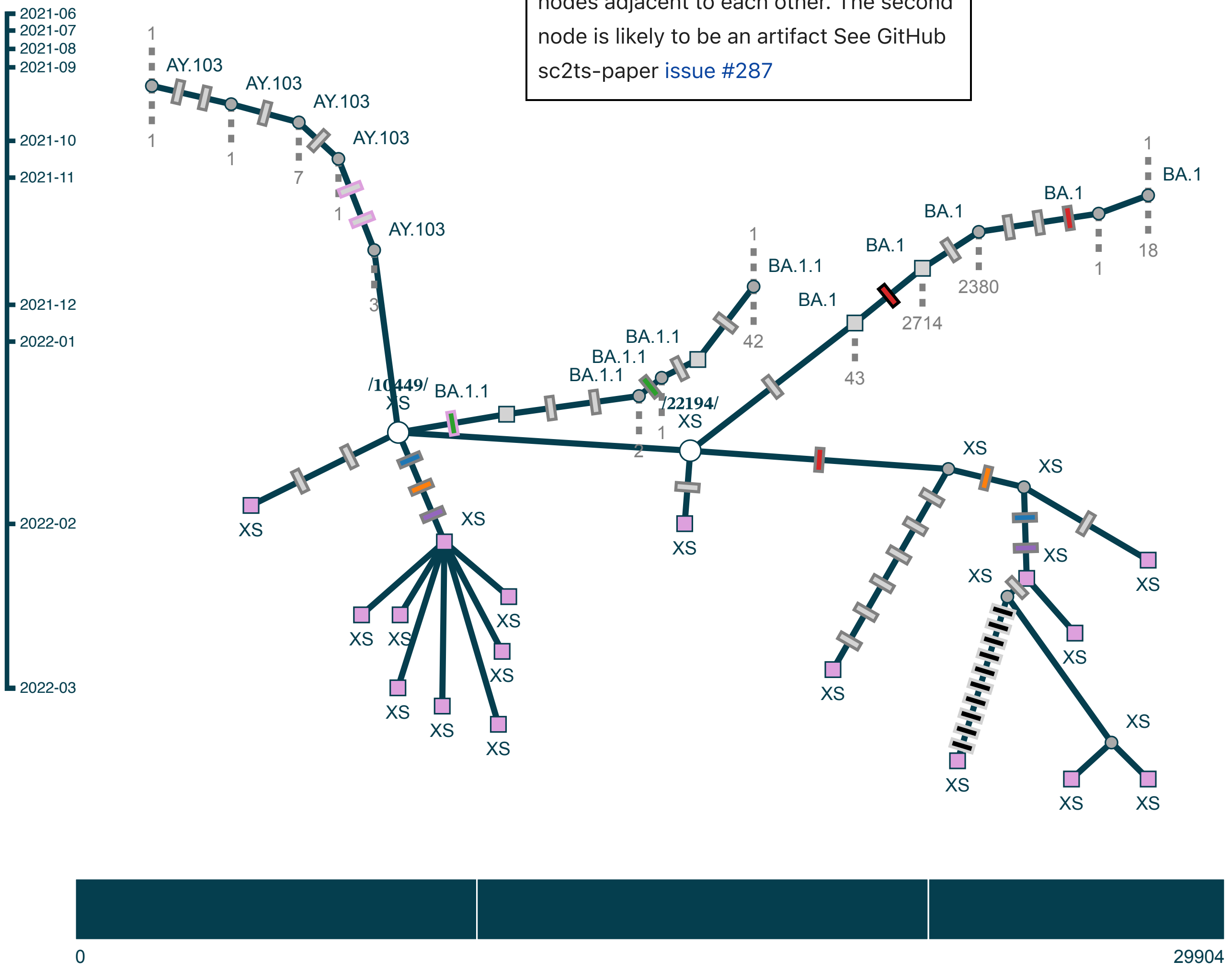

Subgraph of pango XW: (32 samples, 32 shown)

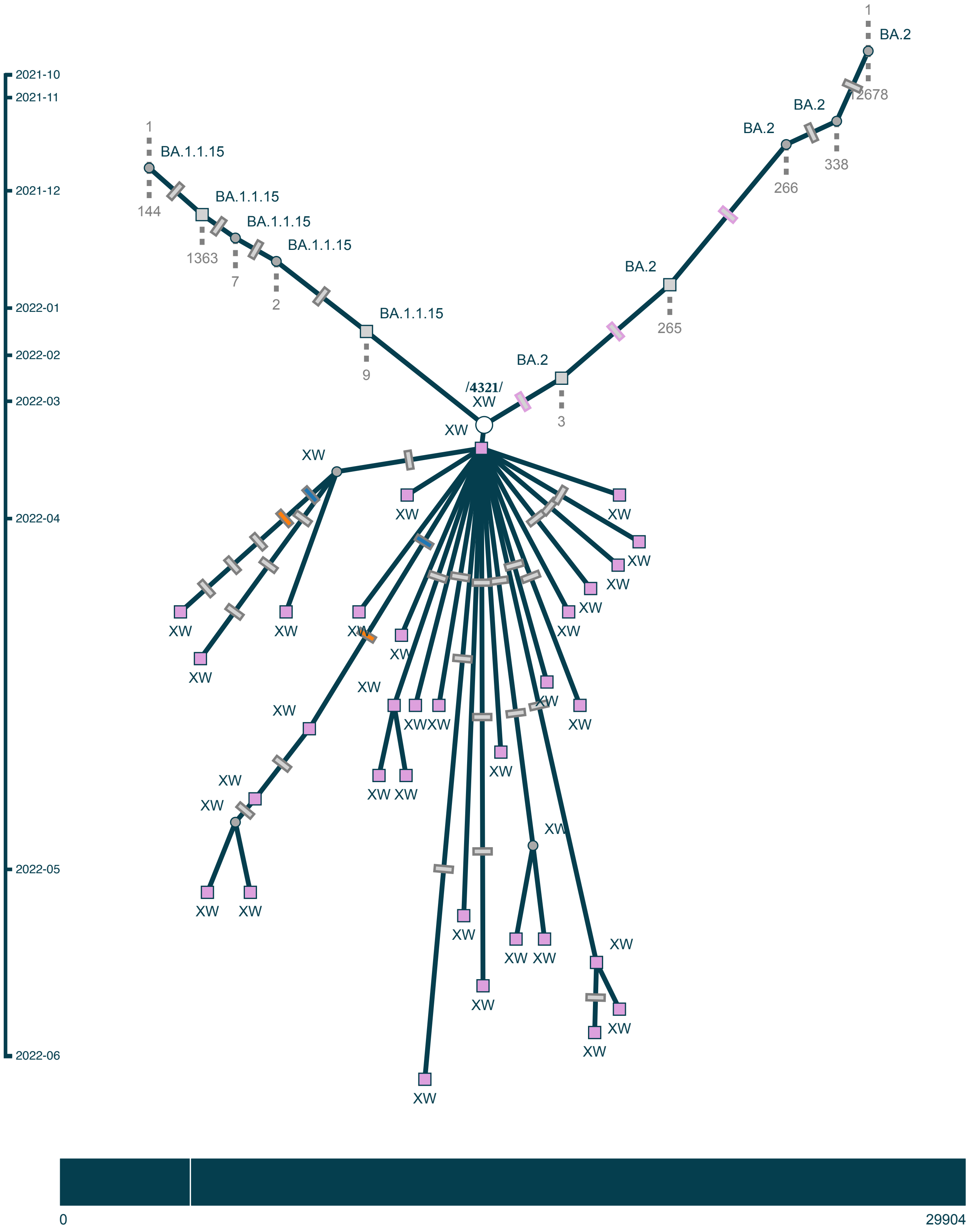

Subgraph of pango XY: (23 samples, 23 shown)

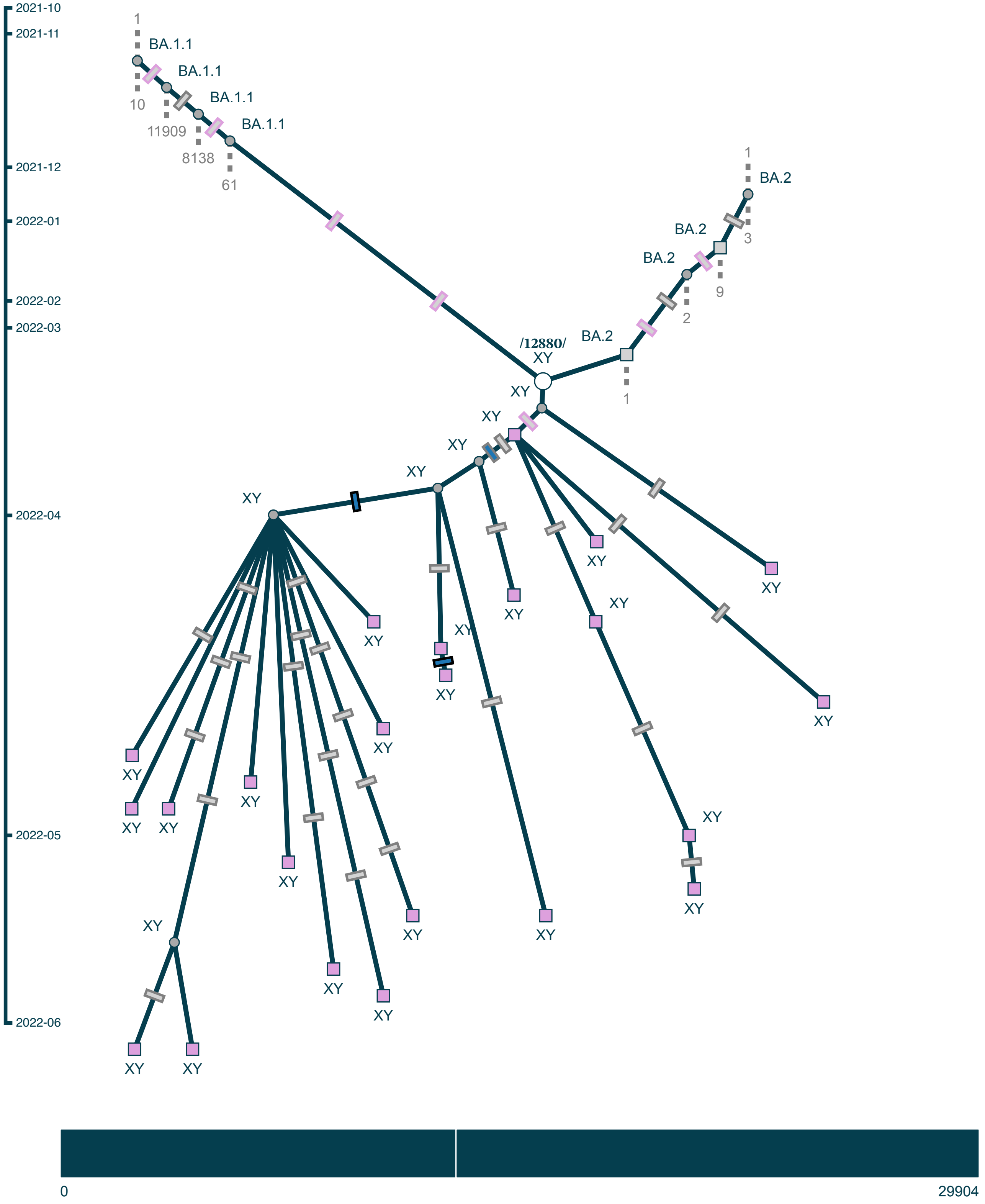

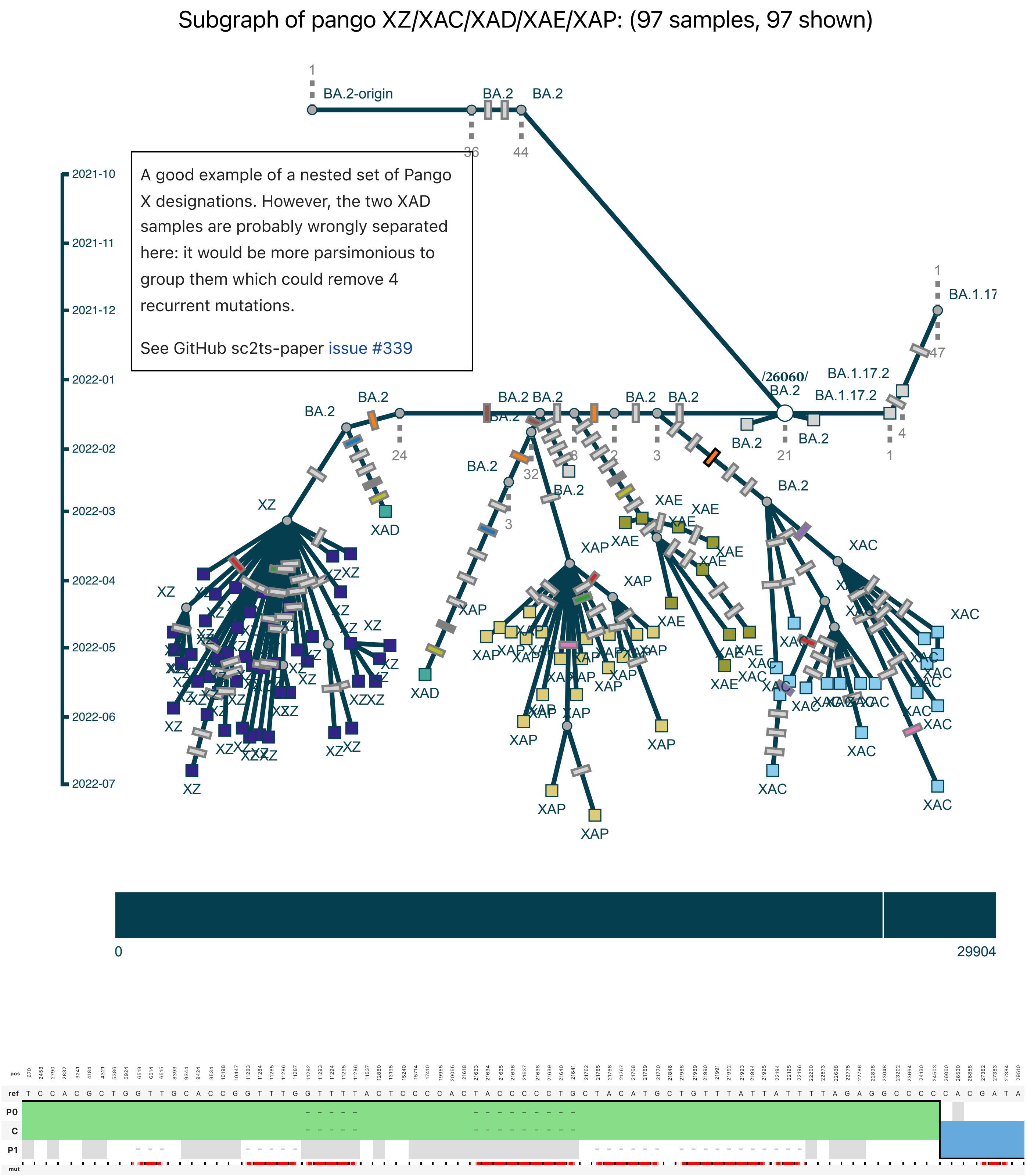
Subgraph of pango XZ/XAC/XAD/XAE/XAP: (97 samples, 97 shown)
A good example of a nested set of Pango X designations. However, the two XAD samples are probably wrongly separated here: it would be more parsimonious to group them which could remove 4 recurrent mutations.
See GitHub sc2ts-paper issue #339
BA.2-origin
BA.2
BA.1.17
BA.1.17.2
/26060/
XZ
XAD
XAP
XAE
XAC
2021-10
2021-11
2021-12
2022-01
2022-02
2022-03
2022-04
2022-05
2022-06
2022-07
0
29904
pos
ref
P0
C
P1
mut

Subgraph of pango XAF: (1 sample, 1 shown)

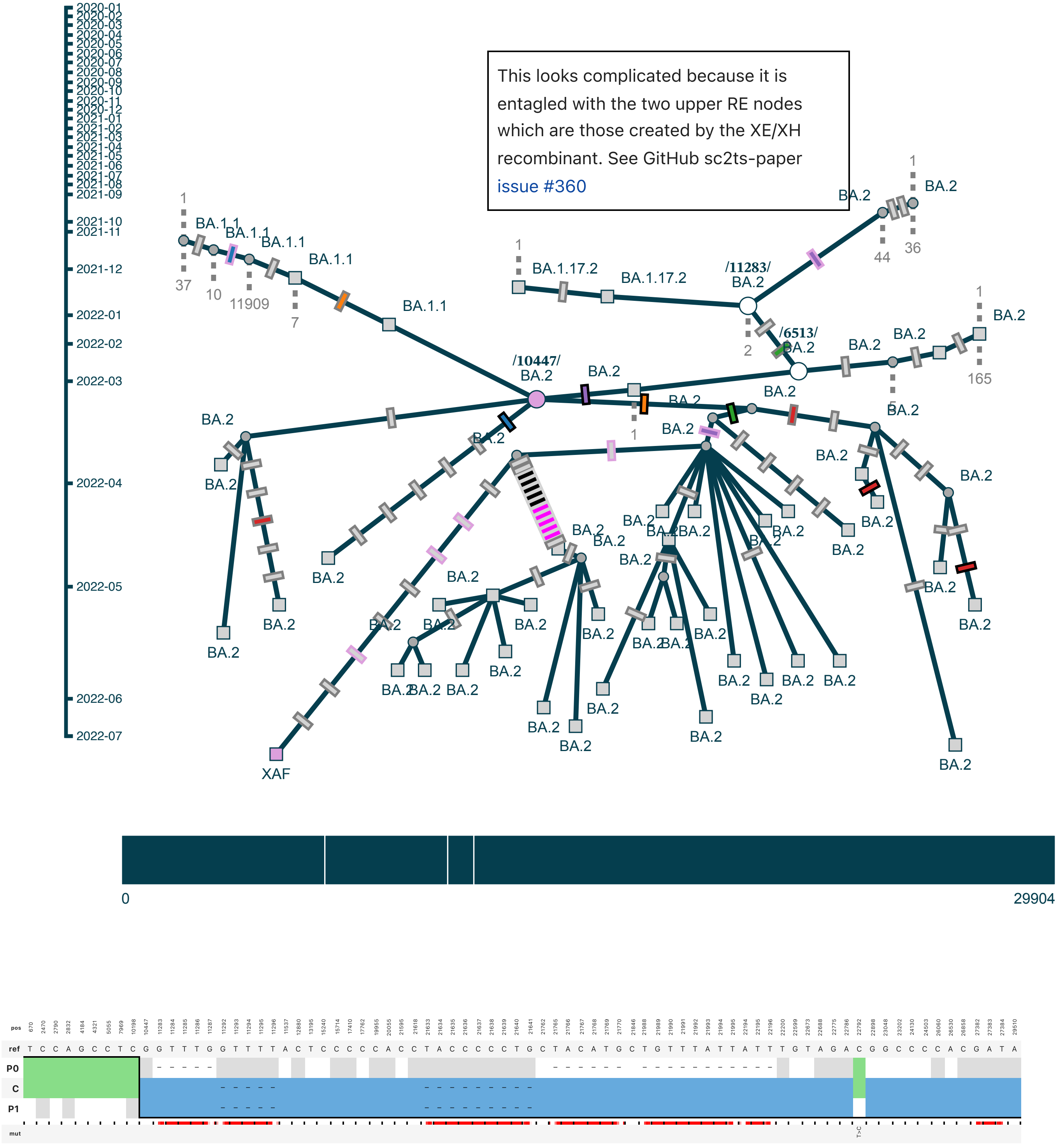

Subgraph of pango XAJ: (18 samples, 18 shown)

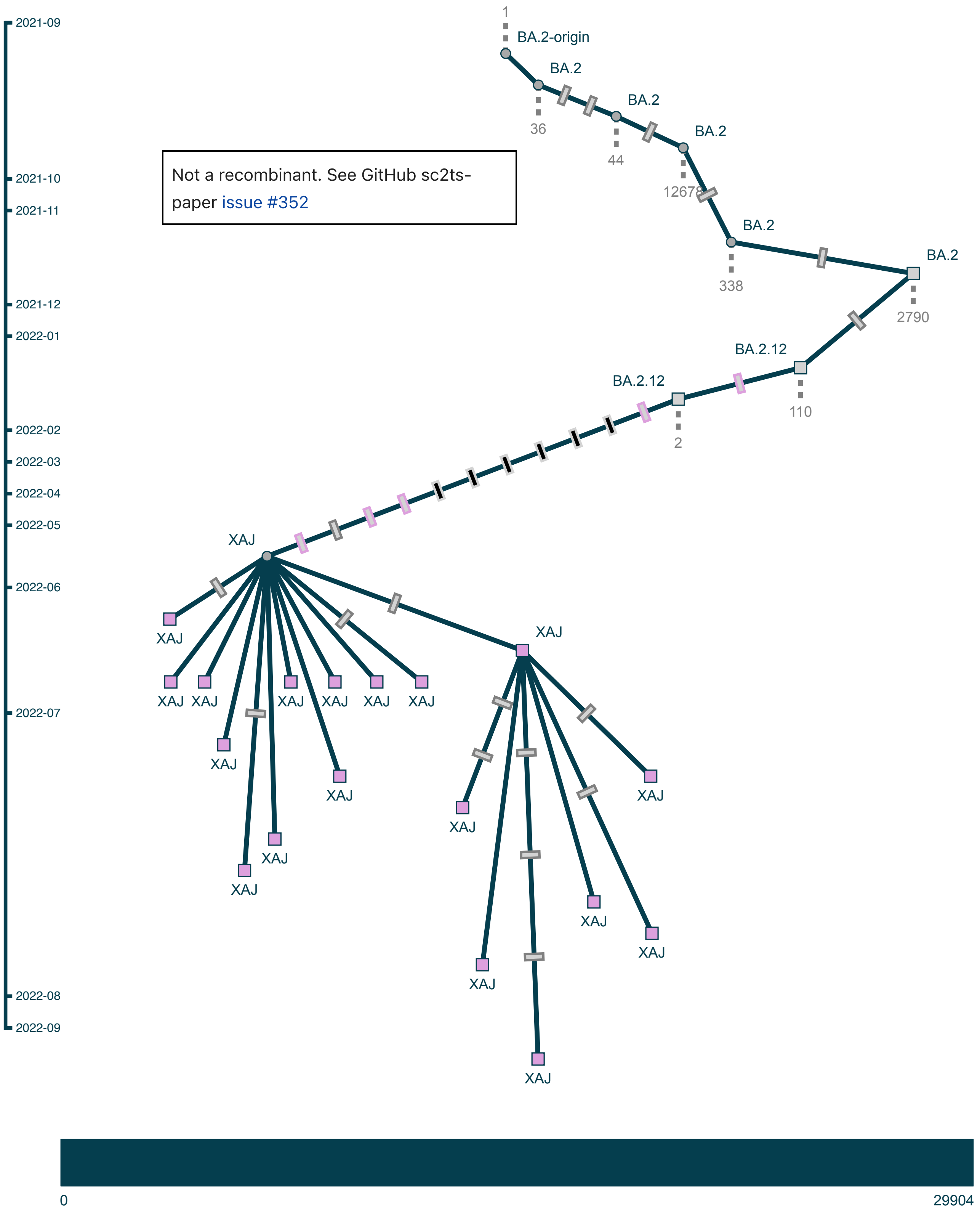

Subgraph of pango XAN/XAV: (20 samples, 20 shown)

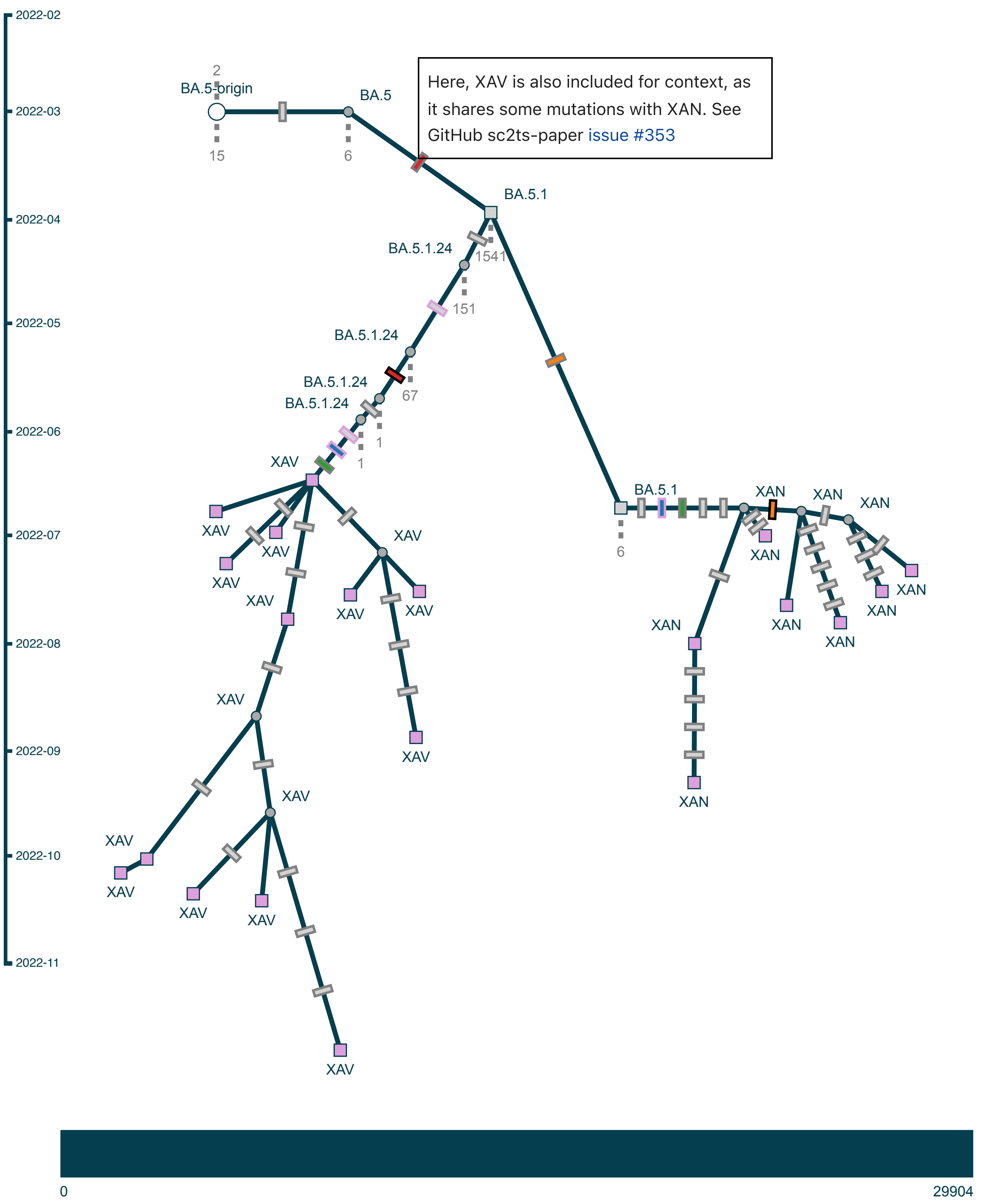

Subgraph of pango XAS: (77 samples, 77 shown)

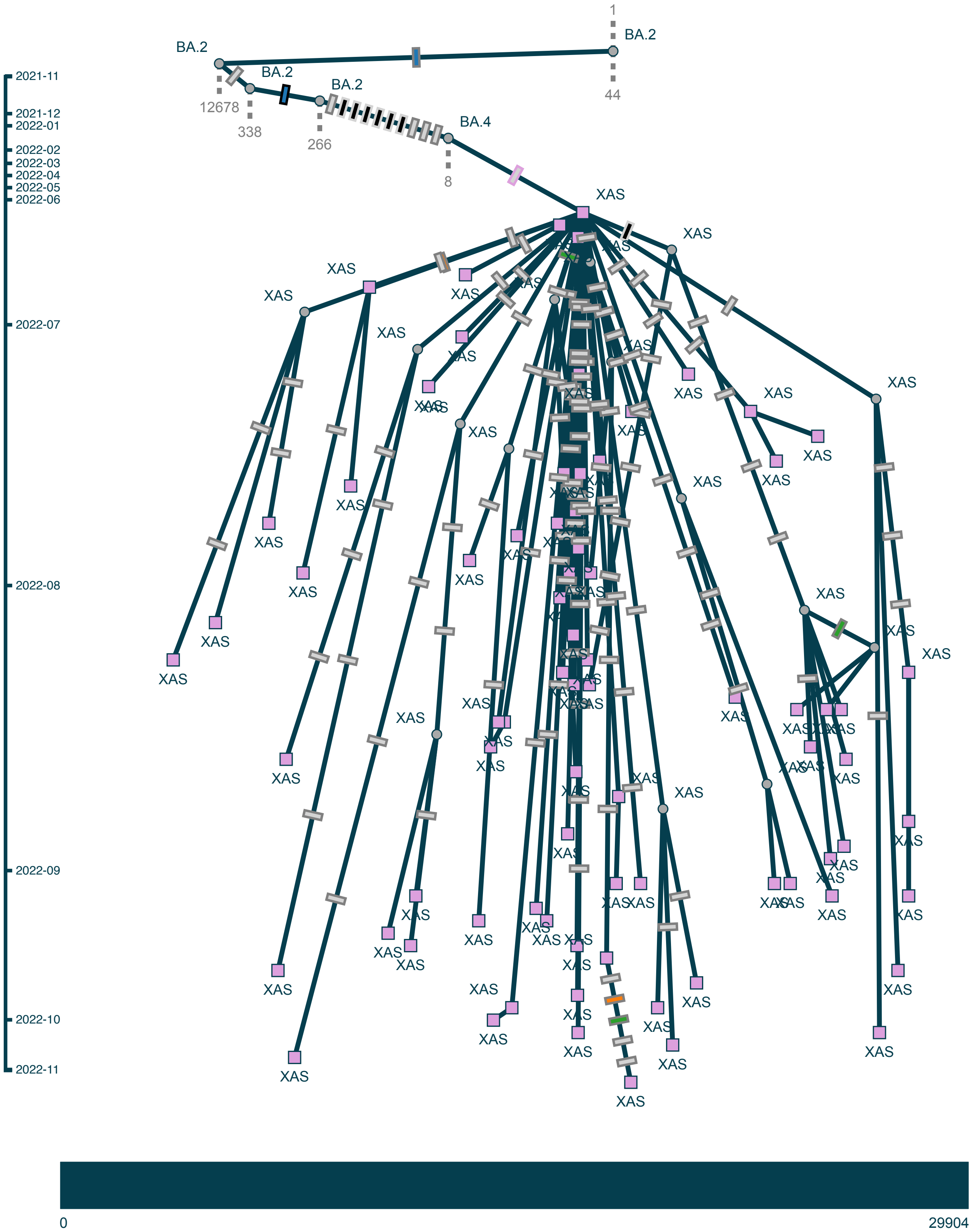

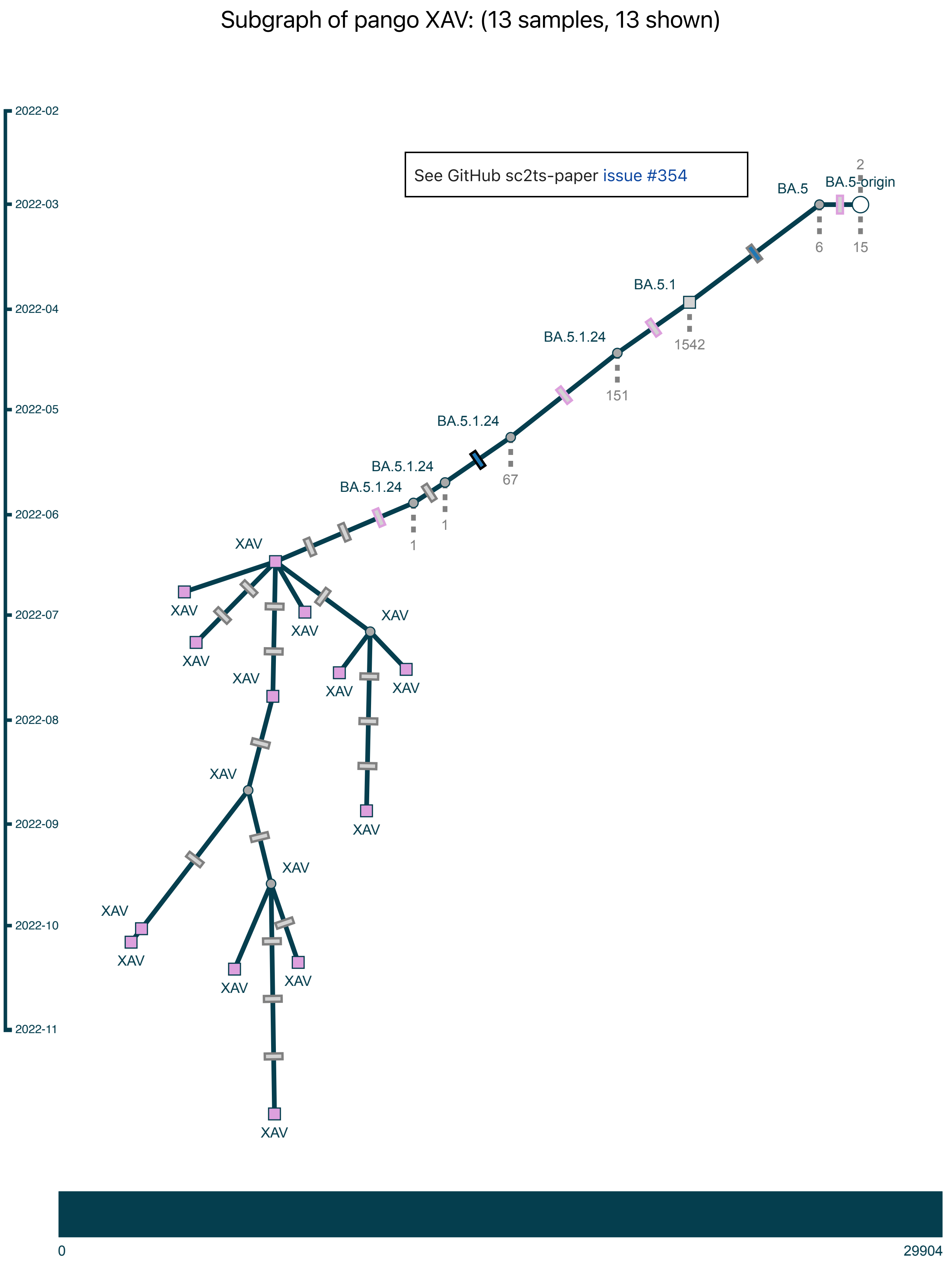

Subgraph of pango XAV: (13 samples, 13 shown)
See GitHub sc2ts-paper issue #354
2022-02
2022-03
2022-04
2022-05
2022-06
2022-07
2022-08
2022-09
2022-10
2022-11
BA.5
BA.5-origin
2
6
15
BA.5.1
1542
BA.5.1.24
151
BA.5.1.24
67
BA.5.1.24
BA.5.1.24
1
1
XAV
XAV
XAV
XAV
XAV
XAV
XAV
XAV
XAV
XAV
XAV
XAV
XAV
XAV
XAV
XAV
XAV
0
29904

Subgraph of pango XAZ: (133 samples, 20 shown)

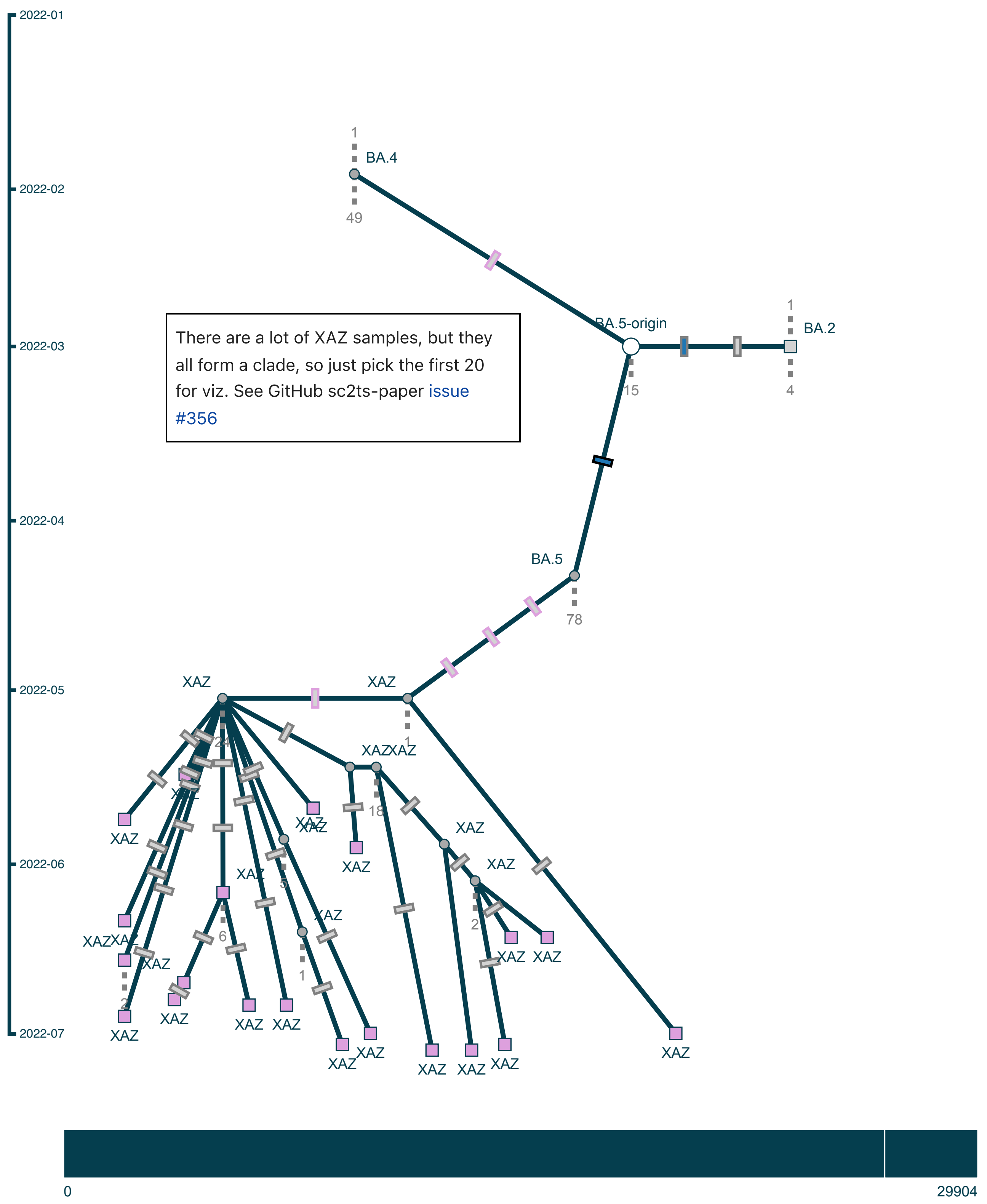

Subgraph of pango XBB: (71 samples, 20 shown)

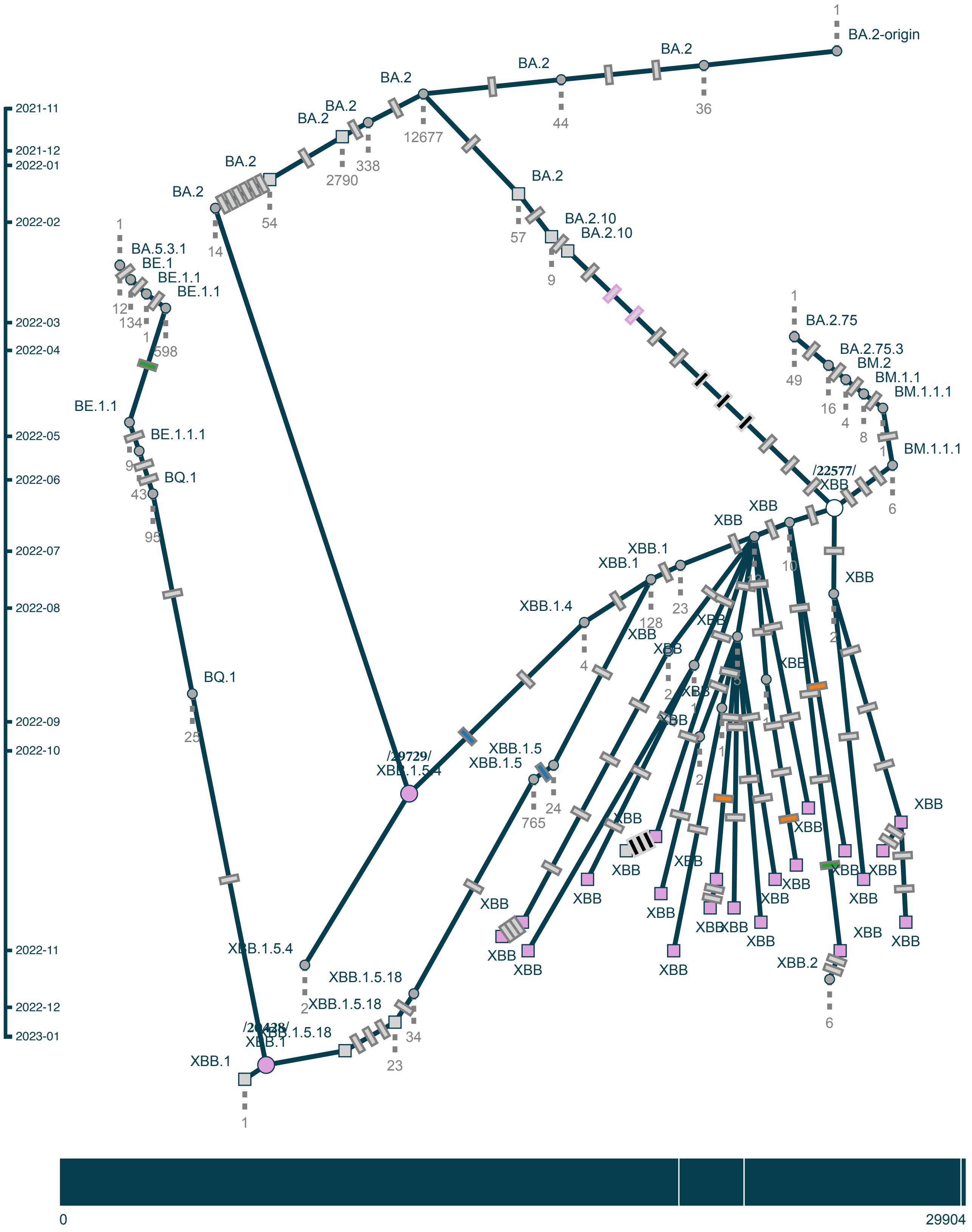

Subgraph of pango XBD: (30 samples, 30 shown)

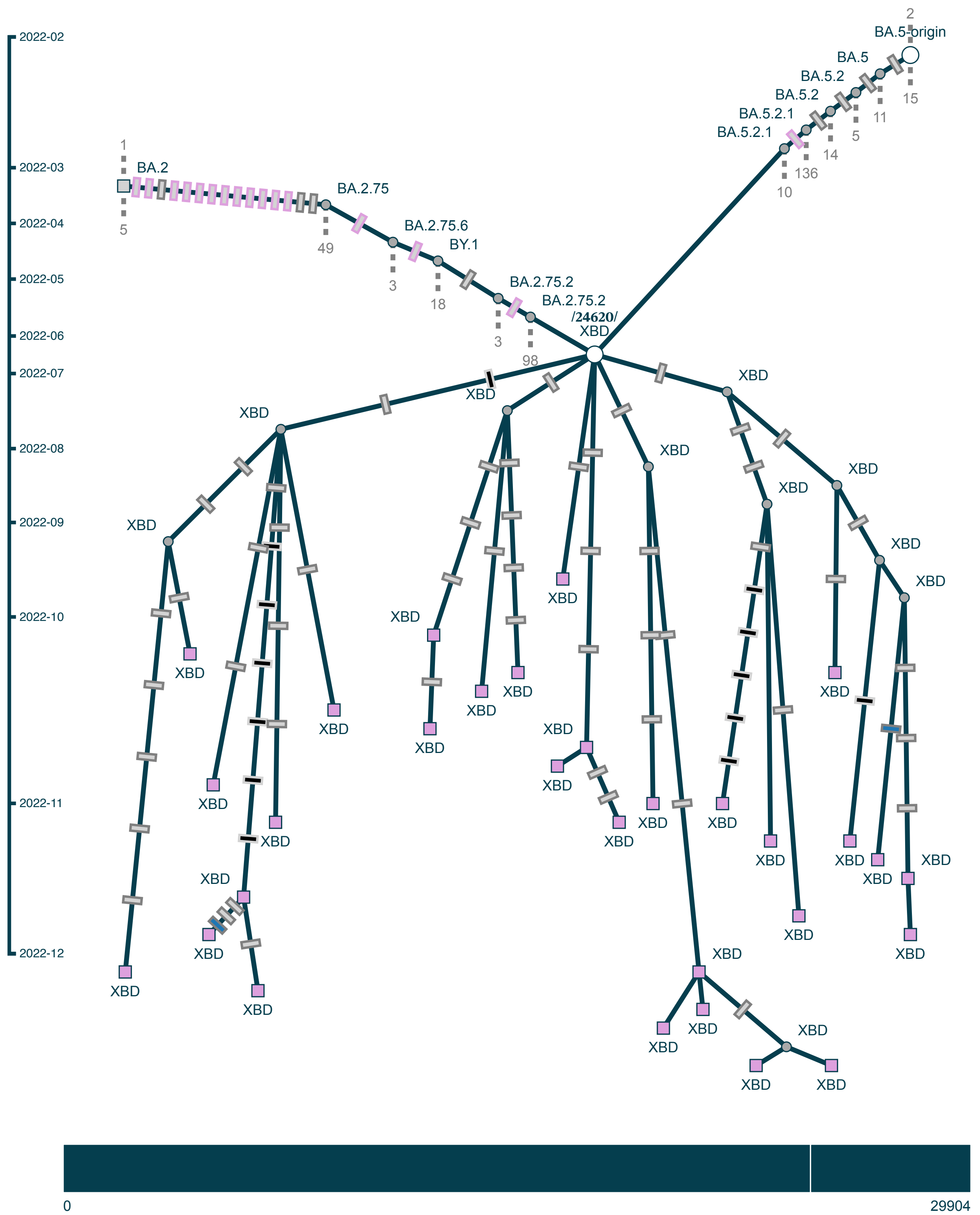

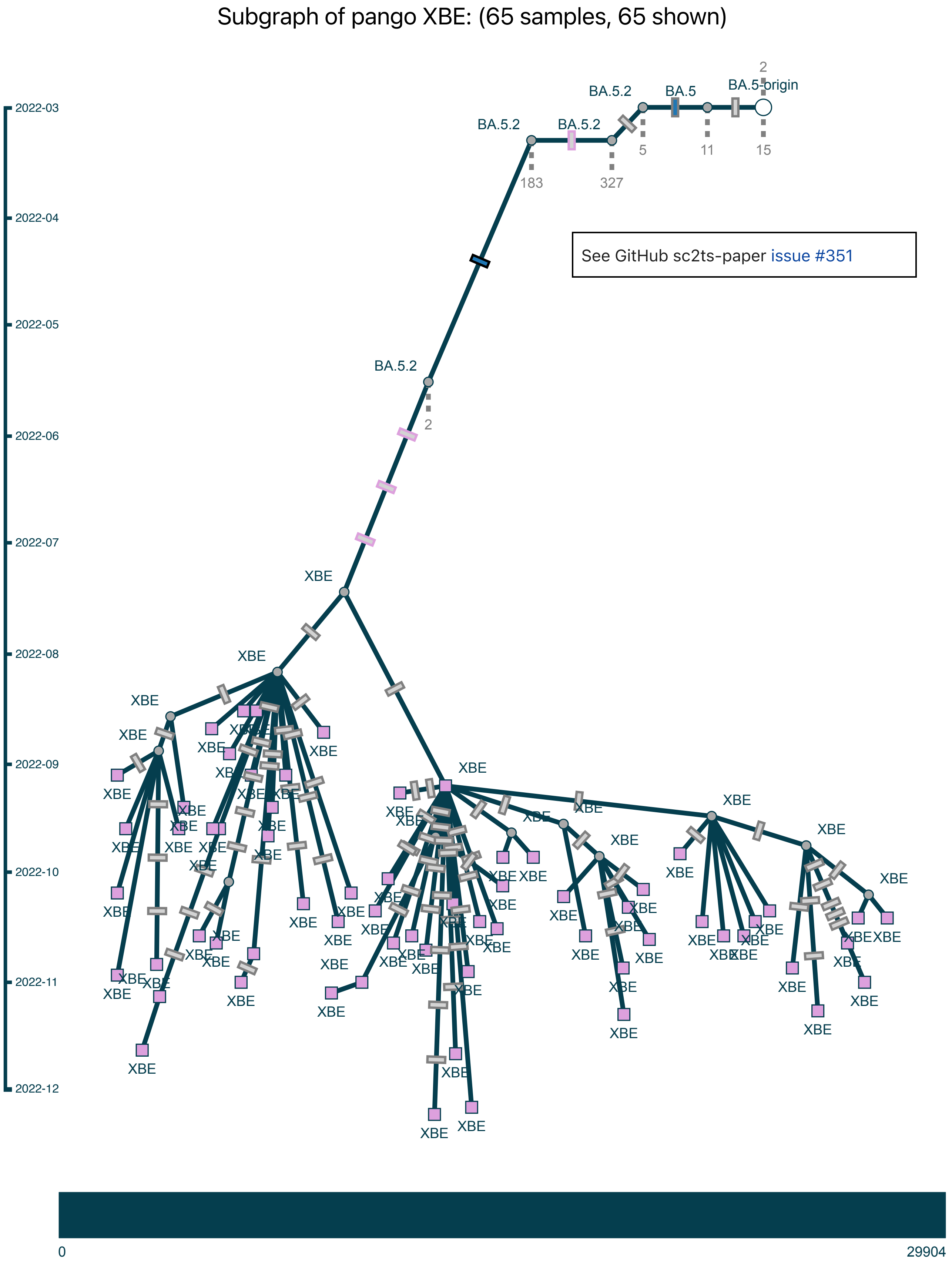

Subgraph of pango XBE: (65 samples, 65 shown)
2022-03
2022-04
2022-05
2022-06
2022-07
2022-08
2022-09
2022-10
2022-11
2022-12
BA.5.2
BA.5.2
BA.5.2
BA.5
BA.5-origin
BA.5.2
2
5
11
15
183
327
2
See GitHub sc2ts-paper issue #351
XBE
0
29904

Subgraph of pango XBF: (124 samples, 124 shown)

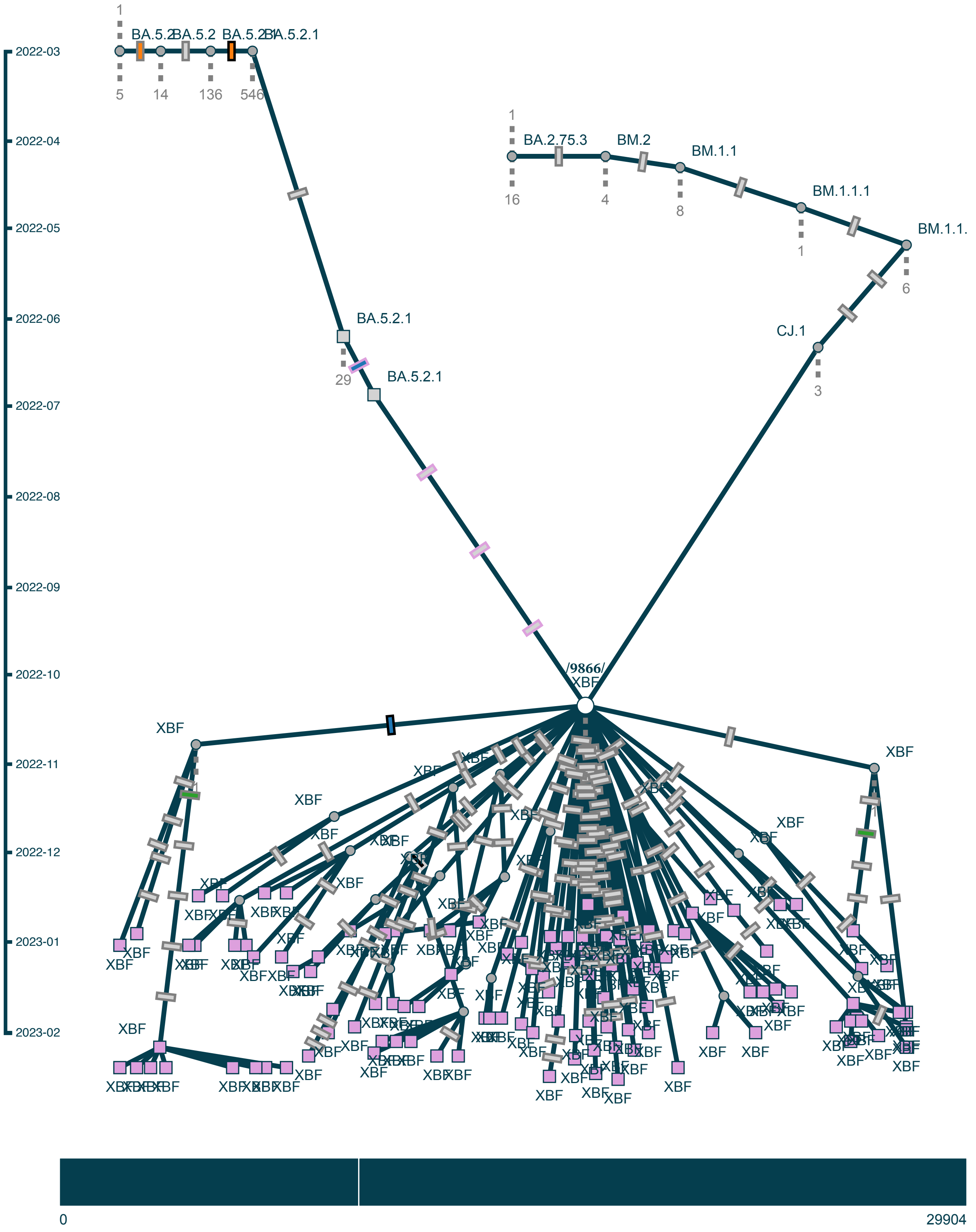

Subgraph of pango XBG: (25 samples, 25 shown)

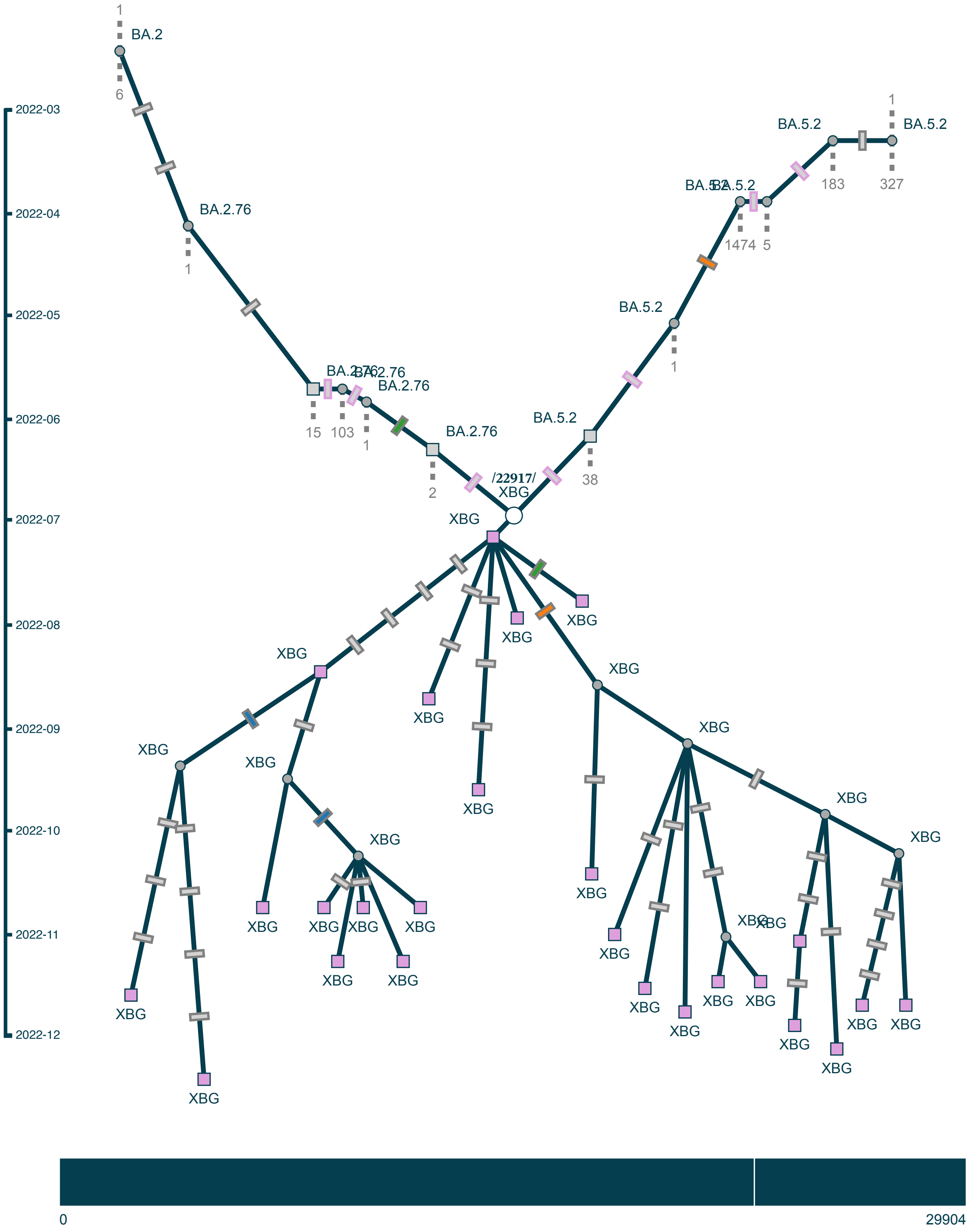

Subgraph of pango XBH: (2 samples, 2 shown)

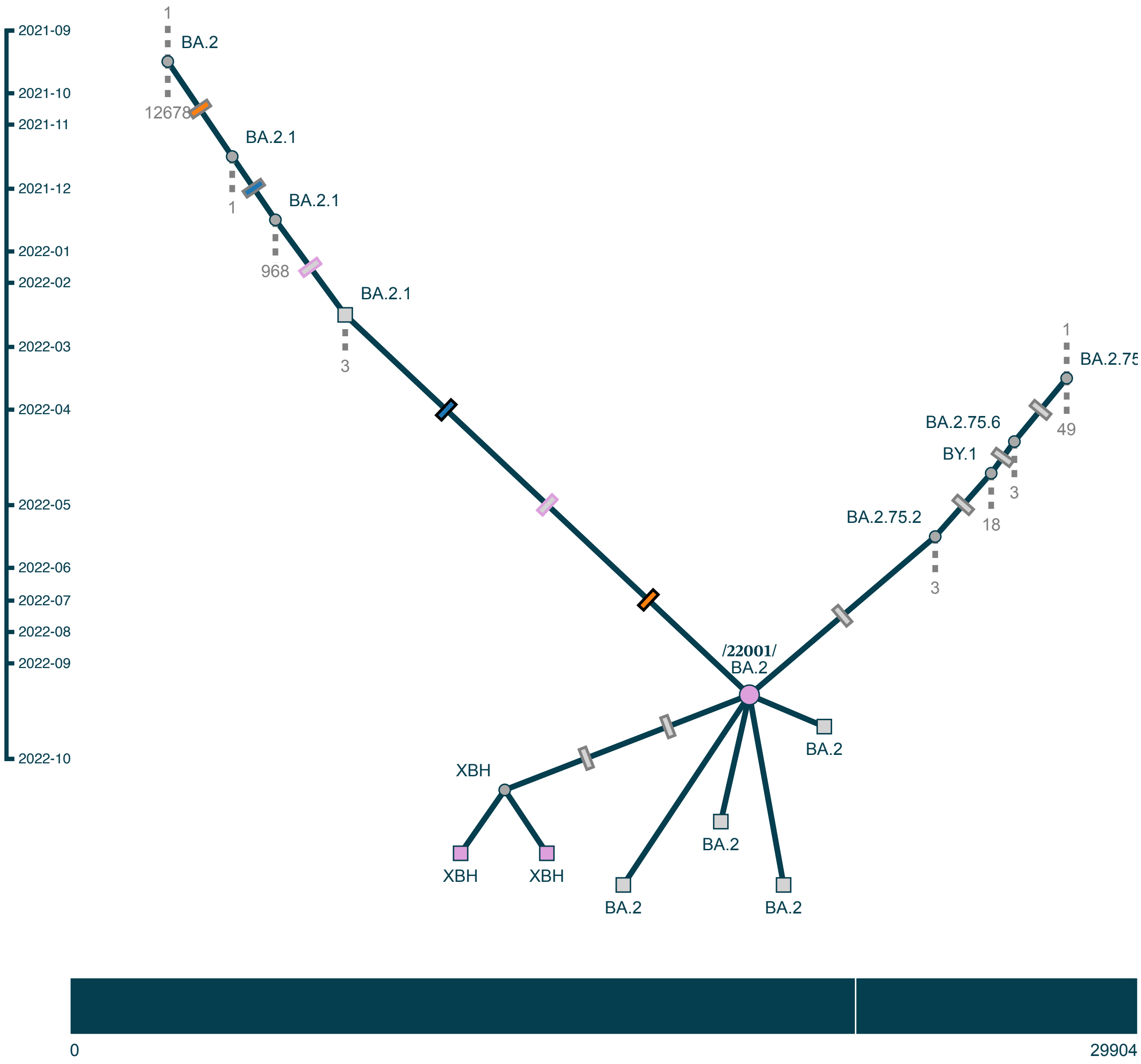

Subgraph of pango XBK/XBK.1/XBQ/CJ.1.3: (27 samples, 27 shown)

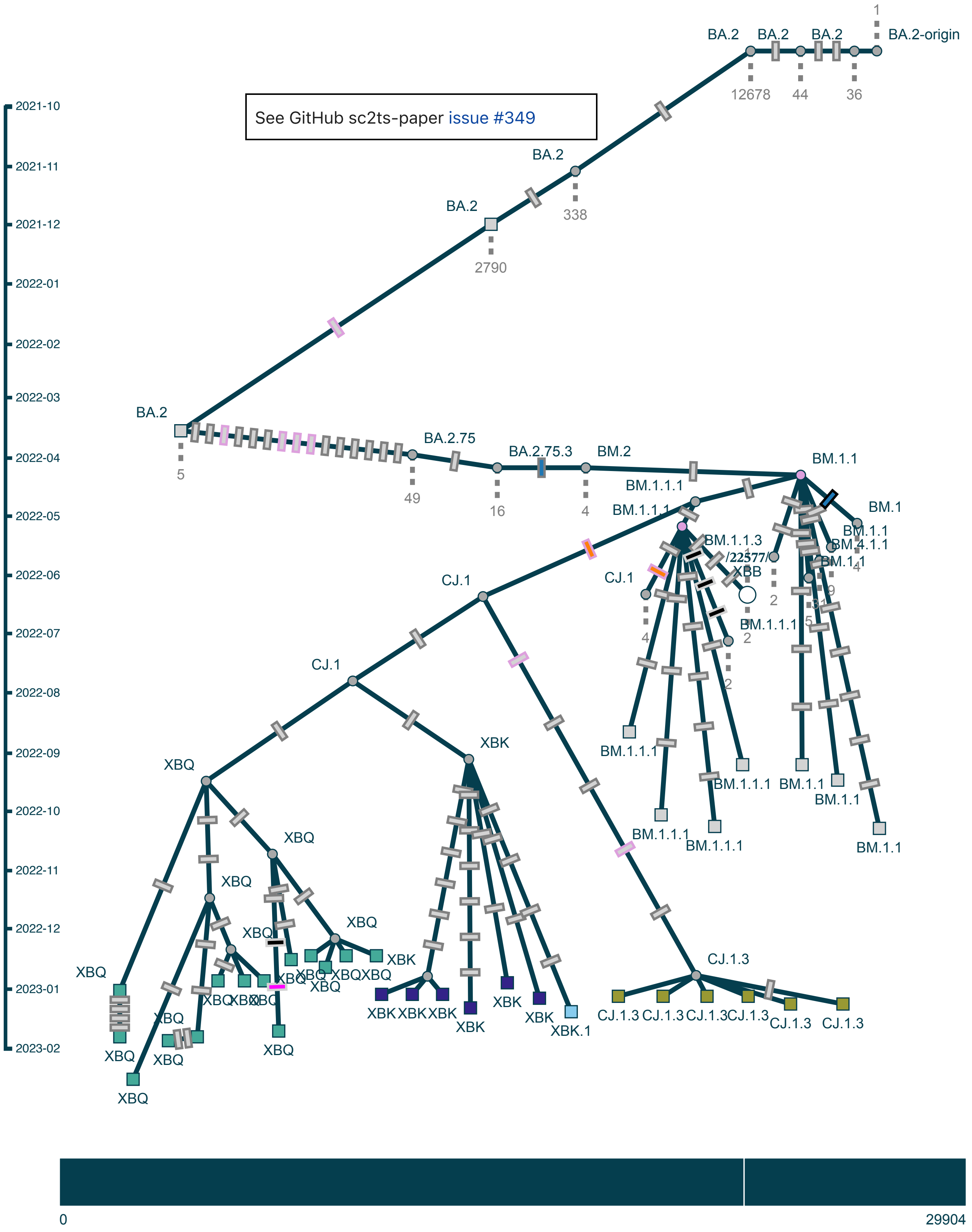

Subgraph of pango XBM: (10 samples, 10 shown)

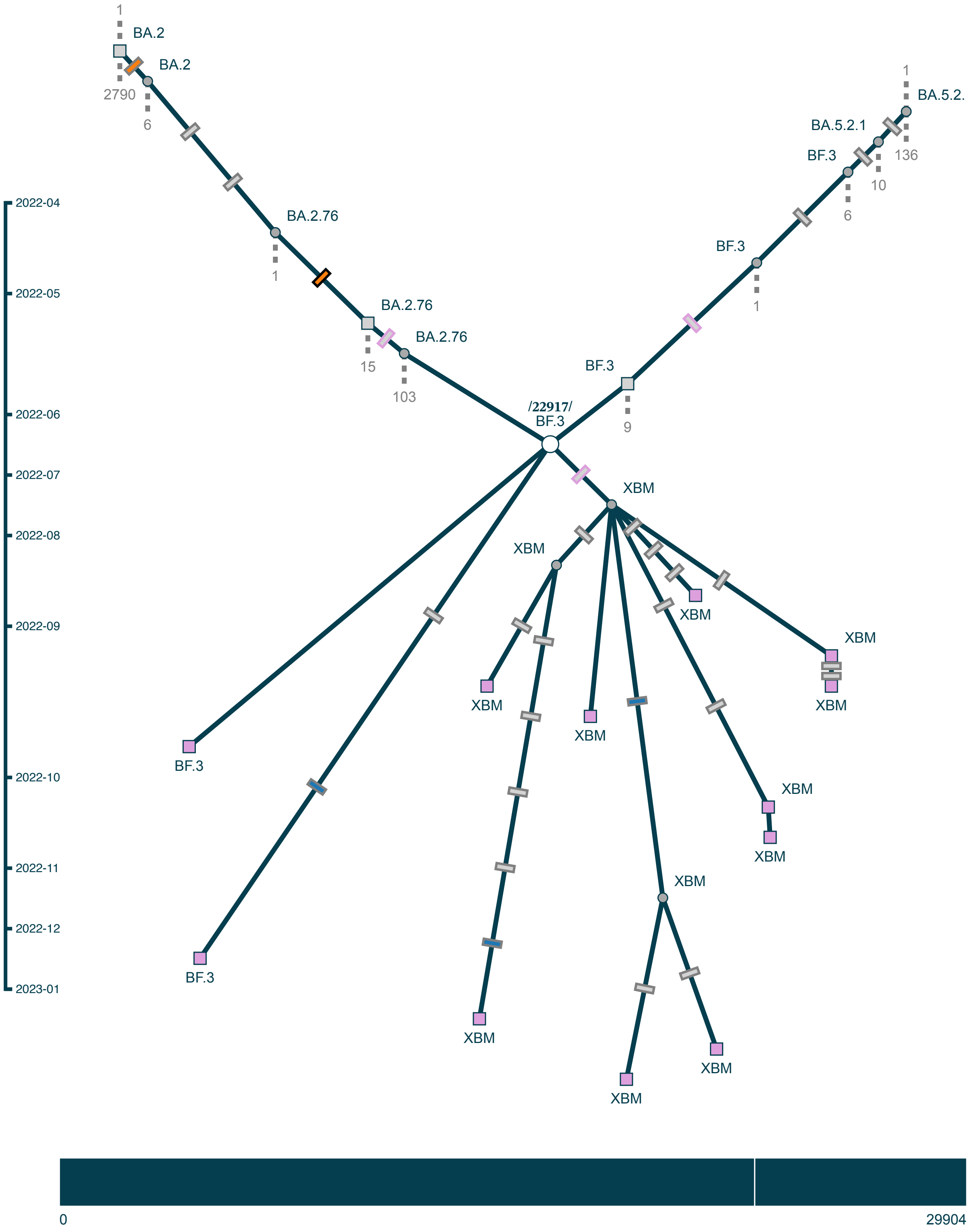

Subgraph of pango XBR: (1 sample, 1 shown)

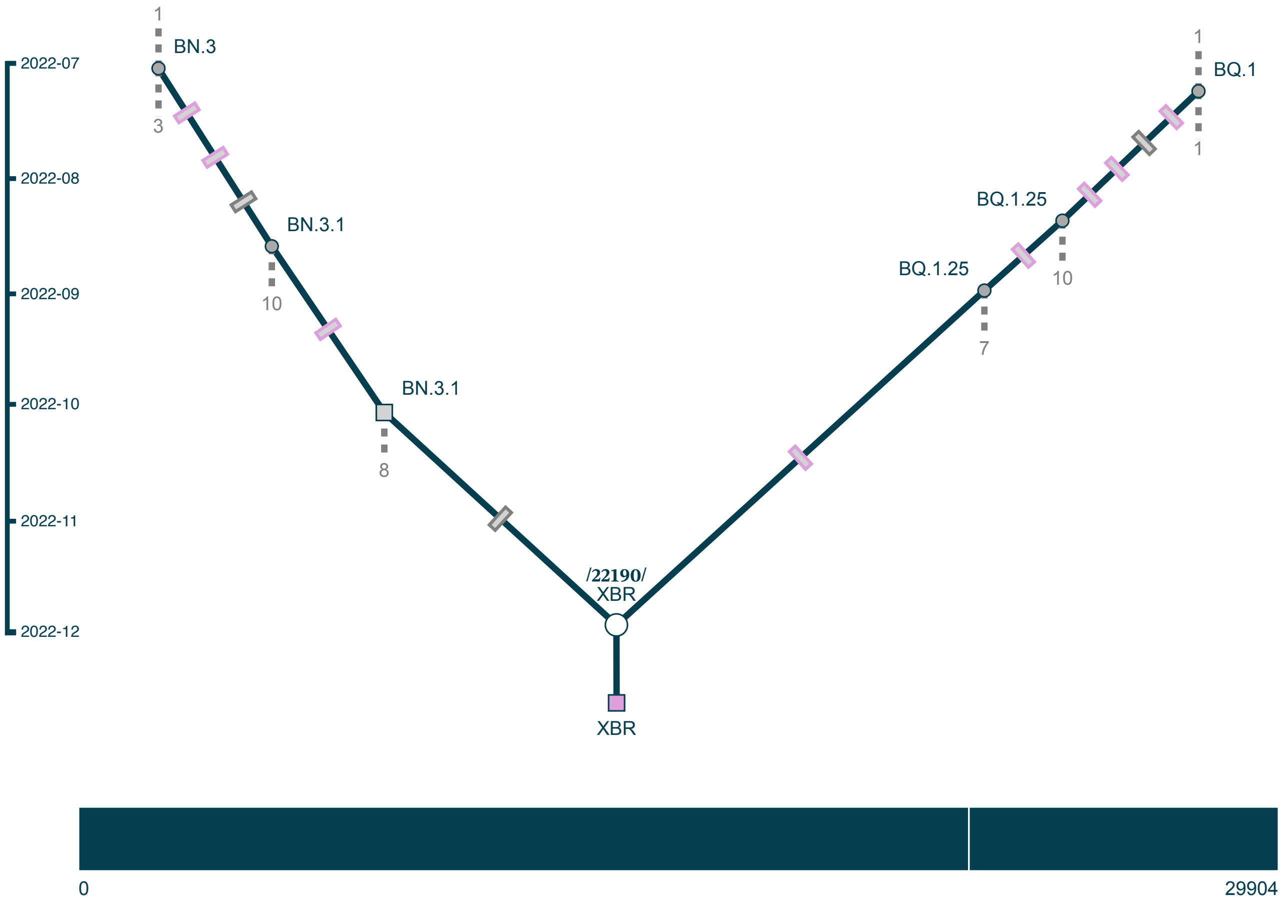

Supplement: Supplement 1 [file media-1.pdf]
