# Supplementary material for: A Pandemic-Scale Ancestral Recombination Graph for SARS-CoV-2
Source: bioRxiv. 2025 Nov 10:2023.06.08.544212. Preprint. [Version 2] doi: 10.1101/2023.06.08.544212 (PMC12642650; doi:10.1101/2023.06.08.544212)
Supplement: Supplementary file 1 [file NIHPP2023.06.08.544212V2-supplement-1.pdf]

bioRxiv preprint doi: https://doi.org/10.1101/2023.06.08.544212; this version posted November 10, 2025. The copyright holder for this preprint (which was not certified by peer review) is the author/funder, who has granted bioRxiv a license to display the preprint in perpetuity. It is made available under aCC-BY 4.0 International license.

Supplementary Material

S1.1 Parsimony comparison of sc2ts and UShER

We performed an in-depth analysis of the sc2ts and UShER ARGs (we refer to the UShER tree as an ARG for simplicity here, as a single tree is also an ARG) in terms of parsimony. A direct comparison is difficult because of the different alignments used as input, the different sets of sites used for inference and the fact that we are including deletions for a subset of sites in the sc2ts ARG.

After site remapping, and including all mutations involving deletion characters, sc2ts has 143,690 mutations more (2,078,370 vs 1,934,680). If we exclude mutations involving deletions in sc2ts, this is reduced to 46,371 more (2.3%) than UShER. If we restrict to the 26,192 sites that were not remapped, sc2ts has 4,785 (0.26%) fewer mutations than UShER, and has around 2.5 times more mutations than UShER on the 163 sites that were post-hoc remapped.

To provide a more objective measure of the overall parsimony of the inferred ARGs with respect to the Viridian alignments, we remapped all 29903 sites in both ARGs using Fitch-Hartigan parsimony. Here the nucleotides ACGT and "-" were treated as distinct characters, with all other values regarded as missing data. In this case, the sc2ts ARG has 629,864 (5.5%) fewer mutations (10,840,687 vs 11,470,551). Some of this difference may be due to the post-hoc incorporation of phylogenetic signal from deletions by sc2ts, but more work is required to fully understand the differences between the two approaches.

See notebook analysis_usher_sc2ts.ipynb for details.

S1.2 Imputation comparison of sc2ts and UShER

Both sc2ts and UShER support missing data in input alignments, and effectively "hard call" these values when the samples are integrated into the tree/ARG. In doing so, we impute the missing and ambiguous data for these samples. We evaluate the imputation performance of sc2ts and UShER by comparing their outputs on the Viridian alignments.

The total aligned dataset for 2,475,418 samples and 27,507 sites in the sc2ts and UShER intersection ARGs represents 63.41 GiB of nucleotide calls. Of the 162,838,461 missing data calls (Ns) in the alignments, sc2ts and UShER disagreed in their imputed values for only 203,579 (0.125%). Additionally, 956,859 calls made use of the IUPAC uncertainty codes. Of these sc2ts imputed 435 (0.05%) incorrectly (i.e., with a base that is not compatible with the ambiguity code). UShER imputed only 7 calls (0.007%) from this set incorrectly, likely due to special handling of ambiguous bases in the parsimony calculations.

This computation is a good example of type of integrated large-scale data analysis that can be easily performed using the combination of tskit and VCF Zarr. The entire analysis is performed in a short Jupyter notebook[155], with the core calculation requiring about 30 lines of Python code. Retrieving data from the base alignments and ARGs for 2.48 million samples at the same time over all 27,507 sites took a little under 5 minutes, with a peak memory usage of about 4.5GiB of RAM on a standard laptop.

See notebook example_data_processing.ipynb for details.

S1.3 Pango lineage "origination" events

To analyse how well the ARG reflects the phylogenetic structure implicit in the Pango lineage naming system, we considered the Pango assignments generated by Pangolin on the alignments for each node in the ARG (STAR Methods). If the ARG perfectly reflected the Pango lineage

bioRxiv preprint doi: https://doi.org/10.1101/2023.06.08.544212; this version posted November 10, 2025. The copyright holder for this preprint (which was not certified by peer review) is the author/funder, who has granted bioRxiv a license to display the preprint in perpetuity. It is made available under aCC-BY 4.0 International license.

structure, each lineage would form a clade descending from a single originating node with that label. We identified putative origination nodes among those labelled with a given lineage as the node with the maximum number of descendant samples and the earliest time (if there is a tie).

Of the 2058 distinct Pango lineages in the ARG, 1473 of these (comprising 717798 samples) match perfectly, with unique origination events in the ARG where all samples assigned a given lineage descend from the first node assigned that lineage. A further 245 lineages (589197 samples) match perfectly when we count the descendants of the parent of the first node (accounting for polytomies in which multiple originating nodes for a given lineage are siblings). We then have 306 lineages (755840 samples) where the difference in the number descendants of the first node's parent is $<$ 100. The remaining 35 lineages (420047 samples) are dominated by a few large lineages such as BA.1.1 (147271 samples) and AY.4.2 (54607 samples) which have multiple non-sibling origins within the ARG.

See notebook analysis_pango_events_in_arg.ipynb for details.

S1.4 Evaluating inferred node dates

To validate the accuracy of times for non-sample internal nodes inserted into the ARG (inferred using tsdate), we compared the dates of first emergence of Nextstrain clades against the Nextstrain SARS-CoV-2 tree (downloaded on 2023-01-21). Figure S8 shows relatively close agreement for most clades, with only three disagreeing by more than 28 days: 20C (an early lineage from 2020), 20E (Pango B.1.177 also known as EU1, emerging mid-2020), and 21B (Pango B.1.617.1 also known as Kappa, emerging in late 2020 to early 2021).

Then, to gauge internal consistency of the time estimation procedure, we selected a subset of sample nodes, matching the distribution of node times and average number of children of the non-sample internal nodes. These selected sample nodes were then re-dated, and the inferred dates compared against their recorded collection dates. The results in Figure S9 demonstrate that although the inferred and true times are generally very close, there is a clear bias with re-estimated times being consistently older (by an average of 37 days). This is in line with the findings of other recent work[94], and is likely to be a consequence of edges carrying more mutations than would be expected under a neutral molecular clock prior (either due to errors in tree-building or genuinely varying mutation rates), as well as inaccuracies in the recording of sample dates.

S1.5 Origins of major VOCs

S1.5.1 Alpha

The B.1.1.7 (Alpha) variant emerged in the UK as the first major VOC. Its origin is well reconstructed in the ARG, with Figure S10 showing it to be a saltational lineage characterized by a long branch preceding its origin. Most of the characteristic Alpha mutations[95] (20 out of 23) in the ARG occur immediately above the Alpha root. However, a characteristic three-base correlated mutation is instead associated with the parent node (labelled B.1.1) of the Alpha root, suggesting that this mutation was acquired prior to the emergence of Alpha. This parent node has an additional 5 children (leading to 12 samples), of which we display one (ERR4413600) with no associated mutations, meaning that it has an identical sequence to the inferred ancestor of Alpha. Below the root of Alpha, a single sample (ERR5178290) appears without the characteristic mutation C3267T (or any additional mutations). However, this is only a single sample and given that it also appears two months after the first Alpha samples we treat its placement with caution. Note that the list of mutations in ref[95] includes a T26801C in the M gene which should no longer

bioRxiv preprint doi: https://doi.org/10.1101/2023.06.08.544212; this version posted November 10, 2025. The copyright holder for this preprint (which was not certified by peer review) is the author/funder, who has granted bioRxiv a license to display the preprint in perpetuity. It is made available under aCC-BY 4.0 International license.

be considered characteristic of Alpha, as the Wuhan-Hu-1 reference at 26,801 has since been updated to C. In the ARG, a one-base non-coding deletion (position 28,271) is also associated with the Alpha root; although this is not in the original list of characteristic Alpha mutations, it has been suggested elsewhere as an Alpha-defining mutation[79].

S1.5.2 Delta

The B.1.617.2 lineage (Delta) was first detected in India[96]. The subgraph in Figure S11 shows a mutation-rich branch leading to the Delta root, which contains 11 single nucleotide mutations and a 6-base deletion. The placement of this branch in the ARG is a result of long branch splitting of the lineage above B.1.617.

Delta belongs to the “B.1.617” clade, which also includes Kappa (B.1.617.1) and B.1.617.3[96]. Previous phylogenetic analyses suggest that Delta and B.1.617.3 are more closely related to each other than either is to Kappa[97,98]. In the ARG, however, Delta and Kappa appear as sibling lineages (see the subgraph in Figure S11). This reflects the closest approximation to the relationships suggested in the previous studies that could be inferred, given that no B.1.617.3 samples passed our QC filters and no seed B.1.617.3 samples were specified for ARG inference.

In the ARG, all six of the shared Delta-Kappa mutations (red) occur above the B.1-labelled common ancestor of Kappa (left branch) and Delta (right branch). All nine of the Kappa-specific mutations (blue) occur above the B.1.617-labelled node leading to Kappa samples. Seven out of nine Delta-specific mutations (purple) occur above the Delta root (the other two are above the MRCA of Kappa and Delta). We further examined the mutations associated with two major branches of Delta. Using a phylogenetic analysis, Stern et al.[99] suggest that the Delta sublineages can be classified into distinct clades and identified the characteristic mutations of these clades. In the Nextstrain phylogeny shown in Stern et al.[99], the first major split separates clades A to D from clade E; this topology is reflected in the ARG. All three of the clade A-D mutations (G15451A, C16466T, and a 6-base deletion at 28,248) occur on the uppermost branch of Delta (which corresponds to clades A to D). There are ten clade E mutations: five occur on the right branch of Delta (which corresponds to clade E) and three (C1191T, C5184T, and C28253T) occur above the Delta root. The placement of these mutations in the ARG generally reflects what is proposed in Stern et al.[99].

There are four positions with multiple mutations (orange at position 1267, blue at 210, green at 20396, and red at 25276). Of these, the only clear topological change that could make the graph more parsimonious would be to attach the node above SRR12316669 one position higher up the tree, which would allow the placement of two recurrent red mutations (a C25276T above SRR12316669 and another above ERR4561562 / B.1.617) rather than one C25276T mutation followed by two reversions. The other recurrent mutations can only be made more parsimonious by topological rearrangements that would cause recurrence at other sites. We therefore believe that the inferred Delta origins in the sc2ts ARG are largely correct.

S1.5.3 Omicron BA.1 and BA.2

The B.1.1.529 lineage (Omicron) is believed to have emerged in southern Africa, and it is a saltational lineage with two highly successful sublineages, BA.1 and BA.2[100]. BA.1 and BA.2 are genetically distinct from each other and from their parental B.1.1.529 lineage[101]. In the subgraph shown in Figure S12, BA.1 and BA.2 differ from the non-Omicron B.1.1 ancestor (above the B.1.1.529-labelled MRCA of BA.1 and BA.2) by 27 mutations (including two deletions), reflecting its saltational origin. The ARG captures the split between BA.1 and BA.2 shown in previous phylogenetic analysis[100]. The 5-base deletion highlighted in green above the BA.2 root is an artefact of our naive deletion remapping procedure, which splits two 9-base deletions in BA.1

bioRxiv preprint doi: https://doi.org/10.1101/2023.06.08.544212; this version posted November 10, 2025. The copyright holder for this preprint (which was not certified by peer review) is the author/funder, who has granted bioRxiv a license to display the preprint in perpetuity. It is made available under aCC-BY 4.0 International license.

and BA.2 into three deletions (one in green at 11288, one in blue at 11283, and one in orange 11292; see Document S1.6). Also, there is uncertainty in the sequence alignments leading to ambiguity in the start position of these deletions (if these deletions occur at 11283, there would be a single deletion event before the BA.1/BA.2 split), as noted in the Pango designation issue[101].

We selected a BA.1 seed sample that led to the fewest mutations (see `https://github.com/jeromekelleher/sc2ts-paper/issues/264`). This strategy resulted in five recurrent mutations highlighted in blue/orange/green/red/purple which are absent in the chosen seed but present in many of its inferred descendants. Inspection of the subgraph reveals that an alternative choice for a seed would be the later sample SRR17051902. Seeding with this sample would be likely to generate a topology in which those five recurrent mutations are shared between BA.1 and BA.2 and subsequently reverted in a number of the deep branching BA.1 lineages such as those to ERR7671333 and ERR7858953.

S1.6 Analysis of deletion events

Although uncommon relative to single nucleotide mutations, deletions are an important component of SARS-CoV-2 evolution[102], contributing to the emergence of VOCs by enhancing, e.g., efficiency for cell entry[156] and potential for antibody escape[103]. Using the ARG, we identified major deletion events (which are propagated to many descendants) as well as recurrent deletions (which occur on at least two branches) in the ARG. In Table S1, we list the major deletion events in the ARG which are each inherited by at least 10,000 descendant samples; and in Table S2, we list the recurrent deletions in the ARG which are each observed in at least 10,000 (0.4%) samples. A deletion is said to be observed in a sample here if the gap character is present at the specified positions, and the immediately adjacent positions are not the gap character.

Most of the deletions in these tables occur in coding regions (except the 1-base deletions at 28,271) and do not cause frame shifts, involving 3, 6, or 9 sites (except the 4-base deletion and the two 5-base deletions, which are likely artefacts; see below). All the deletions in these tables (besides the artefactual cases) have been linked to the emergence of major VOCs (Alpha[95], Delta[99], Omicron B.1.1.529[101], BA.1[101], BA.2[101], and BA.4[157]) and/or reported to be prevalent in previous studies of deletions[79]. All these major deletion events (again, besides the artefactual cases) are associated with the origins of major VOCs in the ARG (see the Alpha subgraph in Figure S10; the Delta subgraph in Figure S11; and the Omicron subgraph in Figure S12), except the 1-base deletion at 28,271 on node 1436808 and the 9-base deletion at 686 on the BA.4-labeled node 2698748 (Table S1). Most of these deletions have been identified as characteristic mutations of the major VOCs in the studies or analysis cited in Table S1. Notably, eight of the major deletion events occur in the N-terminal domain of the S1 unit of Spike (a known hotspot of recurrent deletions[103] that plays a substantial role in antigenicity[158]), which are all highly recurrent in the ARG (Table S2). These include known recurrent deletions: the 6-base deletion at 21,765 and the 3-base deletion at 21,991, which are associated with increased efficiency in cell entry[105] and antibody escape[103], respectively. In addition, we found recurrent deletions in ORF8 and NSP3, which have been suggested to be indel hotspots[159]. Together, these findings demonstrate that the ARG captures genuine deletion events in SARS-CoV-2 evolution. A comprehensive evolutionary analysis of the deletion events in the ARG is beyond the scope of the current study; however, we believe that the ARG, as a resource, will enable and facilitate future studies to better understand the role of deletions in SARS-CoV-2 evolution (alongside recombination and single nucleotide mutations), which to date has been understudied in great part due to a lack of suitable data models, methods, and tools.

Detection of deletions and inference of deletion events remain challenging, especially when the dataset is enormous. Here we took a naive approach to map deletions onto the ARG,

bioRxiv preprint doi: https://doi.org/10.1101/2023.06.08.544212; this version posted November 10, 2025. The copyright holder for this preprint (which was not certified by peer review) is the author/funder, who has granted bioRxiv a license to display the preprint in perpetuity. It is made available under aCC-BY 4.0 International license.

Table S1: Major deletion events in the ARG. The deletions caused by these events are inherited by at least 10,000 samples. The deletions are characterized by a site position in the genome (start) and the number of sites involved (length); they are annotated by the gene in which they occur (non-coding otherwise). The IDs of the nodes in the ARG above which the deletions occur and the Pango lineages assigned to these nodes are shown. Frequency is the percentage of samples inheriting a deletion. If a deletion has been identified as a characteristic mutation of a VOC by a study (or an analysis), a reference to the study (or the analysis) is provided. Abbreviations: NSP, non-structural protein; ORF, open reading frame; S, Spike; S1-NTD, N-terminal domain in Spike S1 subunit.

Start	Length	Region	Node	Pango lineage	Frequency (%)	Ref
28271	1	non-coding	1436808	B.1	45.06	
28248	6	ORF8	220186	B.1.617.2 (Delta)	44.91	[99]
22029	6	S / S1-NTD	200039	B.1.617.2 (Delta)	44.82	[99]
11288	4	ORF1ab / NSP6	1436802	B.1.1.529 (Omicron)	35.12	
28362	9	N	1436802	B.1.1.529 (Omicron)	35.12	[101]
21633	9	S / S1-NTD	822854	BA.2 (Omicron)	21.44	[101]
11292	5	ORF1ab / NSP6	822854	BA.2 (Omicron)	21.43	
6513	3	ORF1ab / NSP3	851246	BA.1 (Omicron)	13.73	[101]
11283	5	ORF1ab / NSP6	851246	BA.1 (Omicron)	13.69	
21765	6	S / S1-NTD	851246	BA.1 (Omicron)	13.68	[101]
21987	9	S / S1-NTD	851246	BA.1 (Omicron)	13.68	[101]
22194	3	S / S1-NTD	851246	BA.1 (Omicron)	12.60	[101]
28271	1	non-coding	86456	B.1.1.7 (Alpha)	11.70	
11288	9	ORF1ab / NSP6	86456	B.1.1.7 (Alpha)	11.70	[95]
21991	3	S / S1-NTD	86456	B.1.1.7 (Alpha)	11.64	[95]
21765	6	S / S1-NTD	86456	B.1.1.7 (Alpha)	11.70	[95]
21765	6	S / S1-NTD	1265302	BA.4 (Omicron)	7.67	[157]
686	9	ORF1ab / NSP1	2698748	BA.4 (Omicron)	0.94	

treating each site involved in a deletion as independent of the other sites. Consequently, some deletion events might be split up and appear as multiple smaller deletion events, which are likely artefactual. The three contiguous out-of-frame deletions in NSP6 (5-base at 11,283, 4-base at 11,288, and 5-base at 11,292) are examples of such artefacts (Table S1). In the sequence alignments, there are two 9-base deletions, one at 11,283 and the other at 11,288, which are found in 339,810 BA.1 samples and 843,291 BA.2 samples in the Viridian v04 dataset, respectively. Assuming that the alignments are correct, there should be two deletion events, but they were mapped as three deletion events as they had four overlapping sites from 11,288 to 11,292, which were inferred by parsimony as the 4-base deletion event at 11,288. Future improvements in deletion remapping should remove such artefactual deletions.

S1.7 Pango X lineage events

Most of the Pango X lineages had straightforward origination events as defined in Section S1.3 and are represented in Table 1. Three lineages were treated specially, corresponding to 23 omitted samples in total.

XM has 4 separate origins, 3 singletons and one dominant node with 26 descendants. We include the dominant node in Table 1 and omit the singletons. See Document S1.9.9 for further

bioRxiv preprint doi: https://doi.org/10.1101/2023.06.08.544212; this version posted November 10, 2025. The copyright holder for this preprint (which was not certified by peer review) is the author/funder, who has granted bioRxiv a license to display the preprint in perpetuity. It is made available under aCC-BY 4.0 International license.

Table S2: Recurrent deletions in the ARG. These deletions occur on at least two branches in the ARG and are found in the alignments of at least 10,000 (0.4%) samples in the Viridian v04 dataset. For each deletion, the number of occurrences in the ARG and the number of samples where it is observed are shown. For further explanation, see the caption of Table S1. Abbreviations: NSP, non-structural protein; ORF, open reading frame; S, Spike; S1-NTD, N-terminal domain in Spike S1 subunit.

Start	Length	Region	Occurrences	Samples
686	9	ORF1ab / NSP1	6436	43354
21991	3	S / S1-NTD	2088	309629
21765	6	S / S1-NTD	482	820194
22029	6	S / S1-NTD	275	1107941
22194	3	S / S1-NTD	200	310349
6513	3	ORF1ab / NSP3	181	341380
21987	9	S / S1-NTD	70	338633
28248	6	ORF8	28	1113392
28271	1	non-coding	24	1331409
11288	9	ORF1ab / NSP6	19	843291
11283	9	ORF1ab / NSP6	13	339810
28362	9	N	7	850483
21633	9	S / S1-NTD	7	531973

Table S3: Concordance among methods in characterizing Pango X lineages. These comparisons focus on the Pango X lineages associated with the Type 1 recombination events (Table 1) For each method, we calculated the number of Pango X lineages which have inferred parent Pango lineages and breakpoint intervals concordant with those proposed by the community. The denominators indicate the number of Pango X lineages for which detection results are available from the original studies[40,41]. For the Pango X lineages with non-overlapping breakpoint intervals, the distances between the inferred breakpoint intervals and those proposed by the community are shown (median and range).

	Parent lineages	Breakpoint intervals	
Method	Concordant (%)	Concordant (%)	Distance (bases)
Sc2ts	14 / 16 (87.5)	14 / 16 (87.5)	540.5 (315, 766)
RecombinHunt-GISAID	13 / 15 (86.7)	10 / 15 (66.7)	764.0 (4, 1202)
RecombinHunt-Nextstrain	12 / 15 (80.0)	11 / 15 (73.3)	1160.0 (559, 2785)
CovRecomb	7 / 8 (87.5)	4 / 8 (50.0)	1562.5 (217, 13208)

Table S4: Validation of sc2ts recombinants by 3SEQ, CovRecomb and rebar over all 855 recombinants and the 354 passing and 501 failing QC.

Method	Total	QC Pass	QC Fail
3SEQ	484	338	146
CovRecomb	92	90	2
rebar	213	203	10

bioRxiv preprint doi: https://doi.org/10.1101/2023.06.08.544212; this version posted November 10, 2025. The copyright holder for this preprint (which was not certified by peer review) is the author/funder, who has granted bioRxiv a license to display the preprint in perpetuity. It is made available under aCC-BY 4.0 International license.

details. XAC has 4 separate origins, and has been omitted from Table 1. However, as Figure 2B shows, these are all descending from a single BA.2 classified node descending from the putative Xx event. Similarly, XAD has two independent origins descending from the Xx event, and we omit it for simplicity. See Document S1.9.16 for more details on the Xx event.

In cases where nested samples not assigned to the focal Pango lineage are either from different Pango X lineages or given a generic BA.2 assignment. Of these 59 generic assignments 6 had conflicts in their UShER placements and could have been assigned an X lineage (XJ: 3, XU: 2, XN: 1), and 25 were cases in which Scorpio[143] failed to find a specific lineage (accounting for 7% of the 357 "Omicron (Unassigned)" Scorpio calls across 2,747,985 alignments).

See notebook tab_pango_x_events.ipynb for details.

S1.8 Analysis of the Jackson et al. recombinants

Jackson et al.[29] conducted a detailed analysis of early recombinants involving B.1.1.7 (Alpha), including the first designated Pango X lineage, XA. The authors first performed a targeted search for candidate recombinants by scanning sampled sequences for sequence motifs associated with Alpha, followed by an analysis to reduce the list of plausible recombinants displaying signs of onward transmission. Here we compared the sc2ts detection results of these recombinants with the detection results reported by Jackson et al.

Jackson et al. described four groups of recombinants (named A to D) and four singleton recombinants. Of the 16 samples analysed by Jackson et al., 12 were included in the ARG; the remaining 4 were excluded because the sequences did not pass the QC filtering criteria or because of a high HMM cost. Groups A-D are summarized in Table S5 and show excellent concordance in terms of the parental Pango lineages and breakpoint intervals. All four groups are directly associated with strongly supported recombination events in the ARG in terms of the number of averted mutations (A: 16, B: 6, C: 30, D: 18). In the ARG, the singleton CAMC-CBA018 has the parents B.1.177+B.1.1.7 and the breakpoint interval 16,177–20,133, and is strongly supported by 14 averted mutations. In the Jackson et al. results, this singleton is almost identical with the same parent lineages and a breakpoint interval of 11,395—21,993. In the ARG, the singleton MILK-103C712 has the parents B.1.177.16+B.1.1.7 and a breakpoint interval of 24915–27972, again strongly supported by 11 averted mutations. In the Jackson et al. results, this lineage has the same parents, with the breakpoint interval 26,800–27,974. In the ARG, the singleton QEUH-1067DEF has the parents B.1.1.7+B.1.177.9 and a breakpoint interval of 7,729–10,870, supported by 11 averted mutations. In the Jackson et al. results, this lineage has the same parents, with the breakpoint interval 6,953–10,872.

See notebook analysis_jackson_recombinants.ipynb for details.

S1.9 Additional analyses of the Pango X lineages

In this section we provide some additional analysis on Pango X lineages in the ARG. These analyses are intended to be read in conjunction with the subgraph illustrations in Document S2.

S1.9.1 XA

XA traces to a well supported recombination node, with no reversions. Note that the "causal" sample ERR5308556 (which triggers the initial recombination event) is identical to the recombination node, and therefore lacks C8090T, which is one of the XA consensus mutations (identified as those shared by over 90% of XA samples). As mentioned elsewhere, the sc2ts detection results in terms of the Pango lineages of the parents and the breakpoint intervals agree with the

bioRxiv preprint doi: https://doi.org/10.1101/2023.06.08.544212; this version posted November 10, 2025. The copyright holder for this preprint (which was not certified by peer review) is the author/funder, who has granted bioRxiv a license to display the preprint in perpetuity. It is made available under aCC-BY 4.0 International license.

Table S5: Comparison of recombination breakpoint intervals and parent lineages for Groups A-D reported by Jackson et al. with the corresponding recombination events in the sc2ts ARG. The second column gives the number of sequences in the group. See the text for details of the groups and the sequences included. limited to the samples considered by Jackson et al. The breakpoint coordinates in Table 1 of Jackson et al. have been altered as follows: we subtract one to the left coordinates and add one to the right coordinates to correspond to the `tskit` definition of inheritance on either side of a breakpoint, and add one to the right coordinate to make the intervals right-exclusive.

Group	Sequences	Method	Breakpoint interval	Parent lineages
A (XA)	2	Jackson	21,254–21,767	B.1.177+B.1.1.7
		`sc2ts`	20,411–21,765	B.1.177.18+B.1.1.7
B	2	Jackson	6,527–6,956	B.1.36.28+B.1.1.7
		`sc2ts`	6,529–6,954	B.1.36.28+B.1.1.7
C	3	Jackson	24,913–28,653	B.1.1.7+B.1.221
		`sc2ts`	25,997–27,972	B.1.1.7+B.1.221.1
D	3	Jackson	21,574–23,065	B.1.36.17+B.1.1.7
		`sc2ts`	22,445–23,063	B.1.36.39+B.1.1.7

results reported by Jackson et al. (Table S5). Two of the Jackson et al. XA samples are present in the ARG (ERR5308556 and ERR5414941); these samples are specifically marked in Figure 2, which is a summary of this subgraph.

S1.9.2 XB

The displayed subgraph shows a small selection of the 192 XB samples. XB was originally defined as a recombinant between the closely related lineages B.1.634 (left parent) and B.1.631 (right parent) from North and Central America[32]. However, these parent lineages were not added to the ARG due to high HMM costs. Together with other lineages such as B.1.627 (sister to B.1.631[32]), they are likely to attach somewhere along the long branch in the subgraph visualisation (with > 30 mutations). If samples from these parental lineages were available for attachment, then XB would likely be detected as a recombinant by sc2ts.

See github:sc2ts-paper/discussions/780 for further details.

S1.9.3 XC

The five XC samples descend from a well supported recombination node. The two parents that are both identical to actual samples (ERR4908740 on the left and DRR321274 on the right). There is one reversion (G21987A) on the left side of the breakpoint, but the site 21987 was masked during ARG inference because it was identified to be experiencing frequent mutation in a preliminary ARG.

S1.9.4 XE/XH

The XE samples descend from a well supported recombination node, with one mutation and one reversion (T22792C). This reversion was mapped onto the ARG as a part of postprocessing, and could be placed more parsimoniously by a single C22792T that groups together XH and the

bioRxiv preprint doi: https://doi.org/10.1101/2023.06.08.544212; this version posted November 10, 2025. The copyright holder for this preprint (which was not certified by peer review) is the author/funder, who has granted bioRxiv a license to display the preprint in perpetuity. It is made available under aCC-BY 4.0 International license.

lineage leading to the BA.2 recombination nodes displayed on the lower left. Nevertheless, the exact arrangement of the mutations at position 22792 should not affect the proposed breakpoint, which is very distant, at position 11283. This breakpoint coincides with a repeated homopolymeric sequence involving 5-base deletions, so that alignment errors could have a small effect on the breakpoint position. Note that the two lower recombination nodes are both well supported, the youngest being postulated as the origin of XAF (see below).

See github:sc2ts-paper/discussions/633 for further details.

S1.9.5 XF

XF is a well-supported recombinant between Delta and Omicron BA.1, with the breakpoint on the far left of the genome. All XF samples are the unique descendants of a well supported recombination node, with both parent nodes (ERR6193241: AY.4 and ERR8042040: BA.1) and the single child node (ERR8076129) being samples.

S1.9.6 XG

The three XG samples descend from a reconstructed internal node, which in turn descends from a well supported recombination node. The left parent is a sample (SRR20179863), and the right parent is a reconstructed internal node assigned to Omicron BA.2, which is supported by a sample (ERR9124067) that is identical to it.

S1.9.7 XJ

All the XJ samples descend from a well supported recombination node. There are 14 samples (including e.g. ERR8683190) which sc2ts identified as descendants of the same recombination node but are labelled BA.2 by pangolin (displayed on the left), and which do not possess the XJ consensus mutations C22792T and C27945T. These BA.2 samples could be worthy of further investigation.

S1.9.8 XL

All the XL samples descend from a well supported recombination node, with three child samples, which are identical to it. The left parent is a sample (ERR7989424), and the right parent is a reconstructed internal node assigned to BA.2.5 with a large number of descendant samples.

S1.9.9 XM/XAL

XM is a recombinant of BA.1 (left parent) and BA.2 (right parent). Sc2ts identifies multiple recombinant origins of XM. One of these origins leads to a dominant clade (node 1003220 at position 21,595), which also contains the only three XAL samples.

Independent evidence for multiple XM origins comes from consideration of a 9-base deletion in the Spike gene at position 21633, which is characteristic of BA.2 but not BA.1 `https://github.com/cov-lineages/pango-designation/issues/361`. Most of the XM-labeled samples in ARG have this deletion, but three (SRR19024311, ERR9447529, and SRR18775874) do not, suggesting that XM samples trace to at least two recombination events, one to the right of the deletion (which leads to the majority of the XM samples), and one to the right. In the subgraph, it can be seen that one of the samples, SRR18775874, has been (presumably wrongly)

bioRxiv preprint doi: https://doi.org/10.1101/2023.06.08.544212; this version posted November 10, 2025. The copyright holder for this preprint (which was not certified by peer review) is the author/funder, who has granted bioRxiv a license to display the preprint in perpetuity. It is made available under aCC-BY 4.0 International license.

placed within the main XM deletion-containing clade. As sc2ts does not use deletions for inference, deletion remapping (STAR Methods) has reverted the deletion in this case, creating many insertions on the branch above the sample (magenta). A better use of deletions in sc2ts would likely result in placing this sample within one of the two XM clades which do not have the deletion.

See github:sc2ts-paper/discussions/656 for further details.

S1.9.10 XN/XAU

In the sc2ts ARG, the initial XN sample (ERR8986821) is well explained as a non-recombinant, copying from an inferred internal node assigned to BA.2 with only one mutation. This internal node is directly supported by the sample ERR9502469. All the other XN samples descend from ERR8986821. A small clade containing XAU is the sister to XN, and there are a series of samples (ERR9453882, ERR9502469, and ERR9823912) which support the gradual emergence of both groups from a BA.2 ancestral lineage via accumulation of single mutations.

The evidence supporting the original designation of XN and XAU as recombinants may come from inaccurate or problematic data sources. In the case of XN, support for a recombinant BA.1+BA.2 origin is from three mutations that are claimed to be BA-1-specific: C241T, A2832G, and C3037T (https://github.com/cov-lineages/pango-designation/issues/480). However, the BA.1/BA.2 split (https://github.com/cov-lineages/pango-designation/issues/361) does not list C241T and C3037T as BA.1-specific, meaning that these two mutations are not supportive of recombination. Moreover, although the remaining mutation claimed to support a recombinant origin, A2832G, is typical of BA.1 and not BA.2, it is seen in several BA.2 samples (e.g. ERR9453882), supporting the idea that it has independently arisen within BA.2. Hence, none of the sites used to designate XN as a recombinant reliably do so.

In the case of XAU, a recombinant origin was proposed on the basis of the same three sites, plus C2470T (https://github.com/cov-lineages/pango-designation/issues/894). However, C2470T is not BA.1-specific (https://github.com/cov-lineages/pango-designation/issues/361). Moreover, some BA.2-designated samples such as ERR9823912 also have C2470T, so we do not believe this to be a recombinant-supporting mutation either.

See github:sc2ts-paper/discussions/781 for further details on XN.

See github:sc2ts-paper/discussions/928 for further details on XAU.

S1.9.11 XP

This is a case where the Pango X lineage is designated on the basis of two loci on the far right of the genome: a single nucleotide mutation (A29510C) and a rare, large deletion at position 29734 in the S2M step loop (https://github.com/cov-lineages/pango-designation/issues/481), which is not common enough to map in sc2ts during postprocessing and hence not shown here. Therefore, unless this deletion were given more weight than a single locus, sc2ts would not identify XP as a recombinant, even if deletions were used during ARG inference. If this deletion were recurrent, the evidence from sc2ts suggests that XP might not be a recombinant, because it is only one mutation different from the sample ERR8628084.

See github:sc2ts-paper/discussions/775 for further details.

S1.9.12 XQ/XR/XU/XAA/XAG/XAM

In the ARG, XR, XU, XAA, XAG, and XAM are nested under the XQ recombinant lineage. These lineages were designated around the same time, approximately within a five-month period (Mar. to Jul., 2022). The breakpoint intervals of these lineages, as suggested in the Pango designation

bioRxiv preprint doi: https://doi.org/10.1101/2023.06.08.544212; this version posted November 10, 2025. The copyright holder for this preprint (which was not certified by peer review) is the author/funder, who has granted bioRxiv a license to display the preprint in perpetuity. It is made available under aCC-BY 4.0 International license.

issues, all occur at the 5' end of the genome, within a ∼5 kb segment (∼4,300 to ∼9,400): XQ (4321-5387), XAG (6515-8394), XU (6517-9345), XAM (8087-9193), and XAA (8392-9345).

Upon closer inspection, the right-hand branch above the recombination node appears problematic, as it displays three reversion mutations, which revert mutations at positions 22792, 24023, and 25624 in the ancestral lineage just above. These sites were excluded during ARG inference, and remapped onto the ARG by parsimony during ARG postprocessing. If these three sites were used during primary ARG inference, the right parent of the recombination node may have been chosen to be the ancestral BA.2 sample on the right-hand path with 18 child nodes. A more parsimonious reconstruction would then have the XQ clade as a sibling to a group of (monophyletic) XAA, XAM, XAG, and XR lineages. This would remove between 4 and 5 reversion mutations.

See github:sc2ts-paper/discussions/655 for further details.

S1.9.13 XS

In the ARG, the XS samples appear to have originated from two sequential recombination events (separated by a single branch). However, the later event is likely an artifact, because the XS sample (SRR18343220) associated with this event failed detection-level QC (i.e., the minimum net number of supporting loci; see the lower copying pattern). In addition, this sample was sequenced using the Ion AmpliSeq V1 kit, which was also used to sequence eight other samples descending from the same event (clustered due to a shared sequencing error due to AmpliSeq). These eight samples would likely attach to the nodes descending from the first recombination event. We excluded this likely artefactual event from further analysis and consider XS to have emerged from a single recombinant origin.

See github:sc2ts-paper/discussions/782 for further details.

S1.9.14 XW

All the XW samples descend from a well supported recombination node. Its left parent is a BA.1.1.15-labelled sample (ERR8069658), and its right parent is a BA.2-labelled sample (ERR9086669).

S1.9.15 XY

All the XY samples descend from a well supported recombination node, through a shared reconstructed internal node. Its left parent is a reconstructed internal node assigned to BA.1.1, which is supported by a sample (ERR8627048) identical to it. Its right parent is a BA.2-labelled sample (ERR9399983). There are a pair of (orange) reversion mutations within the XY clade at position 23948: these were not used for inference but remapped using parsimony during postprocessing, and indicate that the topology within the XY clade could potentially be improved by alternative phylogenetic reconstruction methods.

S1.9.16 Xx: XZ/XAC/XAD/XAE/XAP

This nested set of Pango X lineages descends from a single recombination node in the ARG, which we tentatively refer to as "Xx". Note that all were designated around the same time, with their samples collected roughly within a three-month period (Mar., 2022 to Jul., 2022). Several samples labelled as BA.2 by Pangolin also lie within this recombinant clade, including two that are identical to the proposed originating recombinant (ERR8146303 and ERR8163061).

bioRxiv preprint doi: https://doi.org/10.1101/2023.06.08.544212; this version posted November 10, 2025. The copyright holder for this preprint (which was not certified by peer review) is the author/funder, who has granted bioRxiv a license to display the preprint in perpetuity. It is made available under aCC-BY 4.0 International license.

Exact relationships within the Xx clade are somewhat tentative. For example, there are three highly mutable sites (22792, 28877, and 28878) which were not used during inference, but were remapped onto the ARG during postprocessing. The inferred topology places these sites as recurrent (coloured orange, grey, and faded yellow), but a more parsimonious arrangement would, for example, group the two XAD samples as siblings (removing the need to postulate mutations A1250G, A28877T, and G28878C to be recurrent). It is possible that a more parsimonious arrangement would also reduce the recurrent mutations and reversions seen at sites 22,792 (four orange mutations) and 26,060 (two parallel brown mutations), but we do not show such a reconstruction, which is complicated by the presence of additional (non-displayed) samples descending from internal nodes in the plotted subgraph.

For XAC, in particular, the Pango designation issue (`https://github.com/cov-lineages/pango-designation/issues/590`) suggests it to be a recombinant with two breakpoints: BA.2 on the left, BA.1 in the middle, and a switch back to a very small region of BA.2 on the right. This contrasts with the one-breakpoint BA.2+BA.1 origin inferred in the ARG. However, only two loci support the right hand BA.2 assignment: A29510C and a large deletion (deletions are treated as missing data during primary ARG inference). Sc2ts does not count this as enough evidence to insert an additional breakpoint.

See github:sc2ts-paper/discussions/631 for further details.

S1.9.17 XAF

Of the 21 XAF samples in the Viridian v04 dataset, only one XAF sample passed our QC filters and was added to the ARG. However, we find a small recombinant BA1.1/BA.2 clade of 37 samples, associated with the BA.2-labelled sample ERR9755541, within which the single XAF sample is nested. The inferred parents and breakpoint at 10,447 aligns with that proposed for XAF (`https://github.com/cov-lineages/pango-designation/issues/676`). Inspection of the omitted XAF samples with high HMM cost shows that they would attach as close sisters to the included XAF sample, albeit on branches with many new mutations.

The mutation-rich branch in the subgraph with a 5-base deletion (11283-11287) and insertion (11292-11296) may be due to a simple deletion alignment error.

See github:sc2ts-paper/discussions/638 for further details.

S1.9.18 XAJ

In the Pango designation issue (`https://github.com/cov-lineages/pango-designation/issues/826`), XAJ is suggested to be a recombinant between BA.2.12.1 (left parent) and BA.4 (right parent). However, XAJ is not inferred to be a recombinant lineage in the ARG. From the subgraph, which shows the 18 XAJ samples plus a small number of relevant BA.4 samples, it can be seen that 9 sites leading to XAJ indeed recur in the lineage to the closest BA.4 sample (SRR18848050), which strongly indicates that this is a recombinant that is missed by sc2ts. The reason that is is not a recombinant in the ARG is that 6 sites represent a 6bp deletion and so are not used for inference. The remaining three sites (T22917G, T23018G, and G23040A) are not enough to exceed our k=4 threshold for recombinant designation. Including deletions in inference would certainly lead to XAJ being detected as a recombinant, and with a lower threshold of k=3 the distribution of mutations on the ARG topology suggests that it is is even possible that XAJ is a 2-breakpoint recombinant with the region from 21765-23535 coming from the BA.4 labelled sample SRR18848050.

See github:sc2ts-paper/discussions/634 for further details.

bioRxiv preprint doi: https://doi.org/10.1101/2023.06.08.544212; this version posted November 10, 2025. The copyright holder for this preprint (which was not certified by peer review) is the author/funder, who has granted bioRxiv a license to display the preprint in perpetuity. It is made available under aCC-BY 4.0 International license.

S1.9.19 XAN/XAV

XAN and XAV are shown in this subgraph, as they share some mutations. In the Pango designation issues (`https://github.com/cov-lineages/pango-designation/issues/771`; `https://github.com/cov-lineages/pango-designation/issues/911`), XAN and XAV are suggested to be a recombinant between BA.2 (left parent) and BA.5 (right parent). Both these Pango lineages were detected as non-recombinants by sc2ts, because the number of supporting loci fell below our threshold of detection. In the ARG, we found only one mutation (C9866T) supporting the suggested BA.2 parent for both XAN and XAV.

See github:sc2ts-paper/discussions/783 for further details on XAN.

See github:sc2ts-paper/discussions/785 for further details on XAV.

S1.9.20 XAS

XAS was proposed as a recombination between BA.5 (left parent) and BA.2 (right parent). However, we find that this is only two mutations different from e.g. ERR9929846, which is a BA.4 sample. In fact, XAS only differs from the BA.4 root by a single mutation, C27945T, which was identified in the original Pango designation issue (`https://github.com/cov-lineages/pango-designation/issues/882`) as an important ORF truncation mutation which is also present in a few BA.2.65 samples. It is possible that C27945T was acquired by recombination, but this is clearly below our threshold of detection.

See github:sc2ts-paper/discussions/784 for further details.

S1.9.21 XAZ

In the Pango designation issue (`https://github.com/cov-lineages/pango-designation/issues/797`), XAZ is suggested as a two-breakpoint recombinant between BA.2.5 and BA.5 (the middle parent), with three sites supporting the BA.2.5 parent on the left end (C2232T, C3317T, and T3358C). In the ARG, this is very close to being a recombinant of BA.2.5 (ERR9615610) on the left side of the breakpoint, as not only are the three mutations present in this sample, but there is another site which supports recombination C1912T, which is not reported in the Pango designation issue). XAZ has C44T which is lacking in all BA.2.5 samples, leaving only three net supporting loci for a putative recombinant. However, position 44 is often clipped, so this is not strong evidence against a recombinant, and therefore XAZ is a marginal case of recombination. We found no evidence for the third segment inherited from a BA2.5 parent on the right end. In the ARG, all the XAZ samples form a clade. Here we display only the first 20 XAZ samples.

See github:sc2ts-paper/discussions/786 for further details.

S1.9.22 XBB

Most of the XBB samples descend from a well supported recombination node. In the Pango designation issue (`https://github.com/cov-lineages/pango-designation/issues/1058`), the Pango lineages of the suggested left and right parents are BJ.1 and BM.1.1.1, respectively. In the ARG, the Pango lineage of the right parent is identical, but that of the left parent is a related ancestral Pango lineage (BA.2.10 instead of BA.2.10.1.1 = BJ.1). This is likely due to the absence of BJ.1 in the ARG: only four BJ.1 samples exist in the Viridian v04 dataset, a single one passed sequence-level QC, and that was then rejected due to high HMM cost. Note that BJ.1 differs from BA.2.10 by over 20 mutations (`https://github.com/cov-lineages/pango-designation/issues/915`), explaining the large number of (gold) de novo mutations in the XBB copying pattern. Had BJ.1 samples been present in the ARG, the left branch of the

bioRxiv preprint doi: https://doi.org/10.1101/2023.06.08.544212; this version posted November 10, 2025. The copyright holder for this preprint (which was not certified by peer review) is the author/funder, who has granted bioRxiv a license to display the preprint in perpetuity. It is made available under aCC-BY 4.0 International license.

XBB-associated recombination node may have attached to a BJ.1-labelled node, and the origin of XBB would required fewer mutations.

See github:sc2ts-paper/discussions/929 for further details.

S1.9.23 XBD

All the XBD samples descend from a well supported recombination node. Its left and right parents are reconstructed internal nodes, both of which are directly supported by samples (SRR21382561 and ERR9825609, respectively) that are identical to them.

S1.9.24 XBE

In the Pango designation issue (https://github.com/cov-lineages/pango-designation/issues/1246), XBE was suggested as a recombinant between BA.5.2 (left parent; most of the genome) and BE.4 (right parent; somewhere from 23,609 onwards). However, sc2ts treats the 65 XBE samples as non-recombinant descendants of BA.5.2 because the number of supporting sites fell below our threshold of detection. We have included the BE.4 lineage on the subgraph, revealing two mutations (C27800T, and G28681T) which occur in parallel above the root of the XBE clade and along the path ascending from the displayed BE.4 sample, suggesting that if we used k=2 as the recombinant threshold, sc2ts would indeed detect XBE as a recombinant.

See github:sc2ts-paper/discussions/930 for further details.

S1.9.25 XBF

All the XBF samples descend from a well supported recombination node, with three mutations on the left of the breakpoint. Its left parent is a sample assigned to BA.5.2.1 (SRR20305553). Its right parent is a reconstructed internal node that is supported by a CJ.1-labelled sample (SRR21948068), which differs by three mutations.

S1.9.26 XBG

All the XBG samples descend from a well supported recombination node, with two mutations, one on each side of the breakpoint. Both its left and right parents are samples (SRR19920084 and ERR9852351, respectively).

S1.9.27 XBH

All the XBH samples descend from a well supported recombination node, despite two reversions (T222C and T12789C, respectively), which occur at excluded sites and were remapped by parsimony during postprocessing. If these two sites had been used during ARG inference, the branch attached to BA.2.1 would have been attached to the BA.2.5 shown on the top left instead, leading to a more parsimonious local topology with two mutations on the left branch. This would also remove the two blue lines on the left and side of the copying pattern.

S1.9.28 XBQ/XBK/XBK.1

XBK and XBQ are siblings, and neither appear to be recombinants in the ARG, as they are closely related to the non-recombinant node that originates CJ.1.3. At the time of designating XBK a hypothesis was made that a missing common ancestor could exist (https://github.com/

bioRxiv preprint doi: https://doi.org/10.1101/2023.06.08.544212; this version posted November 10, 2025. The copyright holder for this preprint (which was not certified by peer review) is the author/funder, who has granted bioRxiv a license to display the preprint in perpetuity. It is made available under aCC-BY 4.0 International license.

Table S6: Sc2ts detection results for Pango X lineages not added to the ARG. The number of samples in Viridian v04 dataset assigned to these Pango X lineages, the number of samples that passed sequence-level quality control, and the number of recombination breakpoints in the Viterbi solutions of the samples are shown.

Pango	Samples in Viridian	Samples passing QC	Breakpoints
XBJ	2	1	1
XBP	8	2	1
XBS	19	15	1
XBW	1	1	1
XCA	11	4	1
XAK	2	2	1
XAY	15	11	6
XBC	36	21	3
XBL	2	2	0
XBT	1	1	2

`cov-lineages/pango-designation/issues/1440#issuecomment-1355264973`), making XBK non-recombinant. Exactly such a missing ancestor is found as the upper internal node labelled CJ.1 in the ARG, constructed by sc2ts as part of its tree building steps.

See github:sc2ts-paper/discussions/931 for further details on XBQ.

See github:sc2ts-paper/discussions/932 for further details on XBK.

S1.9.29 XBM

All the XBM samples descend from a well supported recombination node. Its left parent is a reconstructed internal node that is directly supported by a BA.2.76-labelled sample identical to it. Its right parent is a BF.3-labelled sample.

S1.9.30 XBR

This one XBR sample descends from a well supported recombination node. Its left parent is a BN.3.1-labeled sample, and its right parent is a reconstructed internal node that is directly supported by a BQ.1.25-labeled sample identical to it.

S1.10 Pango X lineages not added to the ARG

Ten Pango X lineages which are present in the Viridian v04 dataset were not integrated into the ARG due to high HMM costs and/or insufficient sample support, but their samples were matched against the ARG during inference. Table S6 summarizes the HMM matching results of these Pango X lineages. All were identified as recombinants by sc2ts, except XBL. (For XBL, the evidence for recombination proposed in the Pango designation issue[160] involves only three informative sites supporting the suggested rightmost parent, and therefore this likely fell below our threshold of detection.) Importantly, these results demonstrate that sc2ts can detect recombinants with multiple breakpoints (XAY, XBC, and XBT). XBC and XBT are relatively clean examples of multiple-breakpoint recombinants, with the Pango lineages of the parents and the breakpoint intervals concordant with those suggested in the Pango designation issues, but XAY is more complex, possibly involving up to six breakpoints as per sc2ts.

bioRxiv preprint doi: https://doi.org/10.1101/2023.06.08.544212; this version posted November 10, 2025. The copyright holder for this preprint (which was not certified by peer review) is the author/funder, who has granted bioRxiv a license to display the preprint in perpetuity. It is made available under aCC-BY 4.0 International license.

Table S7: Distribution of numbers of descendants for UShER+RIPPLES events and sc2ts. The first column shows the number of descendants (binned for larger values) and the others show the corresponding number of events.

Count	RIPPLES p3	RIPPLES p4	sc2ts
1	2616	757	525
2	575	178	124
3	232	65	47
4	124	38	32
5	74	14	17
5-100	437	108	96
100-1000	32	6	8
1000-10k	15	6	4
10k-100k	2	0	0
100k-1m	4	2	2

S1.11 Detailed RIPPLES analysis

We ran RIPPLES and aligned events with the sc2ts ARG as described in STAR Methods. We begin by describing the overall composition of events output by RIPPLES at the two parameter values used.

At $p = 4$, RIPPLES returned a total of 1174 events, and 610 pass 3SEQ validation (51%). 1052 events (89.6%) were in close clade agreement with the sc2ts ARG, and 468 (40%) corresponded to recombination events in the ARG (Figure S18A). We investigated the 701 RIPPLES events that could be aligned with the ARG but were not close to a recombination node to determine why they were not found by sc2ts. For 408 of these RIPPLES identified recombination events, one or more parents were *younger* than the recombinant sequence based on metadata, and therefore impossible under the strict time ordering enforced by sc2ts. Secondly, of the 179 remaining events with matching clades and in which the sc2ts node has more than 4 mutations, of the total 1,167 mutations, 452 of these occurred at sites that were remapped during post-hoc parsimony (and thus the mutations were not observed by the LS HMM during primary inference). The remaining "missing" events are likely attributable to the finer details of the differences in tree building approaches between UShER and sc2ts.

At $p = 3$, RIPPLES returned a total of 4111 events, and 2718 pass 3SEQ validation (66%). 3755 events (91.3%) were in close clade agreement with the sc2ts ARG, and 595 (14%) corresponded to recombination events in the ARG (Figure S18B). We did not investigate the $p = 3$ events any further as the additional events detected at the lower threshold are not expected to be detected by sc2ts at the parameter value used ($k = 4$).

Next we considered the overall distribution of the numbers of descendants of the events returned by sc2ts and RIPPLES (Table S7). Overall, the distributions were similar, with the majority having very few descendants and being dominated by singletons. The events subtending $>$ 100,000 samples in all three cases are noteworthy (see Document S1.12 for analysis of the two sc2ts events).

We next examined the RIPPLES events referencing Pango X lineages. Table S8 shows a summary of all recombination events associated with Pango X lineages detected by RIPPLES at $p = 3$ and their relationship with the sc2ts ARG. Of the 16 Class I events (X lineages unequivocally associated with a recombination node in the sc2ts ARG) in Table 1, 8 have corresponding recombination events (4, at $p = 4$). Of the 5 Type II events (recombination nodes with more complex assocation with X lineages), two have close matches. XBM matches exactly (including

bioRxiv preprint doi: https://doi.org/10.1101/2023.06.08.544212; this version posted November 10, 2025. The copyright holder for this preprint (which was not certified by peer review) is the author/funder, who has granted bioRxiv a license to display the preprint in perpetuity. It is made available under aCC-BY 4.0 International license.

Table S8: RIPPLES events associated with Pango X lineages. Each row is a recombination event identified by RIPPLEs (at $p = 3$) in which Pango X lineages appear, along with the corresponding sc2ts event from Table 1, where applicable, grouped by class. Events also present at $p = 4$ are indicated. Clades are matched by finding the most recent common ancestor of the samples associated with each UShER+RIPPLES event in the sc2ts ARG. The clade differences indicates the closeness of the match.

sc2ts event	in $p = 4$	UShER desc	sc2ts class	clade diff	descendants
Equivalent Sc2ts and RIPPLES recombination events					
XC	✓	5	I	0	XC:5
XBR	✓	1	I	0	XBR:1
XS	✓	17	I	0	XS:17
XA	✓	39	I	0	XA:39
XY		23	I	0	XY:23
XW		32	I	0	XW:32
XL		64	I	0	XL:64
XBB		6452	I	1	XBB:6452
RIPPLEs recombination event close to sc2ts recombination event					
XBM	✓	12	II	0	XBM:10, BF.3:2
XM	✓	8	II	0	XM:4, BA.2:4
Xx	✓	18	II	0	XAC:18
Xx		9	II	0	XAE:9
XQ		1	II	0	XAM:1
XQ		21	II	0	XAM:21
XE/XH		1113	II	3	XE:1113
RIPPLEs recombination event equivalant to sc2ts non-recombination event					
XB	✓	411	IV	0	XB:192, B.1:219
RIPPLEs recombination event not comparable to sc2ts					
NA	✓	19	NA	45190	XAJ:18, BA.4:1
NA	✓	35	NA	7147	XBD:30, BA.2.75:5
NA	✓	36	NA	7146	XBR:1, XBD:30, BA.2.75:5
NA		20	NA	1061775	XS:17, BA.1:1, BA.1.1:1, BA.1.15:1
NA		1321	NA	340501	XE:1113, XJ:68, BA.2:82, XM:29, XY:23, XAL:3, XAF:1, XH:2

bioRxiv preprint doi: https://doi.org/10.1101/2023.06.08.544212; this version posted November 10, 2025. The copyright holder for this preprint (which was not certified by peer review) is the author/funder, who has granted bioRxiv a license to display the preprint in perpetuity. It is made available under aCC-BY 4.0 International license.

2 descending BF.3 samples), and XE/XH is similar (but without including the 2 XH samples in the clade). UShER and sc2ts agree on creating two separate XAM clades, but while sc2ts makes these both descendants of the Xx event, RIPPLES creates two independent recombinations. Similarly, XAC and XAE have exactly matching clades, but RIPPLES infers two independent recombination events while sc2ts groups them both under the Xx event. XB is notable as a likely sc2ts false negative that is identified by RIPPLES at both $p = 3$ and $p = 4$. The remaining events associated with X lineages output by RIPPLES cannot be easily mapped to the sc2ts ARG as one or more descendants are distantly placed. XAJ is an outlier for sc2ts, being the Pango X lineage identified as a non-recombinant with the largest number of mutations (Table 1), and a likely false negative. The remaining 4 events are mixtures of multiple lineages that are difficult to reconcile with either the other events in the table or the sc2ts ARG.

See notebook analysis_ripples.ipynb for details.

S1.12 Likely spurious recombinants with many descendants

In the sc2ts ARG, there are two recombination events which have a large number of descendants. The first of these corresponds to recombination event within BA.2 whose breakpoint is at position 27382 on the far right of the genome. Although supported by 5 sites, three of them are adjacent so that they only represent 3 loci, meaning that this event does not pass our QC filter..

The second of these involves a single breakpoint at position 26858, and is the origin of the BA.5 lineage in the ARG. Although supported by 5 loci on the right, this recombinant also requires 3 de-novo mutations, some of which are adjacent to the supporting loci. Furthermore, when the first BA.5 sample is added to the ARG, it is possible to construct a more parsimonious non-recombinant origin by long branch splitting, although subsequent tree building does not make this a parsimonious way to rewire the final ARG. It is therefore likely to be spurious.

bioRxiv preprint doi: https://doi.org/10.1101/2023.06.08.544212; this version posted November 10, 2025. The copyright holder for this preprint (which was not certified by peer review) is the author/funder, who has granted bioRxiv a license to display the preprint in perpetuity. It is made available under aCC-BY 4.0 International license.

Supplementary Figures

bioRxiv preprint doi: https://doi.org/10.1101/2023.06.08.544212; this version posted November 10, 2025. The copyright holder for this preprint (which was not certified by peer review) is the author/funder, who has granted bioRxiv a license to display the preprint in perpetuity. It is made available under aCC-BY 4.0 International license.

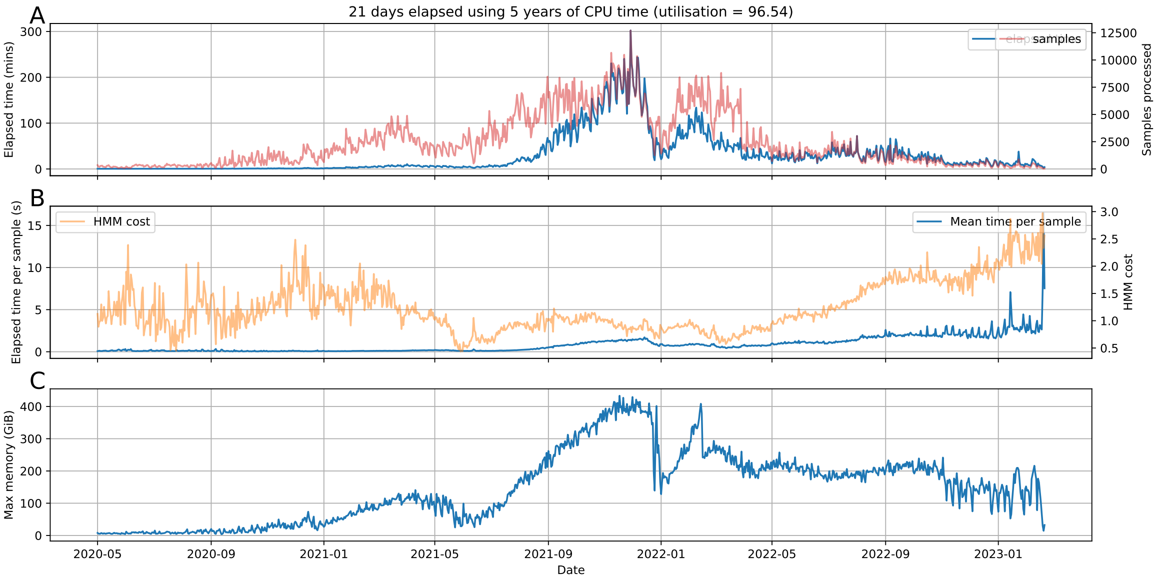

Figure S1: Computational resources used during the ARG inference process. (A) The amount of time to process a daily batch of samples and the number of samples processed per day. (B) Mean time to process a sample is less than 5s except when the number of samples is small. (C) RAM usage is the most difficult resource to manage, and depends on the sample composition. The sharp drop in 2022-01 corresponds to the sweep in which Omicron BA.1 replaced Delta and there was very little sample diversity. The drop in RAM usage around 2022-02 corresponds to a change in configuration where we reduced the number of HMM threads from 128 to 80 to better fit into available memory.

bioRxiv preprint doi: https://doi.org/10.1101/2023.06.08.544212; this version posted November 10, 2025. The copyright holder for this preprint (which was not certified by peer review) is the author/funder, who has granted bioRxiv a license to display the preprint in perpetuity. It is made available under aCC-BY 4.0 International license.

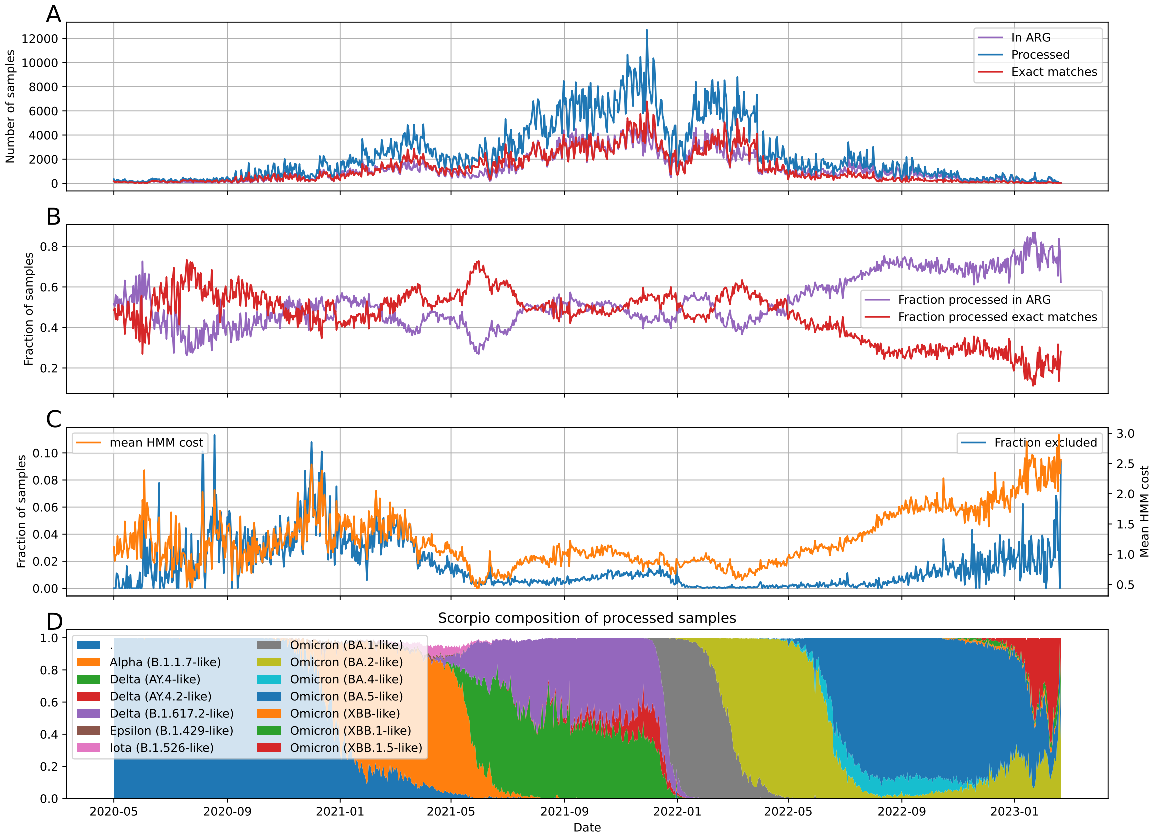

Figure S2: Daily statistics of samples analysed during the ARG inference process. (A) The number of samples processed from the input sequence alignments, exactly matched to a node in the current ARG, and added to the ARG per day. (B) The fraction of exactly matched samples and the fraction of samples added to the ARG mirror each other. The fraction of exact matches is a function of both sampling depth and composition of the global population. (C) The mean HMM cost per sample is a useful metric. (D) Proportion of samples classified by (simplified) Scorpio designation.

bioRxiv preprint doi: https://doi.org/10.1101/2023.06.08.544212; this version posted November 10, 2025. The copyright holder for this preprint (which was not certified by peer review) is the author/funder, who has granted bioRxiv a license to display the preprint in perpetuity. It is made available under aCC-BY 4.0 International license.

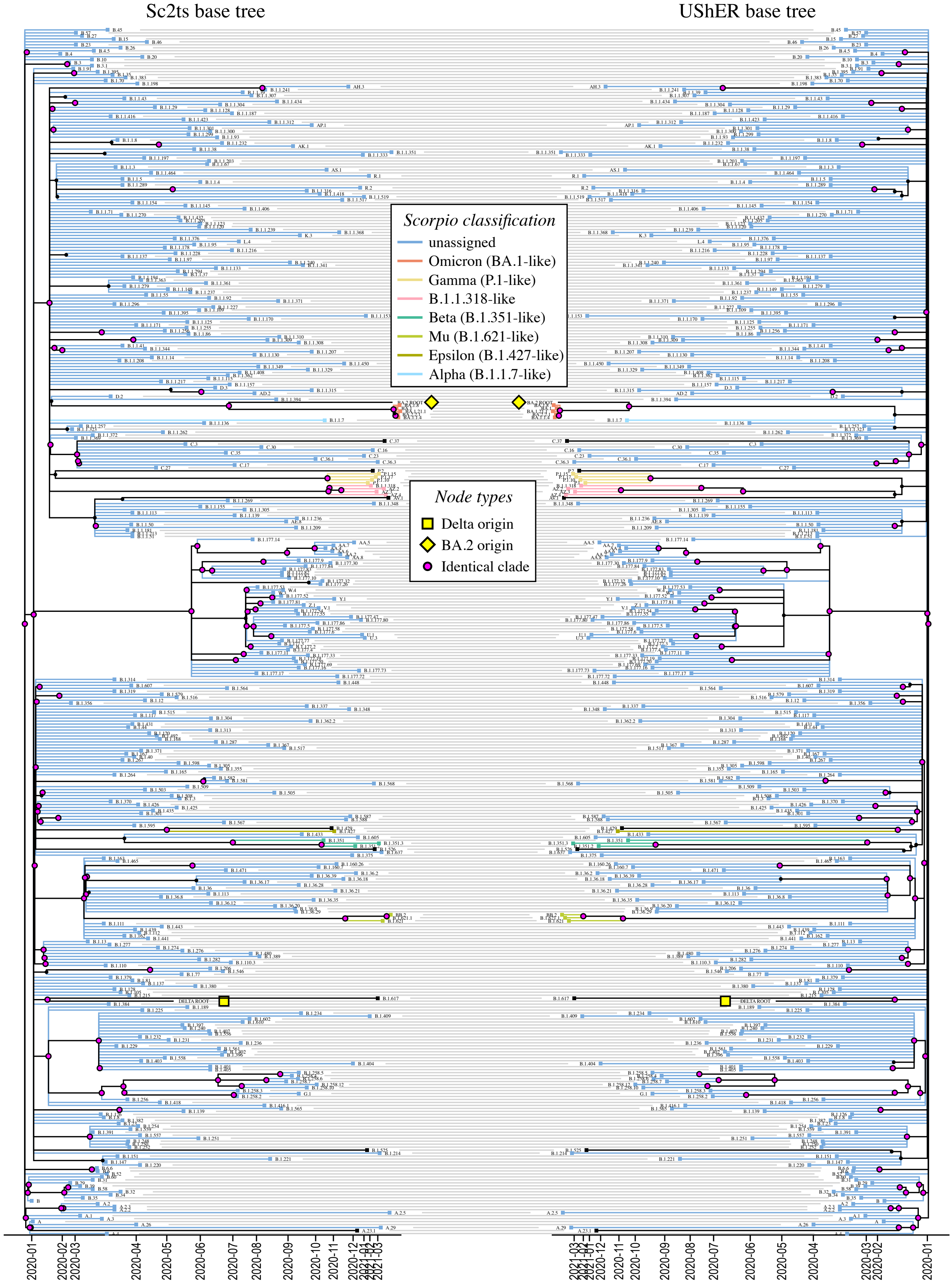

Figure S3: Tanglegram comparing the basic phylogenetic backbones of sc2ts and UShER.

bioRxiv preprint doi: https://doi.org/10.1101/2023.06.08.544212; this version posted November 10, 2025. The copyright holder for this preprint (which was not certified by peer review) is the author/funder, who has granted bioRxiv a license to display the preprint in perpetuity. It is made available under aCC-BY 4.0 International license.

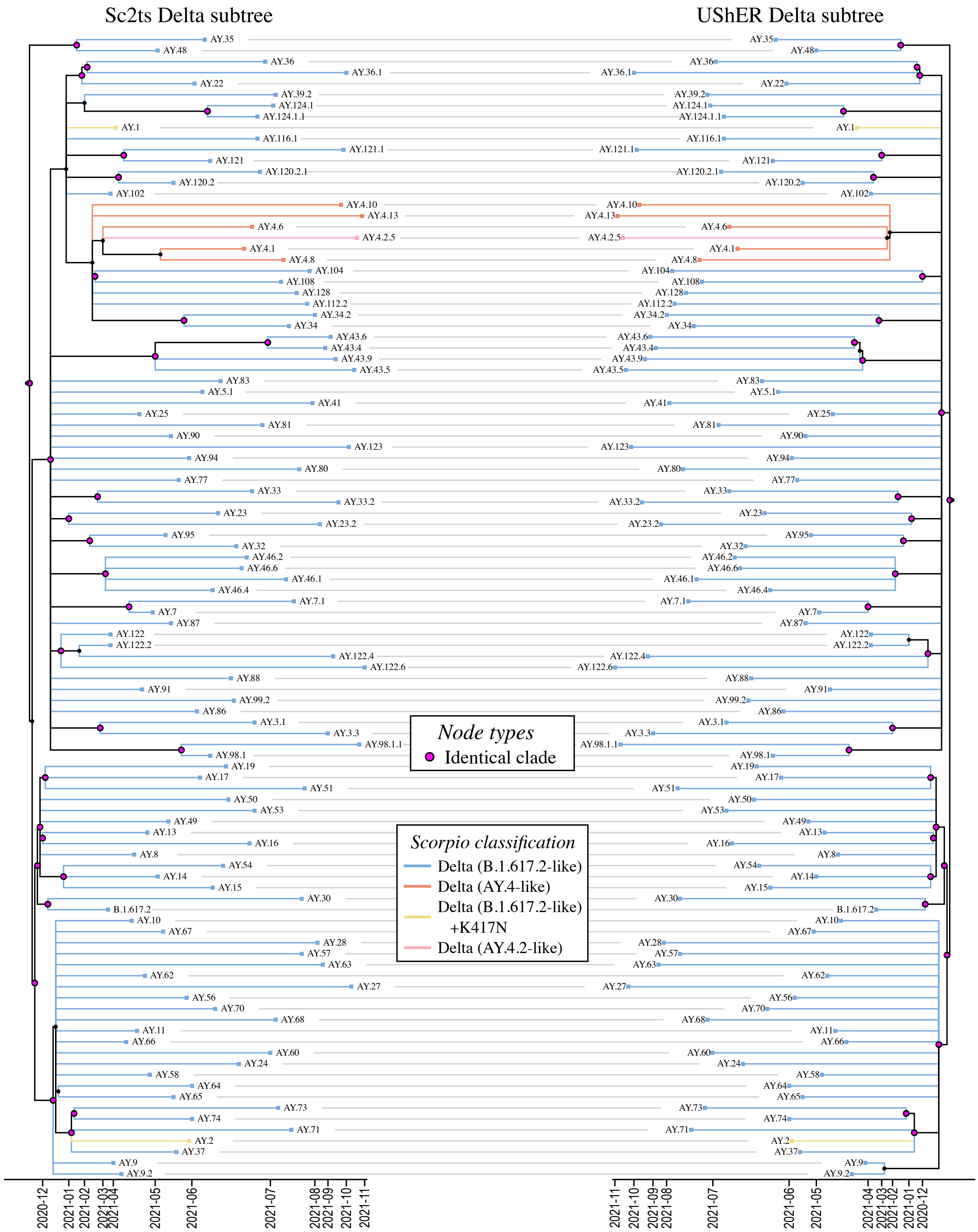

Figure S4: Tanglegram comparing sc2ts and UShER on the Delta subtree.

bioRxiv preprint doi: https://doi.org/10.1101/2023.06.08.544212; this version posted November 10, 2025. The copyright holder for this preprint (which was not certified by peer review) is the author/funder, who has granted bioRxiv a license to display the preprint in perpetuity. It is made available under aCC-BY 4.0 International license.

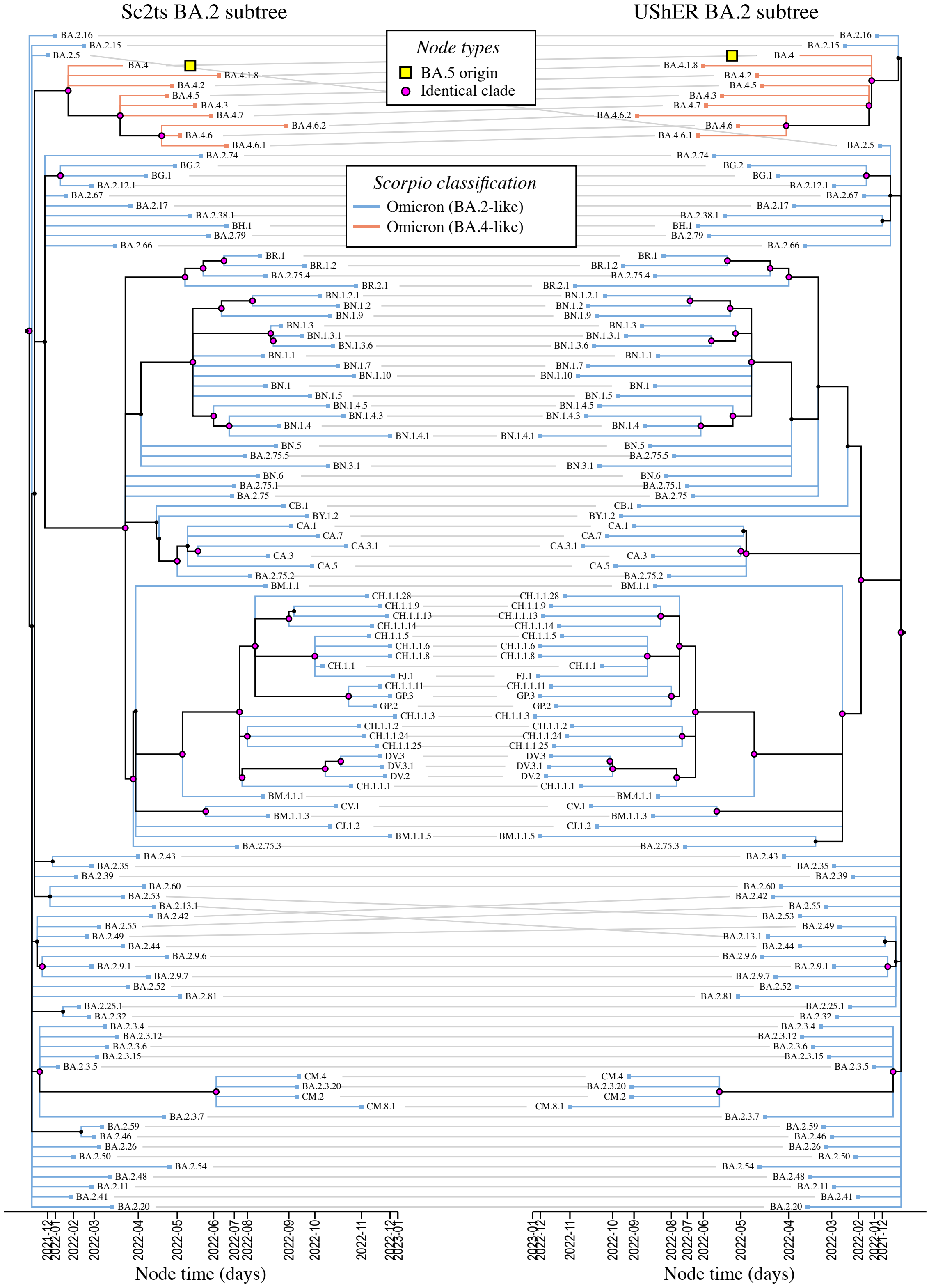

Figure S5: Tanglegram comparing sc2ts and UShER on the BA.2 subtree.

bioRxiv preprint doi: https://doi.org/10.1101/2023.06.08.544212; this version posted November 10, 2025. The copyright holder for this preprint (which was not certified by peer review) is the author/funder, who has granted bioRxiv a license to display the preprint in perpetuity. It is made available under aCC-BY 4.0 International license.

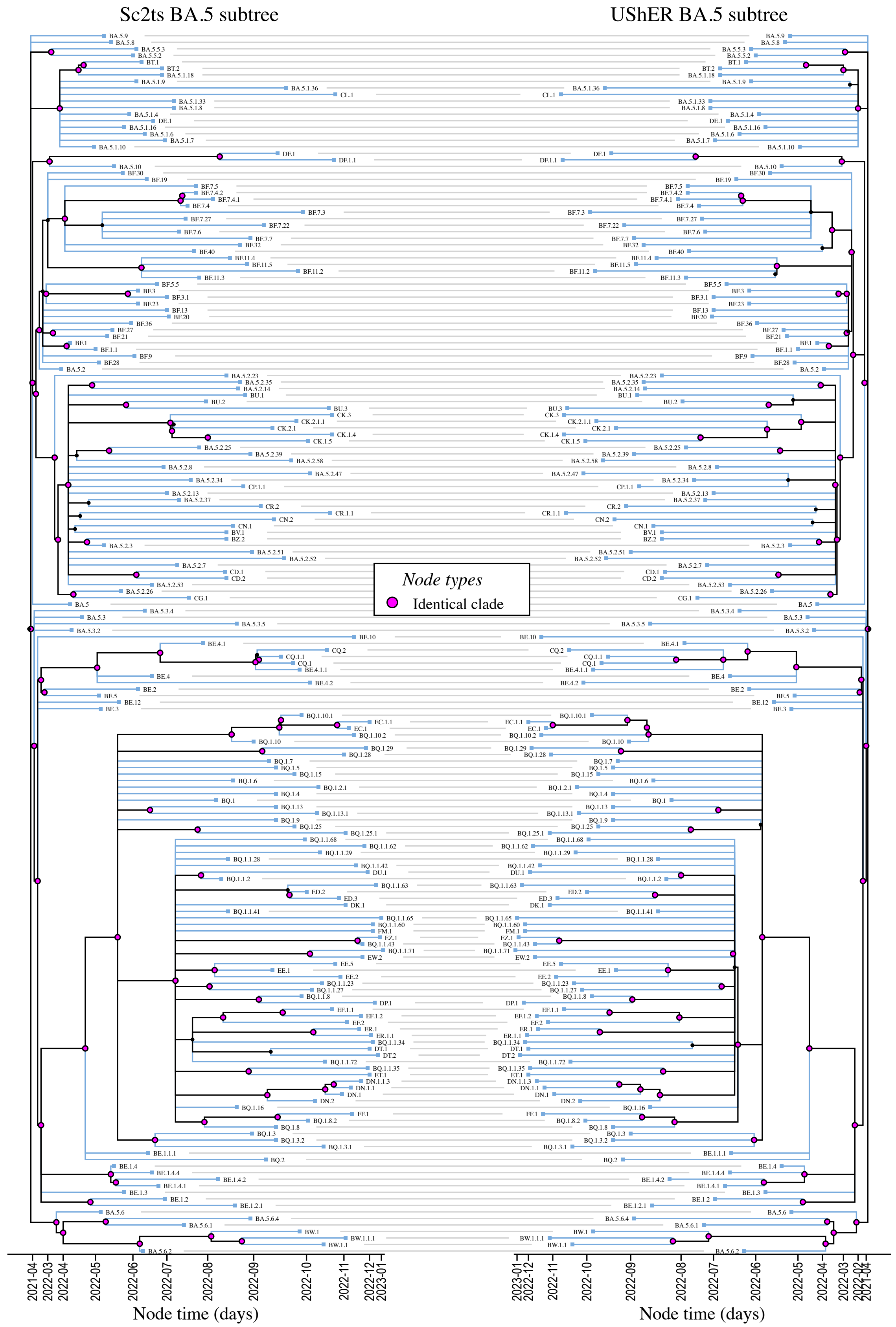

Figure S6: Tanglegram comparing sc2ts and UShER on the BA.5 subtree.

bioRxiv preprint doi: https://doi.org/10.1101/2023.06.08.544212; this version posted November 10, 2025. The copyright holder for this preprint (which was not certified by peer review) is the author/funder, who has granted bioRxiv a license to display the preprint in perpetuity. It is made available under aCC-BY 4.0 International license.

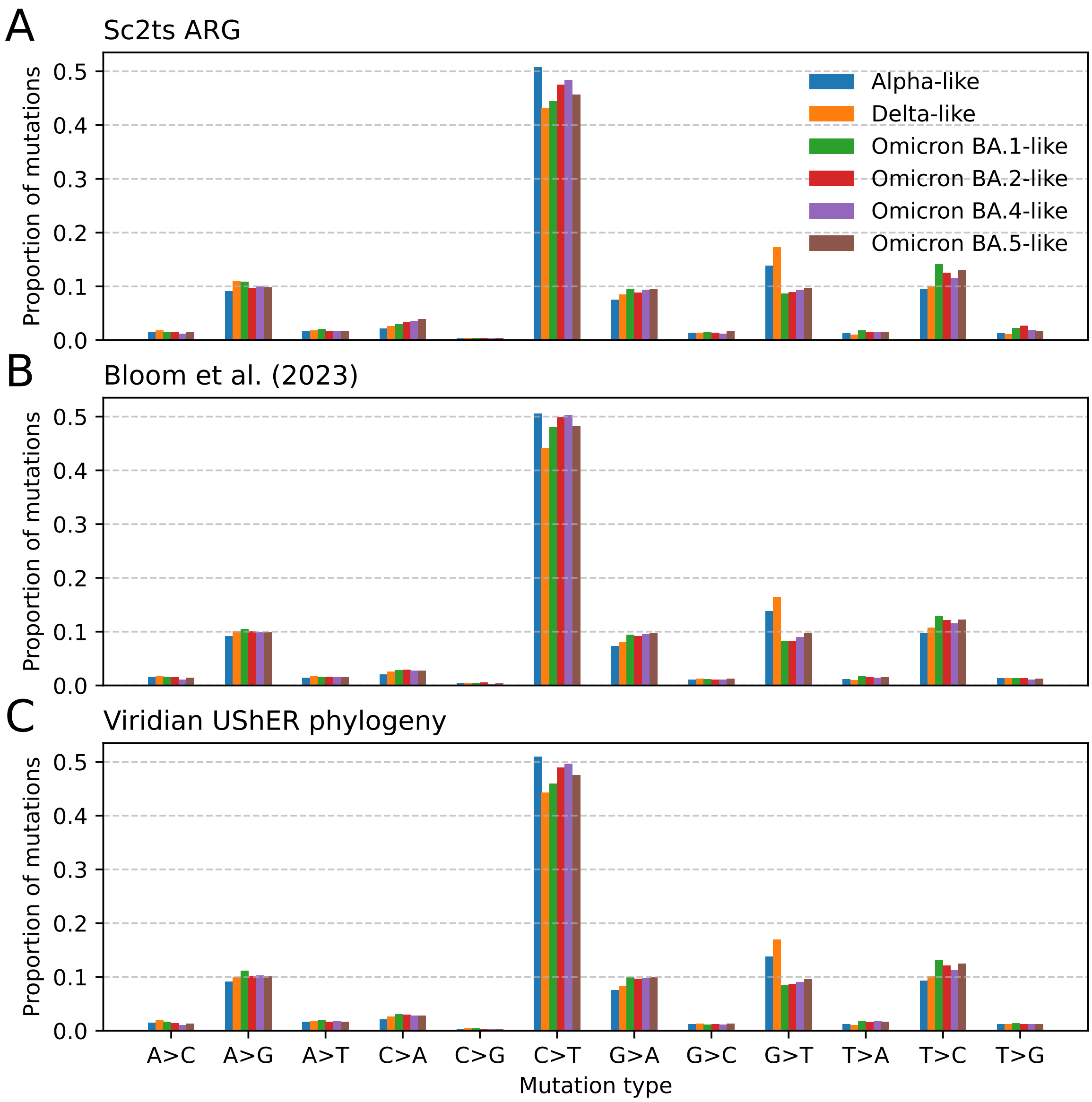

Figure S7: All-site mutational spectra of major VOCs calculated from the sc2ts ARG (A), the mutation count data from Bloom et al. (2023)[78] (B), and the Viridian UShER phylogeny from Hunt et al. (2024)[76].

bioRxiv preprint doi: https://doi.org/10.1101/2023.06.08.544212; this version posted November 10, 2025. The copyright holder for this preprint (which was not certified by peer review) is the author/funder, who has granted bioRxiv a license to display the preprint in perpetuity. It is made available under aCC-BY 4.0 International license.

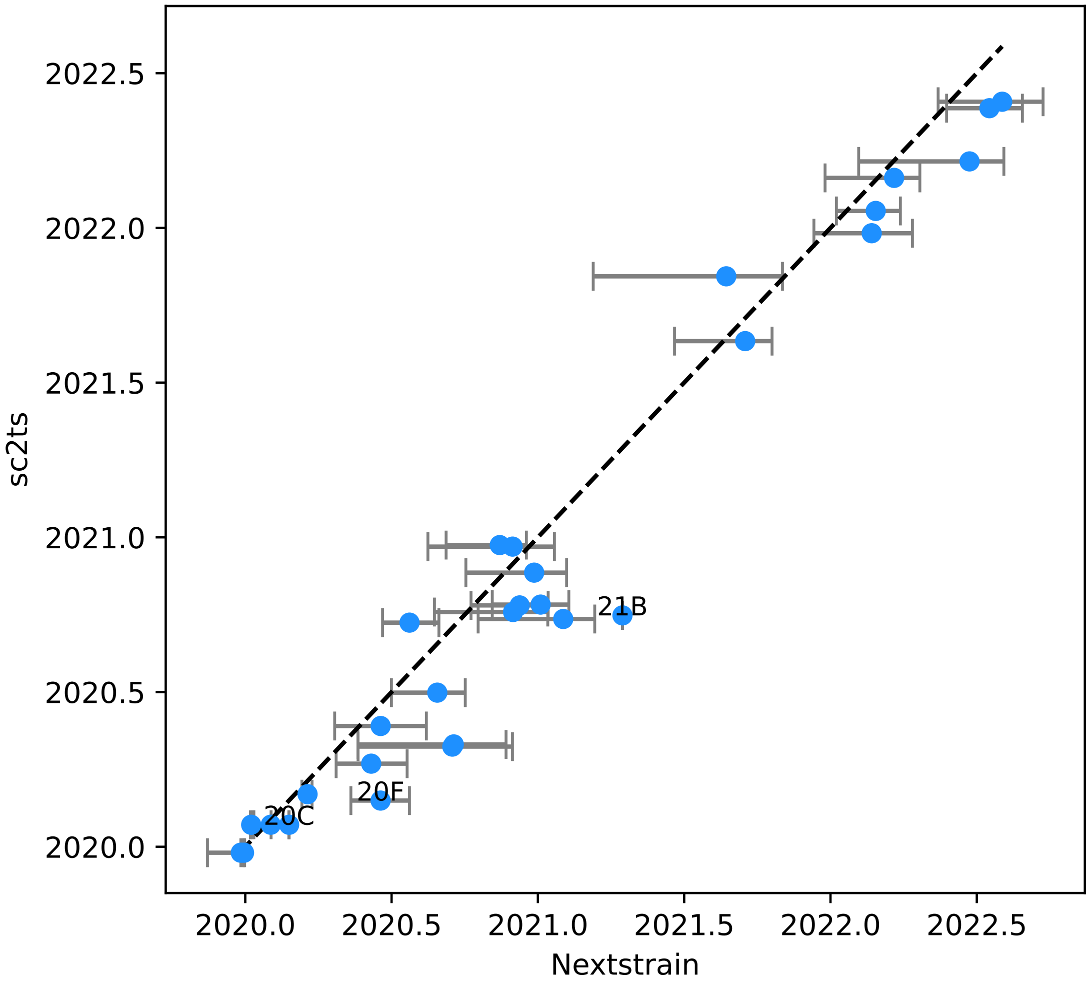

Figure S8: Inferred times of internal nodes corresponding to origins of Nextclade clades (y-axis) against those estimated in the Nextclade tree (x-axis). Confidence intervals shown as horizontal bars. Clades are labelled where dates differ by more than 28 days.

bioRxiv preprint doi: https://doi.org/10.1101/2023.06.08.544212; this version posted November 10, 2025. The copyright holder for this preprint (which was not certified by peer review) is the author/funder, who has granted bioRxiv a license to display the preprint in perpetuity. It is made available under aCC-BY 4.0 International license.

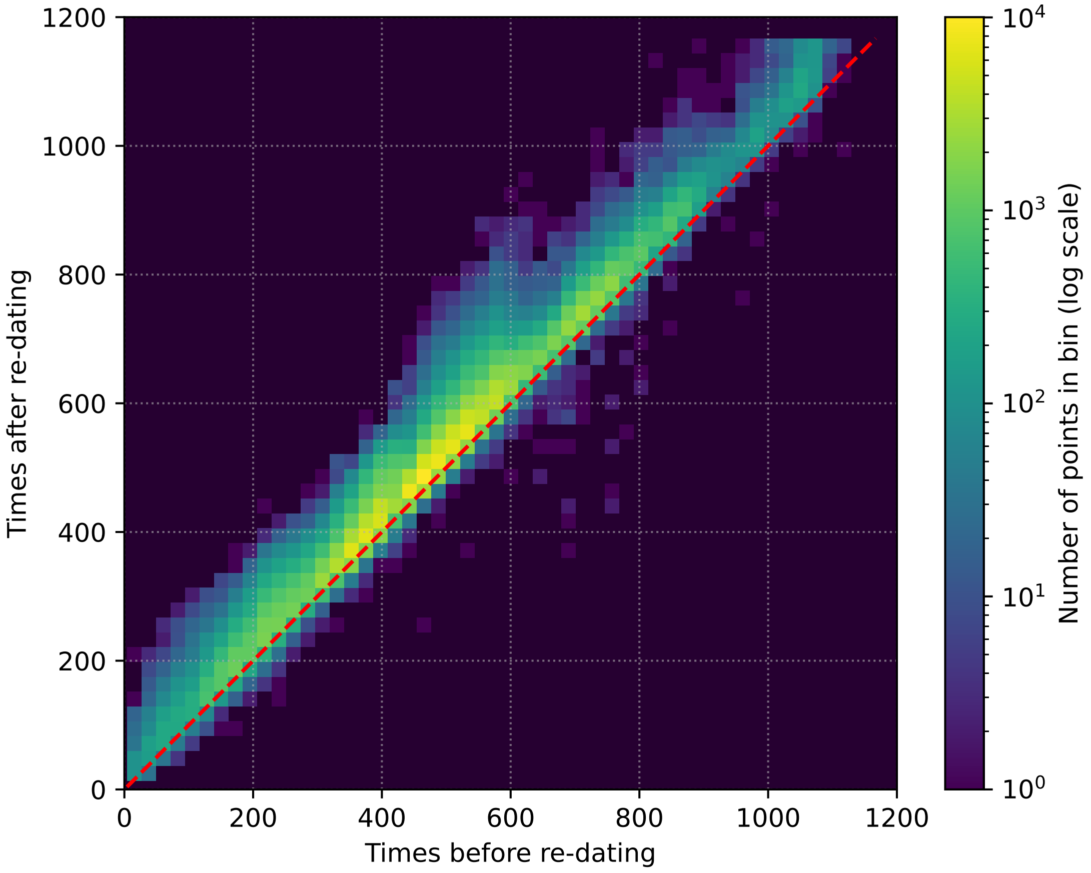

Figure S9: Heat map showing differences (in days) between actual and re-estimated sample dates for a subset of internal sample nodes.

bioRxiv preprint doi: https://doi.org/10.1101/2023.06.08.544212; this version posted November 10, 2025. The copyright holder for this preprint (which was not certified by peer review) is the author/funder, who has granted bioRxiv a license to display the preprint in perpetuity. It is made available under aCC-BY 4.0 International license.

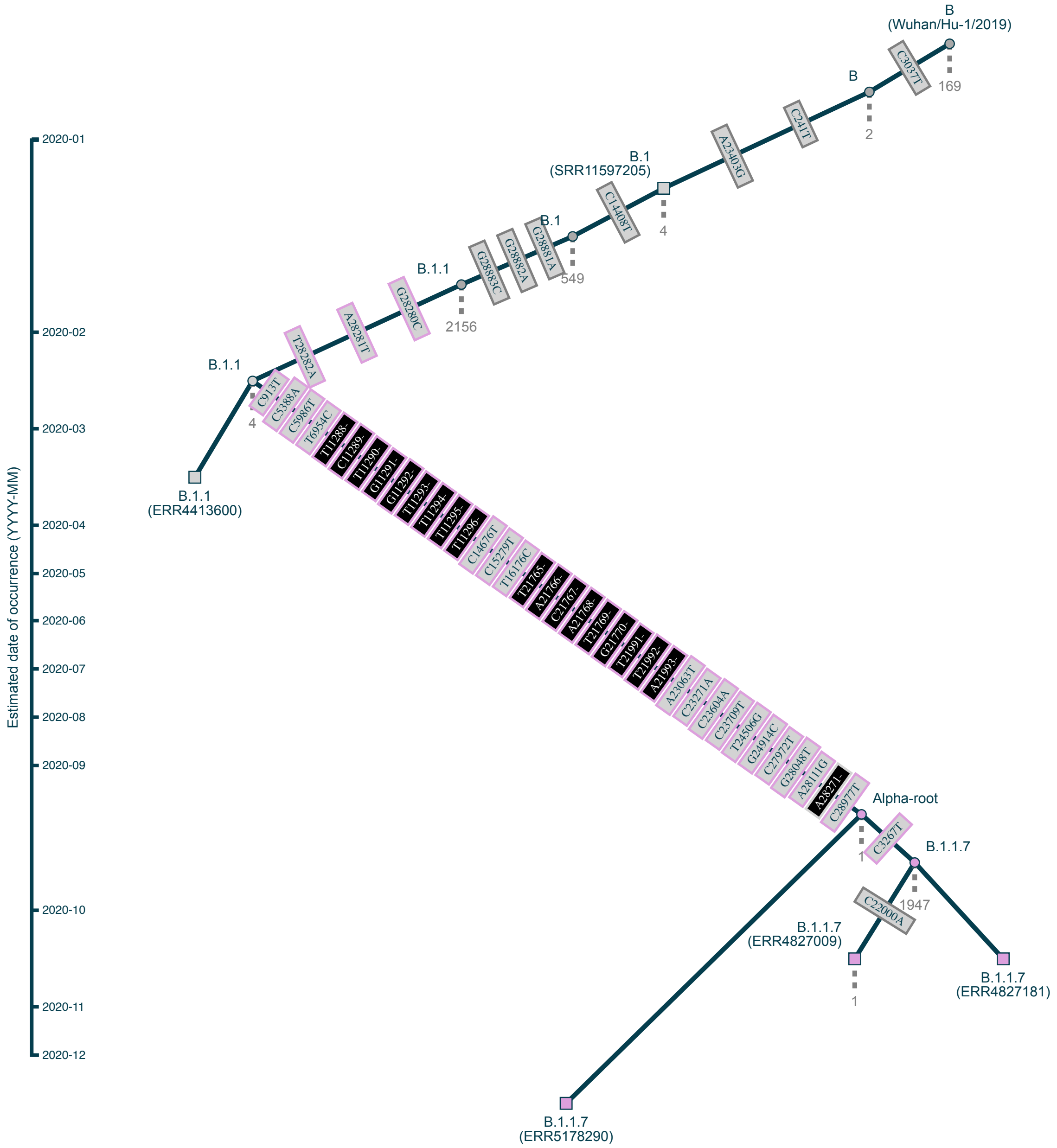

Figure S10: Subgraph illustrating the saltational origin of B.1.1.7 (Alpha). Squares represent sample nodes, circles represent reconstructed internal nodes; nodes classified as B.1.1.7 by Pangolin v4.3.1 are in pink. Mutations are shown as rectangles along branches, ordered by position; deletions are black. Characteristic mutations associated with the emergence of Alpha are highlighted with a pink outline.

bioRxiv preprint doi: https://doi.org/10.1101/2023.06.08.544212; this version posted November 10, 2025. The copyright holder for this preprint (which was not certified by peer review) is the author/funder, who has granted bioRxiv a license to display the preprint in perpetuity. It is made available under aCC-BY 4.0 International license.

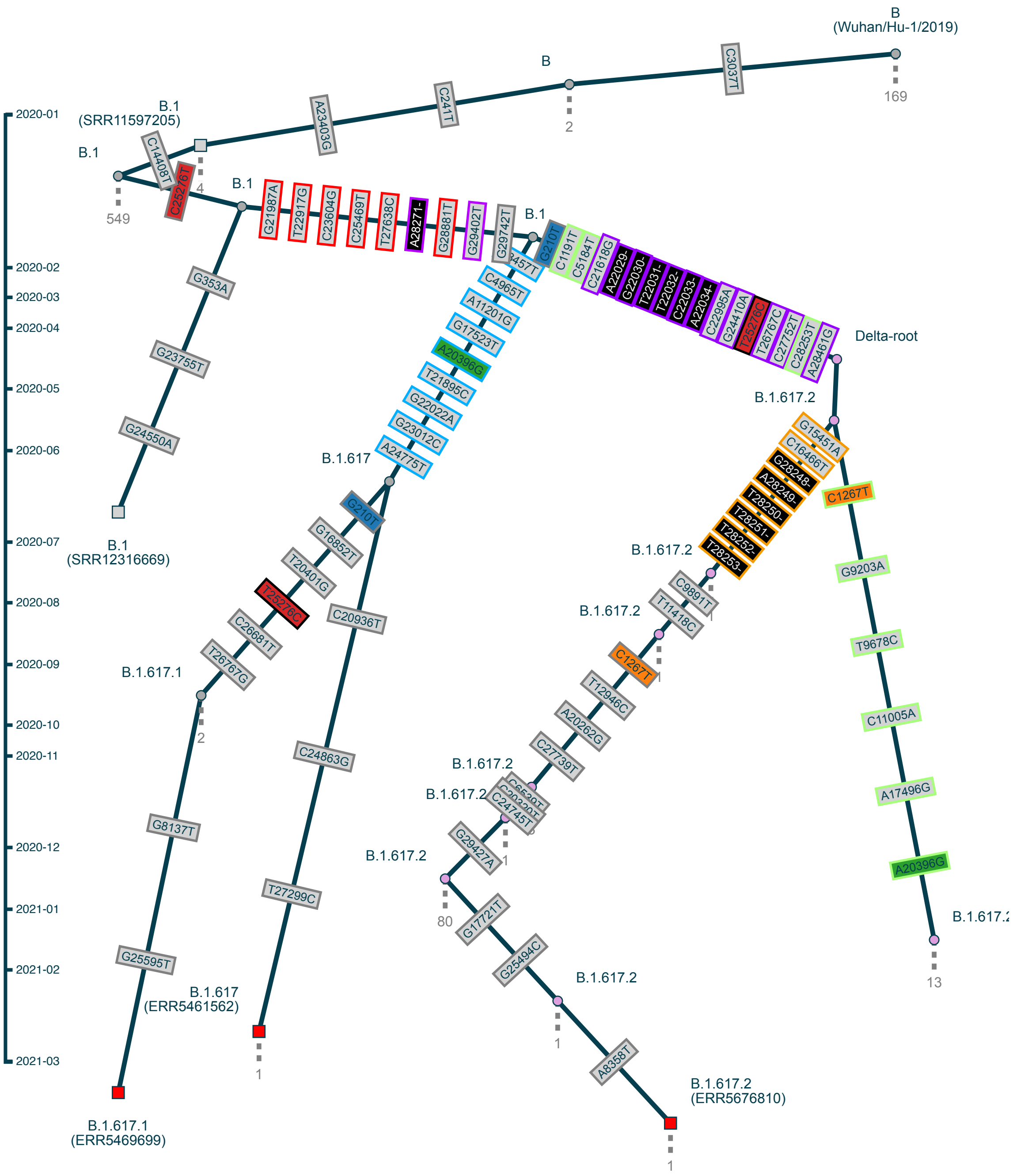

Figure S11: Subgraph illustrating the origin of B.1.617.2 (Delta). Symbols are described in S10, except that we highlight the characteristic mutations of Kappa and Delta: those with a blue outline are listed in the Pango designation issue[161] or the Kappa constellation[162], but absent in the Delta constellation; those with a purple outline are listed in the Delta constellation[163] or are Delta-specific deletions reported in Stern et al.[99], but absent in the Kappa constellation; and those with a red outline are listed in both the Kappa and Delta constellations. We also outline in orange the mutations associated with the lineage containing clades A to D and in green those associated with lineage containing clade E. Sites associated with multiple mutations in the subgraph have their mutations assigned a unique fill colour (orange, blue, green, red, etc); reversions are further highlighted with a black outline. Sc2ts seed samples (see STAR methods) for B.1.617, B.1.617.1, and B.1.617.2 are plotted as red squares.

bioRxiv preprint doi: https://doi.org/10.1101/2023.06.08.544212; this version posted November 10, 2025. The copyright holder for this preprint (which was not certified by peer review) is the author/funder, who has granted bioRxiv a license to display the preprint in perpetuity. It is made available under aCC-BY 4.0 International license.

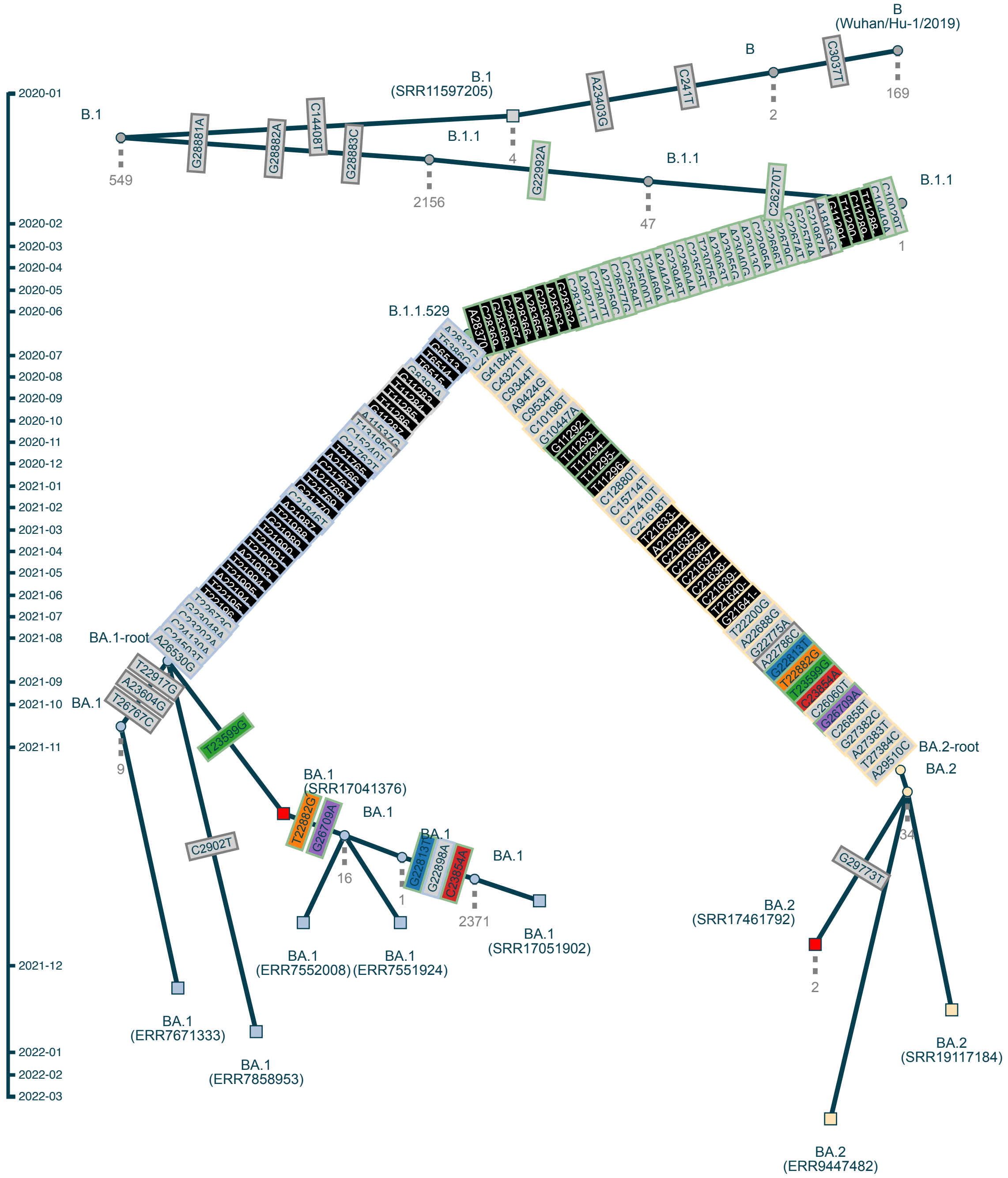

Figure S12: Subgraph illustrating the saltational origin of the major Omicron lineages BA.1 and BA.2. Symbols are described in Figure S11, except that BA.1 samples are filled in light blue (also used to outline BA.1 characteristic mutations), BA.2 samples in light yellow (also used to outline BA.2 characteristic mutations), and B.1.1.529 mutations which are neither BA.1 or BA.2 characteristic mutations are outlined in green. These colours correspond to those used in the BA.1/BA.2 Pango designation issue[101].

bioRxiv preprint doi: https://doi.org/10.1101/2023.06.08.544212; this version posted November 10, 2025. The copyright holder for this preprint (which was not certified by peer review) is the author/funder, who has granted bioRxiv a license to display the preprint in perpetuity. It is made available under aCC-BY 4.0 International license.

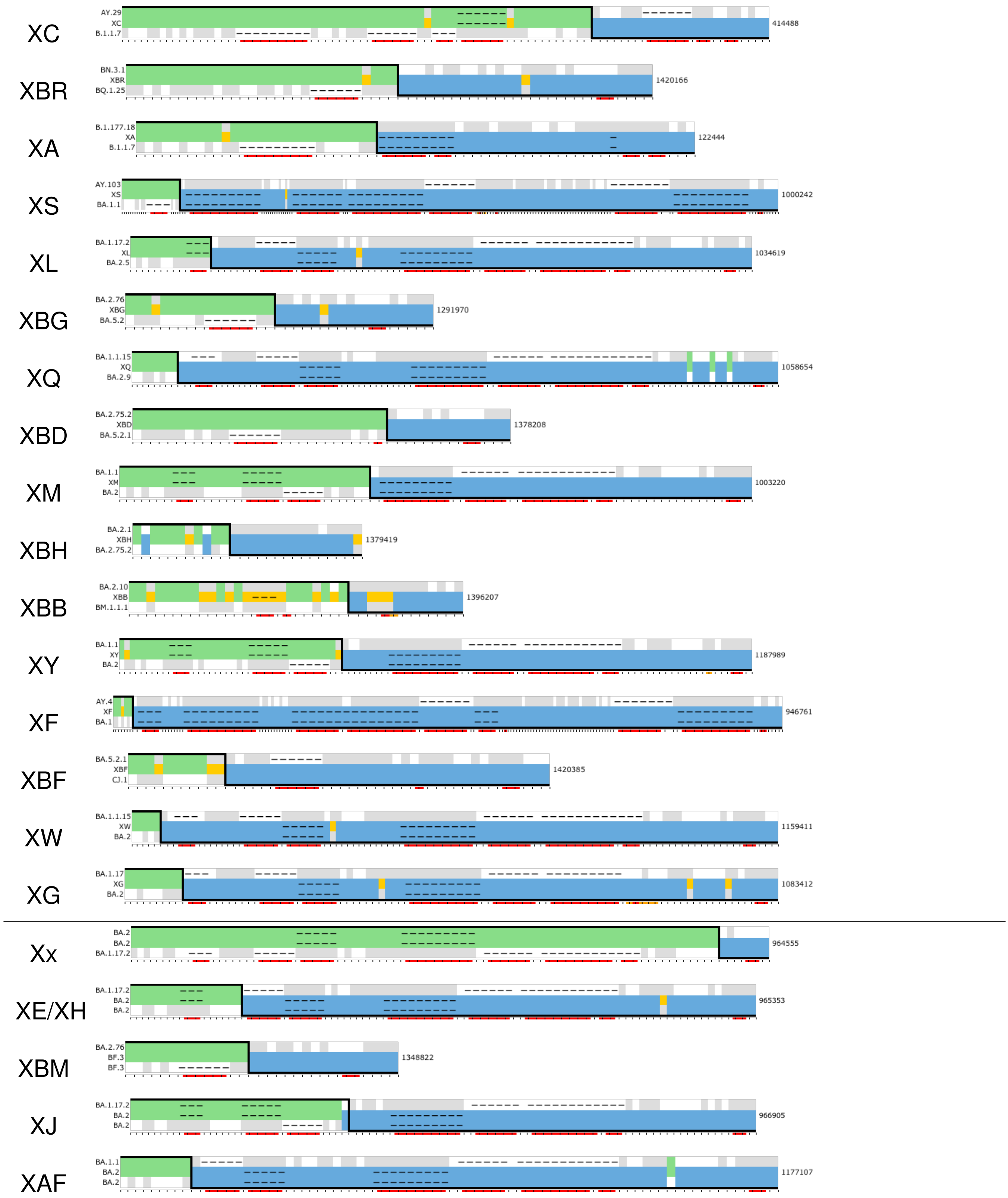

Figure S13: Copying patterns for the 21 recombination events associated with Pango X lineages listed in Table 1 (in the same order). The horizontal line separates Type I and Type II events. See also Document S3 for exact positions and nucleotide bases.

bioRxiv preprint doi: https://doi.org/10.1101/2023.06.08.544212; this version posted November 10, 2025. The copyright holder for this preprint (which was not certified by peer review) is the author/funder, who has granted bioRxiv a license to display the preprint in perpetuity. It is made available under aCC-BY 4.0 International license.

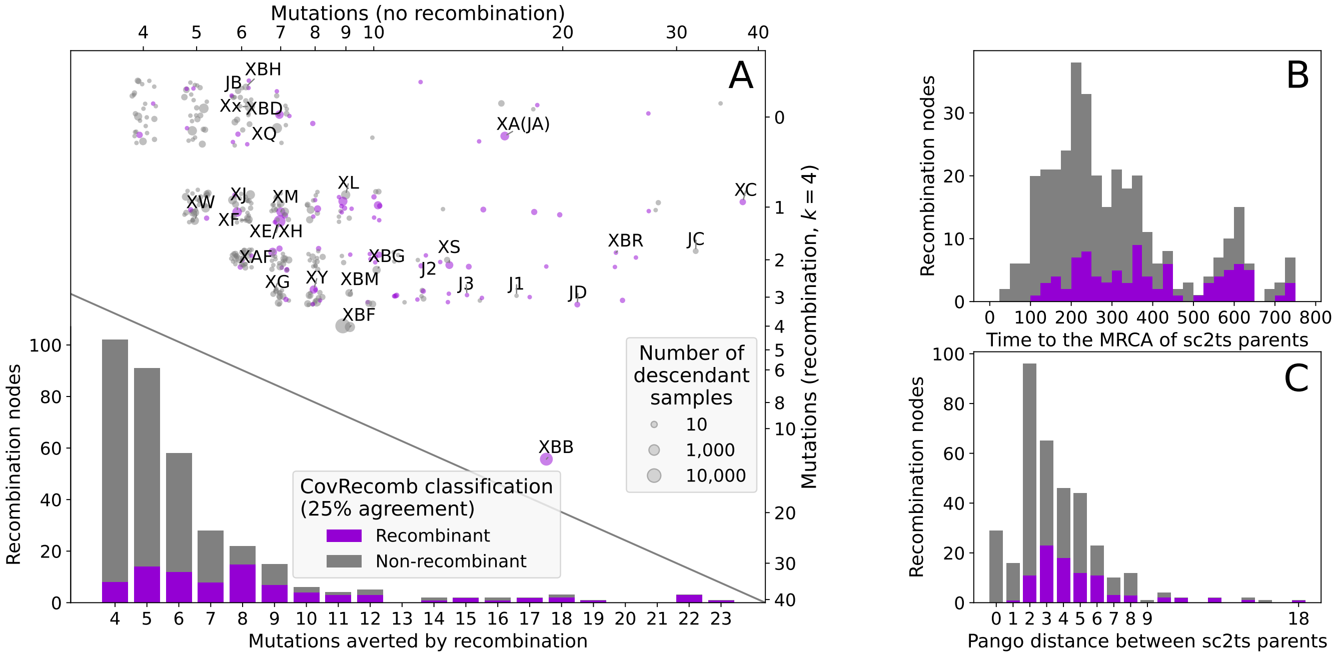

Figure S14: Properties of sc2ts recombination events, coloured by CovRecomb classification. All other details as per Figure 3.

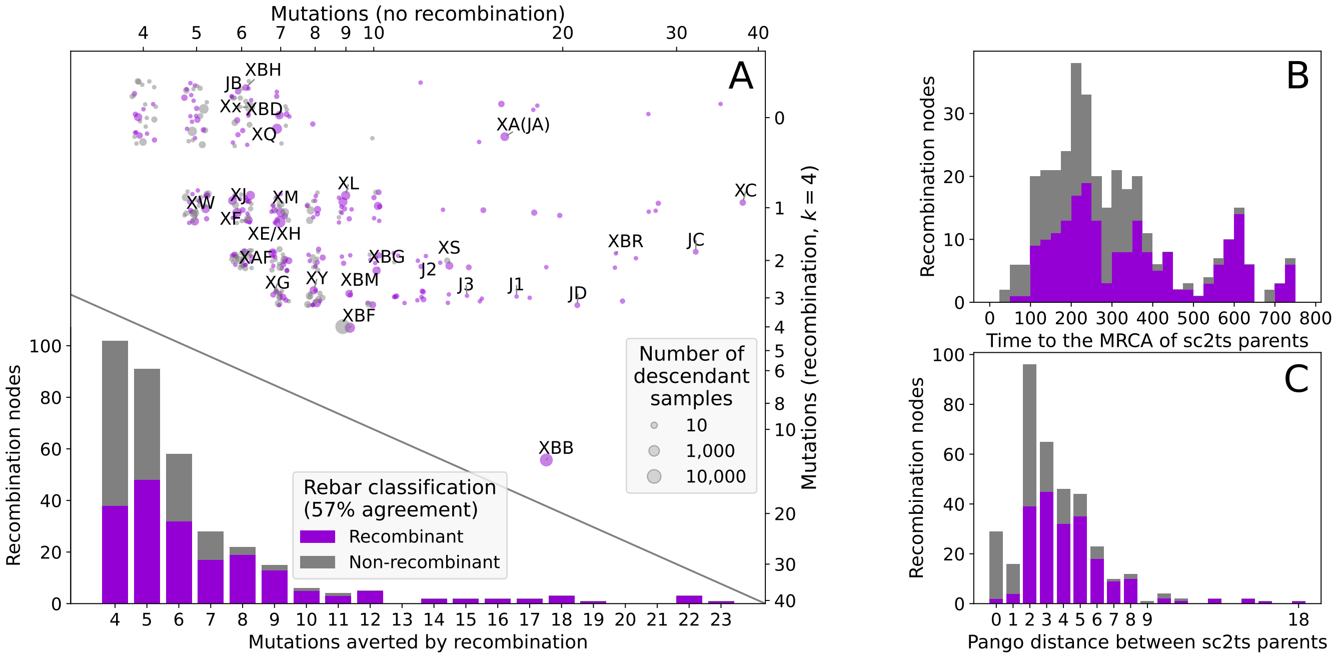

Figure S15: Properties of sc2ts recombination events, coloured by rebar classification. All other details as per Figure 3.

bioRxiv preprint doi: https://doi.org/10.1101/2023.06.08.544212; this version posted November 10, 2025. The copyright holder for this preprint (which was not certified by peer review) is the author/funder, who has granted bioRxiv a license to display the preprint in perpetuity. It is made available under aCC-BY 4.0 International license.

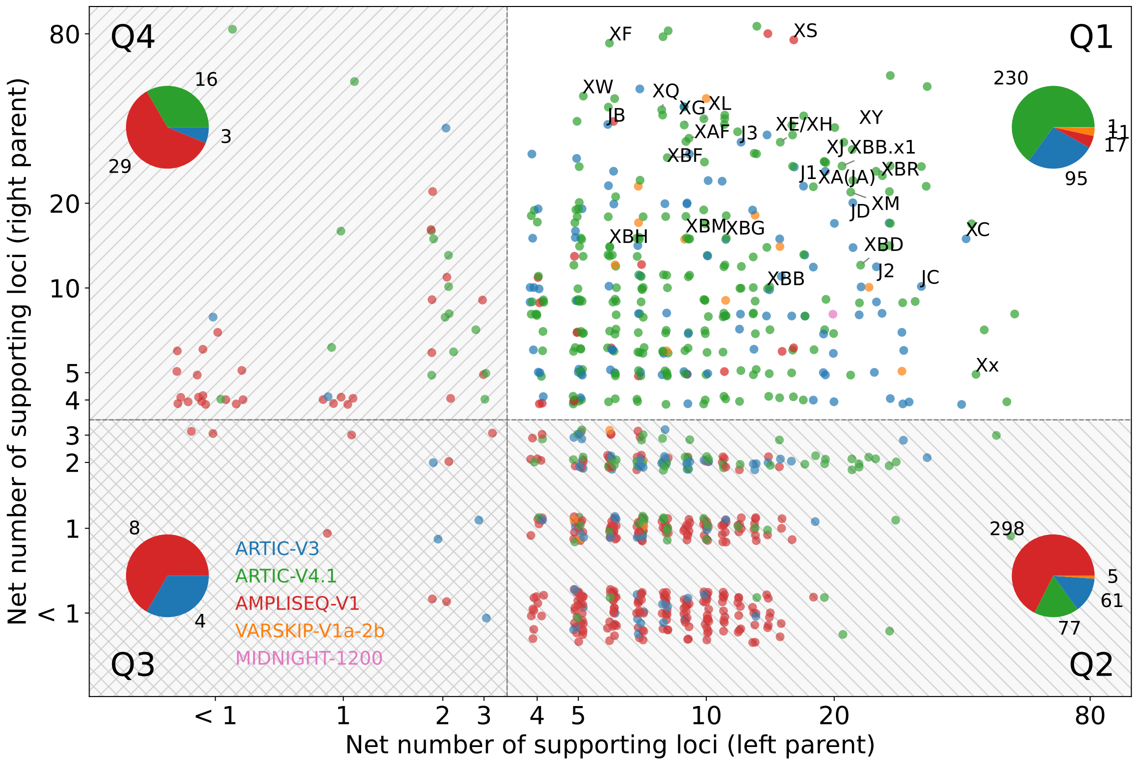

Figure S16: Quality control of recombination events. Scatterplot of recombination events by the net number of supporting loci on the left parent (x-axis) and the right parent (y-axis). The shaded regions highlight potentially artefactual recombination events, which have fewer than 4 net supporting loci on one side or both sides of the suggested breakpoint. colours indicate the primer scheme used for sequencing. The pie charts show breakdowns of the recombination events by primer scheme per quadrant (labeled Q1 to Q4). Recombination events associated with the origins of Pango X and Jackson[29] lineages are labeled.

bioRxiv preprint doi: https://doi.org/10.1101/2023.06.08.544212; this version posted November 10, 2025. The copyright holder for this preprint (which was not certified by peer review) is the author/funder, who has granted bioRxiv a license to display the preprint in perpetuity. It is made available under aCC-BY 4.0 International license.

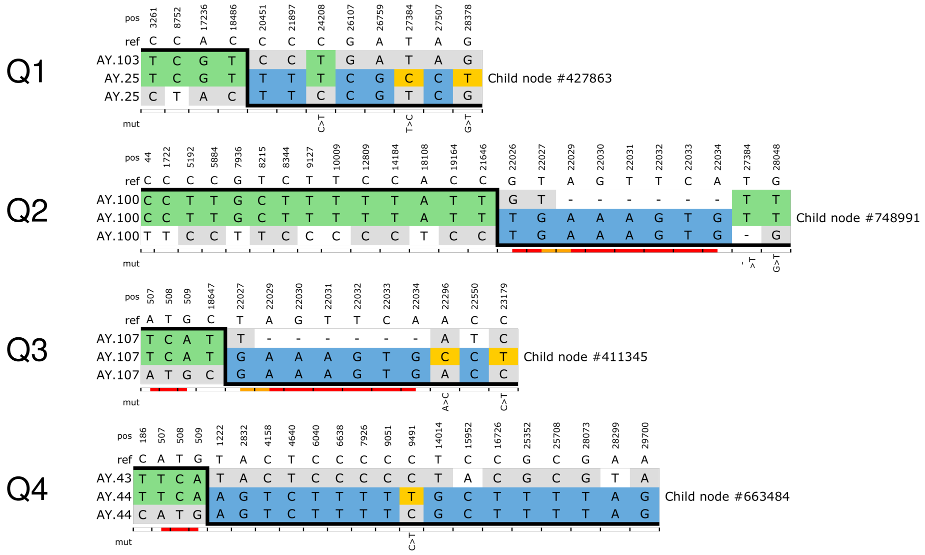

Figure S17: Illustrative copying patterns drawn from the quadrants labelled in Figure S16. Each copying pattern shows the the positions where the allelic state in a recombinant (middle row, labelled by node ID) matches that of the left (P0: upper row, coloured green) or right parent (P1: lower row, coloured blue), or where the recombination event requires a de-novo mutation (gold, with mutational change below). Parental states that correspond to the reference but are not inherited by the recombinant are shown with a gray background. Genome position ("pos") and reference allele ("ref") are shown for each column. Underneath the copying pattern, adjacent genomic positions are underlined in red, and near-adjacent sites (within 3 bases) in orange.

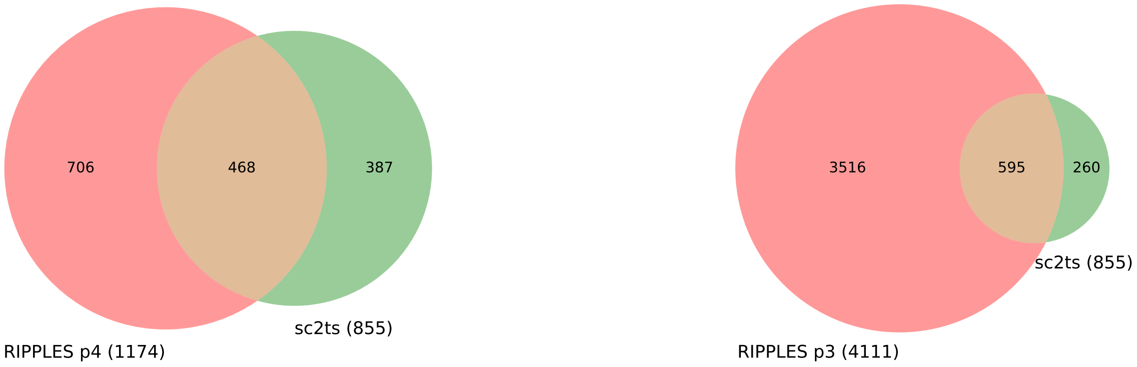

(A) (B)

Figure S18: Intersection of the RIPPLES and sc2ts recombination events at different values of the RIPPLES parsimony parameter, p.

bioRxiv preprint doi: https://doi.org/10.1101/2023.06.08.544212; this version posted November 10, 2025. The copyright holder for this preprint (which was not certified by peer review) is the author/funder, who has granted bioRxiv a license to display the preprint in perpetuity. It is made available under aCC-BY 4.0 International license.

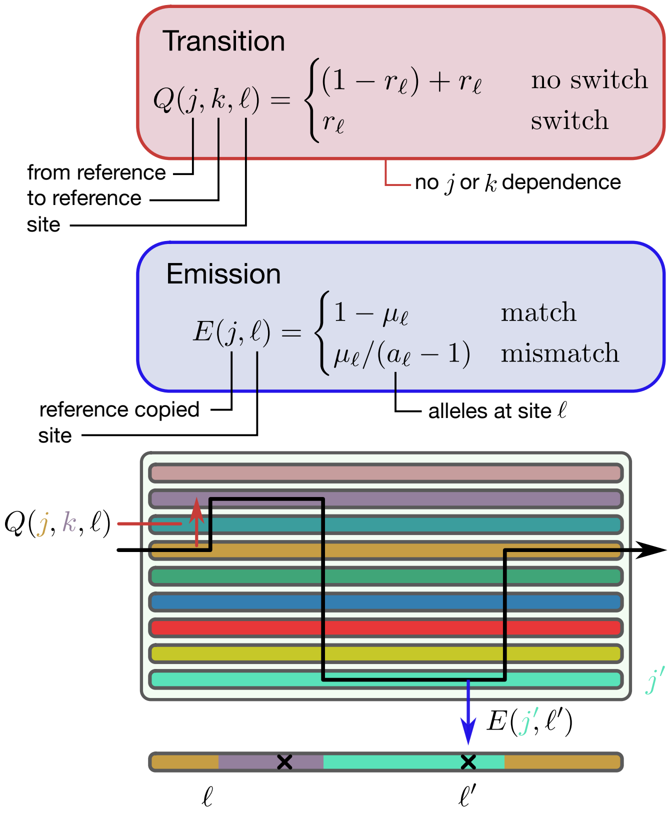

Figure S19: A schematic of the Li and Stephens (LS) model, in which a focal sequence (bottom) is described as an imperfect mosaic of the sequences in a reference panel. Black crosses along the focal sequence show sequencing errors or mutations. In the standard formulation, at site ℓ, the recombination probability is r_ℓ, the mutation probability is μ_ℓ and n denotes the size of the reference panel. The Viterbi algorithm can be used to find a "copying path" through the reference panel for a given focal sequence that maximises the likelihood under these parameters. Unseen states in the reference panel are shown as coloured lines enclosed by the grey box. The black arrow describes the true path through the data which leads to the emitted focal sequence below. Examples of transition and emission probabilities along this trajectory are shown by the red and blue arrows, respectively.

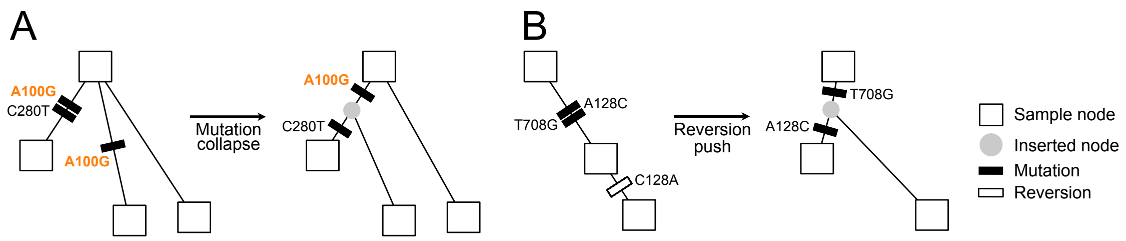

Figure S20: Parsimony improving heuristics. (A) Mutation collapsing. Mutation A100G is shared by two siblings, and we create a new node to represent the ancestor on which this mutation occurred. (B) Reversion pushing. Mutation A128C is immediately reverted by C128A, and we create a new node to represent the ancestor that did not carry A128C.
